# Supplementary material for: Inventa: A computational tool to discover structural novelty in natural extracts libraries
Source: Front Mol Biosci. 2022 Nov 11;9:1028334. doi: 10.3389/fmolb.2022.1028334 (PMC9692083; doi:10.3389/fmolb.2022.1028334)
Supplement: Supplementary file 1 [file DataSheet1.PDF]

Supporting Information

Inventa: a computational tool to discover chemical novelty in natural extracts libraries

Luis-Manuel Quiros-Guerrero[1,2,*], Louis-Félix Nothias[1,2], Arnaud Gaudry[1,2], Laurence Marcourt[1,2], Pierre-Marie Allard[1,2,3], Adriano Rutz[1,2], Bruno David[4], Emerson Ferreira Queiroz[1,2], Jean-Luc Wolfender[1,2*]

Supplementary Table S1. Glossary of terms and default values

**Parameter**	**Default value**	**Description**
annot_gnps_df		raw annotations attributes file downloaded directly from GNPS
annot_is_df		annotation status table from in silico ISDB dereplication
annot_sirius_df		annotation status table from SIRIUS dereplication
annotation_df		combined annotation status table from all the dereplication results used
annotation_preference	0	Only Annotated features: '1' or Only Not annotated features: '0' #userdefined
ATTRIBUTE_Sppart'		if needed this column is generated by merging the species and organe columns #userdefined
canopus_npc_df		chemical classes retrieved from the Sirius dereplication results
canopus_npc_summary_filename	../data/yourfilenamegoeshere.tsv'	path where the SIRIUS-CANOPUS results file is placed
CC		Class component results table
CC_component	True (False)	CC will be calculated
col_id_unique		unique identifier for the samples (filename, part, etc) #userdefined
correlation_groups_df		ion identity annotation network number attributes
data_process_origin	MZmine3' / 'MZmine2'	version of MZmine used to treat the data #userdefined
df_annotations		raw annotations file downloaded directly from GNPS

family_colum	ATTRIBUTE_Family' (yourfamilycolumgoeshere)	column header for the family name #userdefined
FC		Feature component results table
filename_header	filename' (yourfilenamecolumgoeshere)	column header for the species name #userdefined
filtered_F		the residual number of features different from zero in the quant_df after filtering
filtered_full_df		filtered (without QC and blanks) full table
filtered_metadata_df		filtered (without QC and blanks) metadata table
FS		Feature Specificity, ratio of Total_SF/filtered_F
full_df		quantitative and metadata combined table
genus_colum	ATTRIBUTE_Genus' (yourgenuscolumgoeshere)	column header for the genus name #userdefined
gnps_annotations_consolidated		processed annotations file from GNPS
gnps_annotations_filtered		processed annotations file from GNPS after chemical descriptors consolidation
IF		Isolation Forest
initial_F		the initial number of features different from zero in the quant_df
ionisation_mode	pos'	ionisation mode according to experimental conditions 'pos' or 'neg' #userdefined
isdb_annotations	True (False)	the tima_results_filename will be considered in the calculations #userdefined
job_id	yourjobidgoeshere	GNPS Job ID #userdefined
LC		Literature component results table
LC_component	True (False)	LC will be calculated
LOF		Local Outlier Factor
max_comp_reported_f	500	max number of compounds reported at genus level,more than this value, the plant is considered less interesting #userdefined
max_comp_reported_g	50	max number of compounds reported at genus level,more than this value, the plant is considered less interesting #userdefined
max_comp_reported_sp	20	max number of compounds reported at species level, more than this value, the plant is considered less interesting #userdefined

max_parts_per_organism	4 (your max occurrence goes here)	max recurrence of the same organism species (for example: 5 samples, same species but different plant part) #userdefined
max_ppm_error	5	min error in ppm to consider an annotation valable #userdefined
max_spec_charge	2	maximum charge allowed #userdefined
metadata_df		formated metadata table
metadata_filename	../data/yourfilenamegoeshere.tsv'	path where the metadata is placed #userdefined
metric_df		memo matrix table
MF_prediction_ratio		Ratio of SNAGQMFF/filtered_F
min_class_confidence	0.8	cut-off filter for considering a sirius class valable. It is used in combination with min_recurrence #userdefined
min_ConfidenceScore	0.25	cut-off filter for considering a sirius annotation valable. '0.0' as default. #userdefined
min_cosine	0.6	min cosine score to consider an annotation valable #userdefined
min_recurrence	5	minimum recurrence of a chemical class to consider it acceptable #userdefined
min_score_final	0.3	cut-off filter for considering an isdb annotation valable. You must be extremenly carefull with this parameter, '0.0' as default. #userdefined
min_specificity	0.9	minimum value of relative area (0 to 1) to consider a feature specific or not #userdefined
min_ZodiacScore	0.9	cut-off filter for considering a sirius annotation valable. It is used in combination with min_ConfidenceScore. #userdefined
multiple_organism_parts	True (False)	True: the specificity is going to be considered as the sum of the 'max_parts_per_organism' shared in the samples. #userdefined
OCSVM		One Class Support Vector Machine
organe_colum	ATTRIBUTE_Organ' (yourorgancolumgoeshere)	column header for the organe name (this column could be used to indicate different culture media, extraction solvents, etc.) #userdefined
PR		Priority Rank results table
quant_df		sample-wise normalized quantitative table
quantitative_data_filename	../data/yourfilenamegoeshere.tsv'	path where the quantitative table is placed #userdefined
reduced_df		col_id_unique restricted full df
Reported_comp_Family		Number of compounds reported in the family in Lotus

Reported_comp_Genus		Number of compounds reported in the genus in Lotus
Reported_comp_Species		Number of compounds reported in the species in Lotus
SC		Similarity component results table
SC_component	True (False)	SC will be calculated
shared_peaks	4	min number of shared peaks between the MS2 experimental and MS2 from the database, to consider an annotation valable #userdefined
sirius_annotations	True (False)	the sirius_annotations_filename will be considered in the calculations
sirius_annotations_filename	../data/yourfilenamegoeshere.tsv'	path where the SIRIUS results file is placed #userdefined
species_colum	ATTRIBUTE_Species' (yourspeciescolumgoeshere)	column header for the species name #userdefined
tima_results_filename	../data/yourfilenamegoeshere.tsv'	path where the ISDB reponderated file is placed #userdefined
Total_SF		the total number of Specific (S) features (F)
Total_SNA_GQMFF		the total number of Specific (S) non-annotated (NA) features (F) with a molecular formula of good quality (GQMF)
Total_SNAF		the total number of Specific (S) non-annotated (NA) features (F)
use_ion_identity	True (or False)	if True, the ion identity results will be used #userdefined
vectorized_data_filename	../data/yourfilenamegoeshere.tsv'	path where the MEMO matrix is placed #userdefined
w1	1	weight for the FC in the final PR result #userdefined
w2	1	weight for the LC in the final PR result #userdefined
w3	1	weight for the SC in the final PR result #userdefined
w4	1	weight for the CC in the final PR result #userdefined
wf	1	weight for the family level in the LC component #userdefined
wg	1	weight for the genus level in the LC component #userdefined
ws	1	weight for the species level in the LC component #userdefined

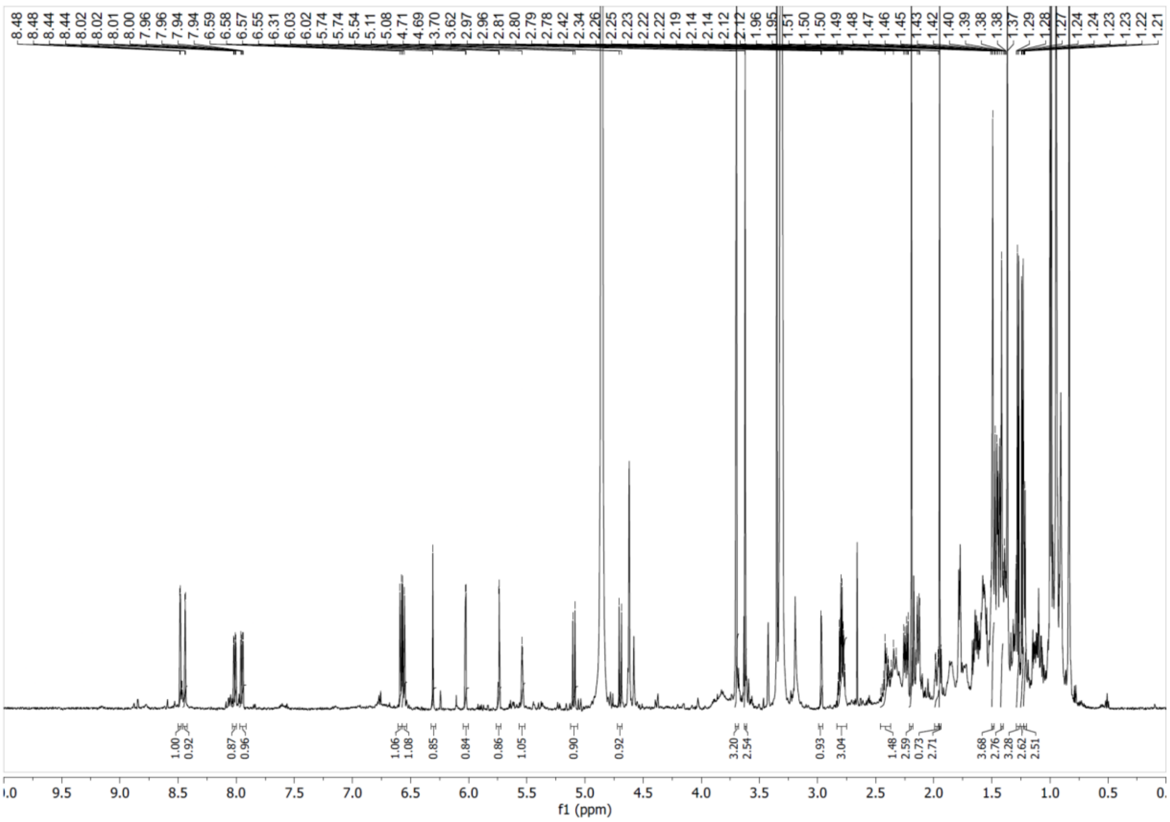

Supplementary Figure S1. ^1H NMR spectrum of compound **1** in CD_3OD at 600 MHz.

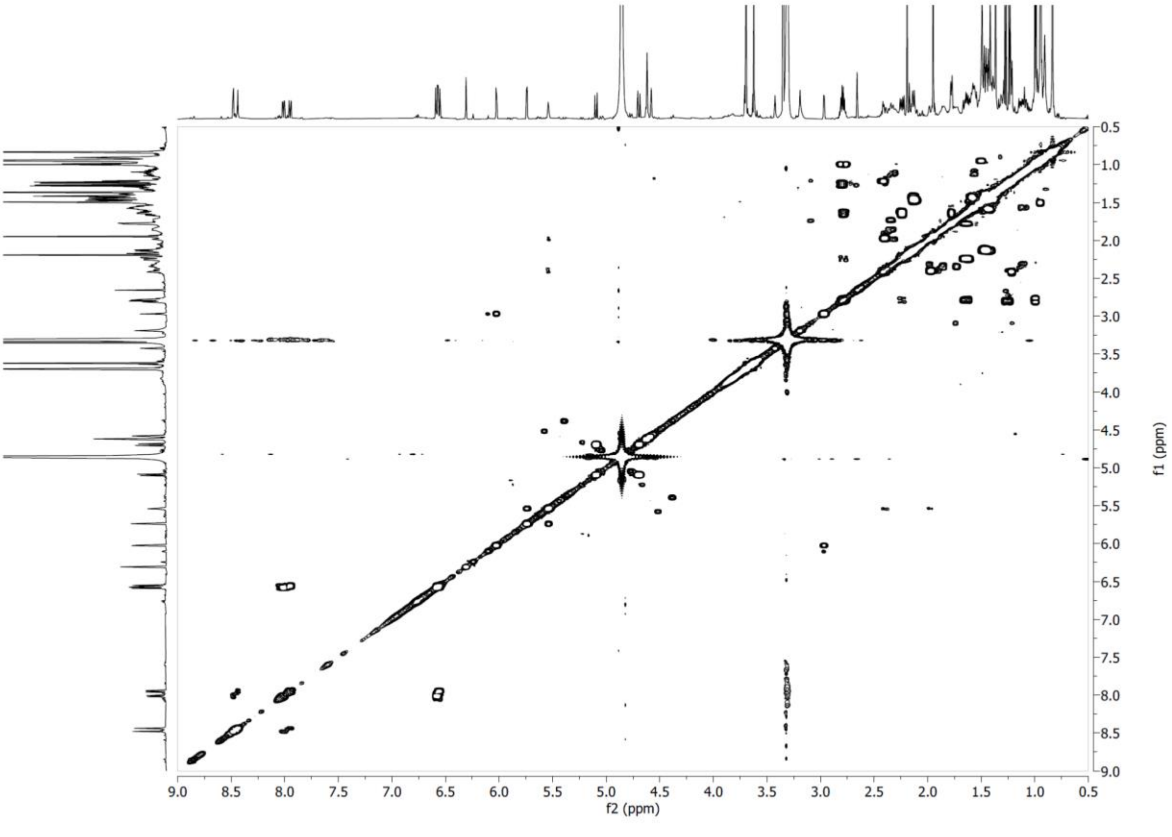

Supplementary Figure S2. COSY NMR spectrum of compound **1** in CD_3OD at 600 MHz.

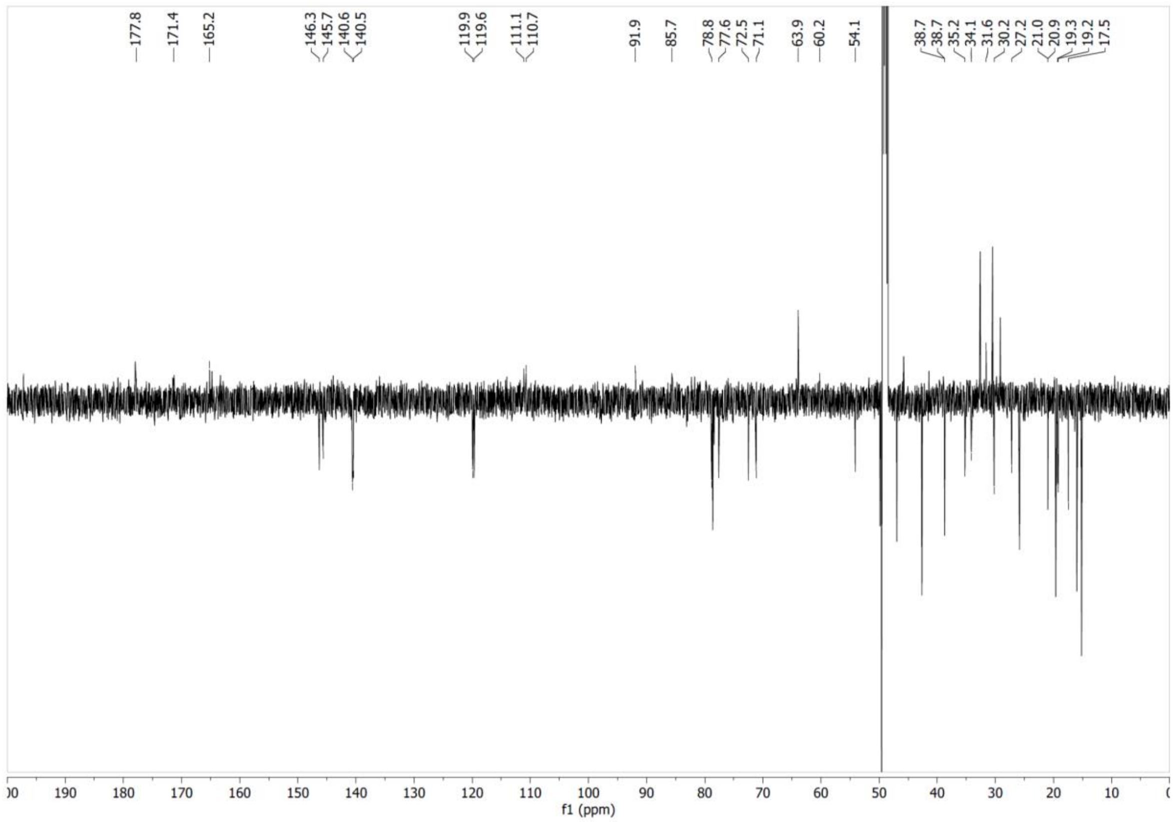

Supplementary Figure S3. ^{13}C NMR spectrum of compound **1** in CD_3OD at 151 MHz.

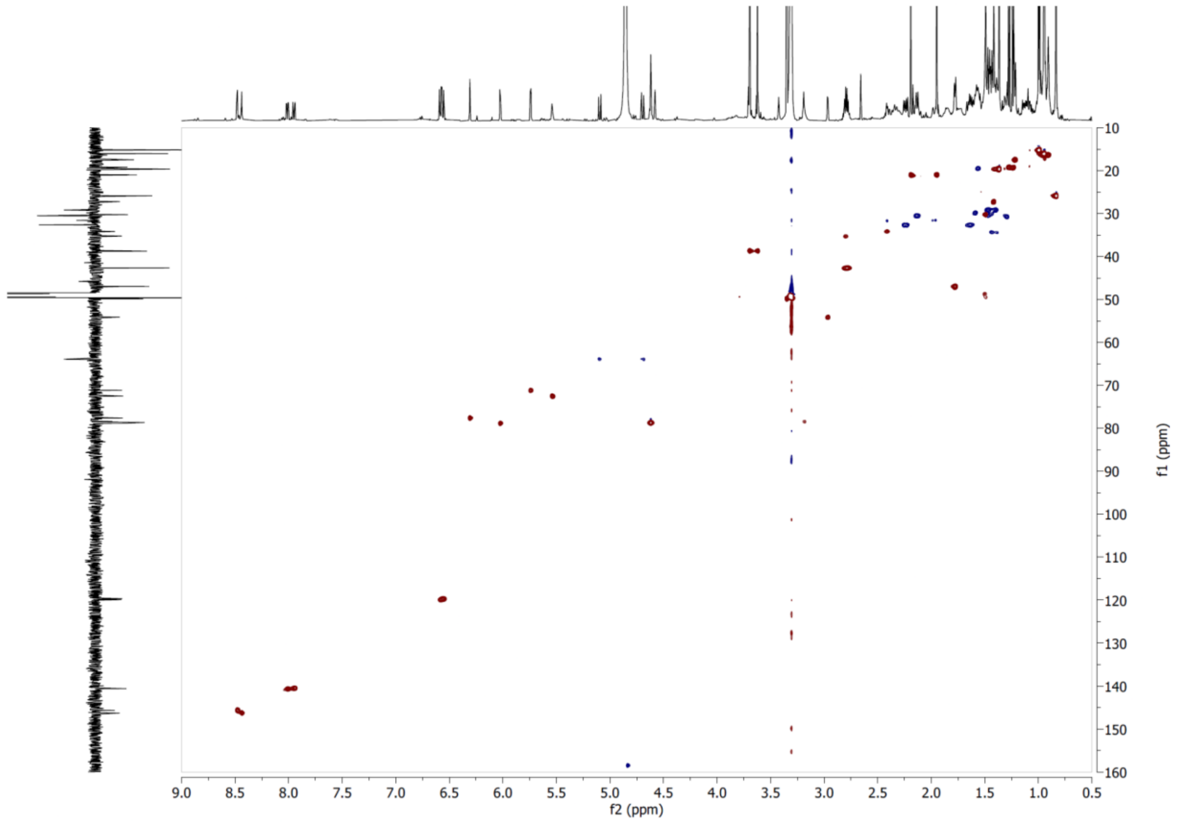

Supplementary Figure S4. HSQC NMR spectrum of compound **1** in CD_3OD.

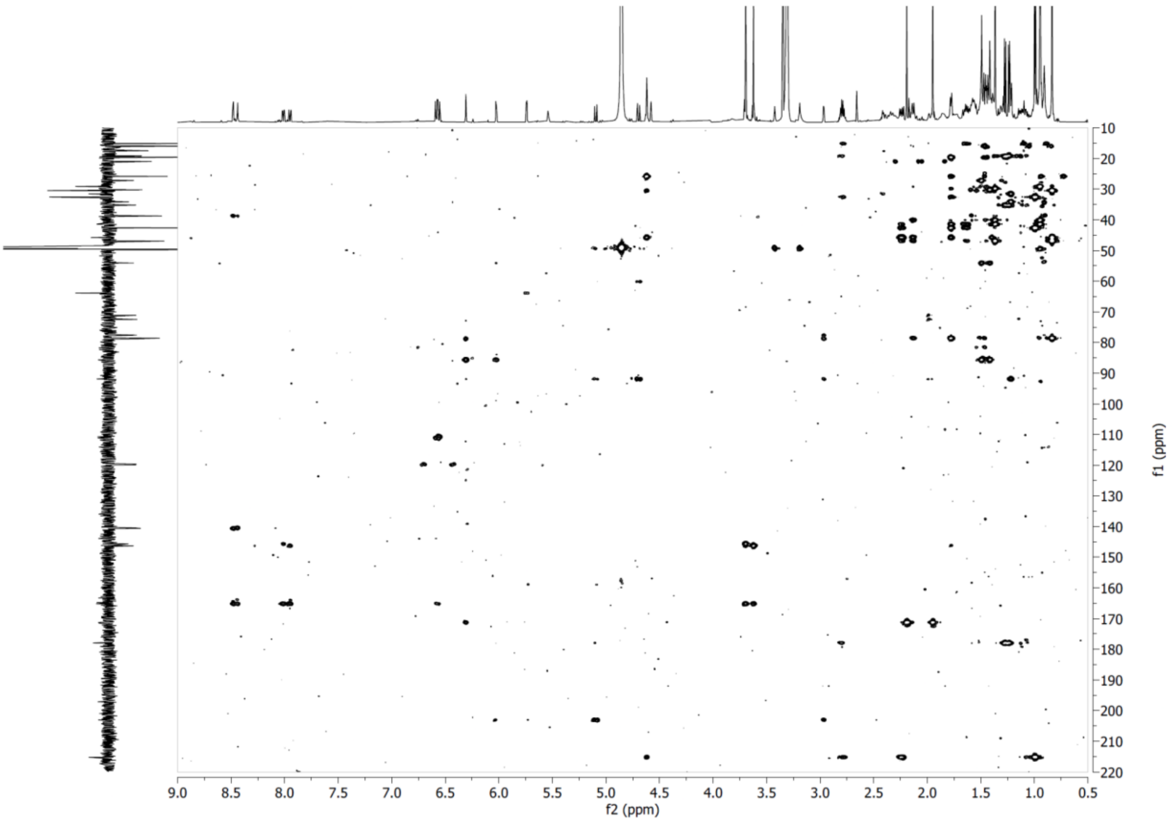

Supplementary Figure S5. HMBC NMR spectrum of compound **1** in CD_3OD.

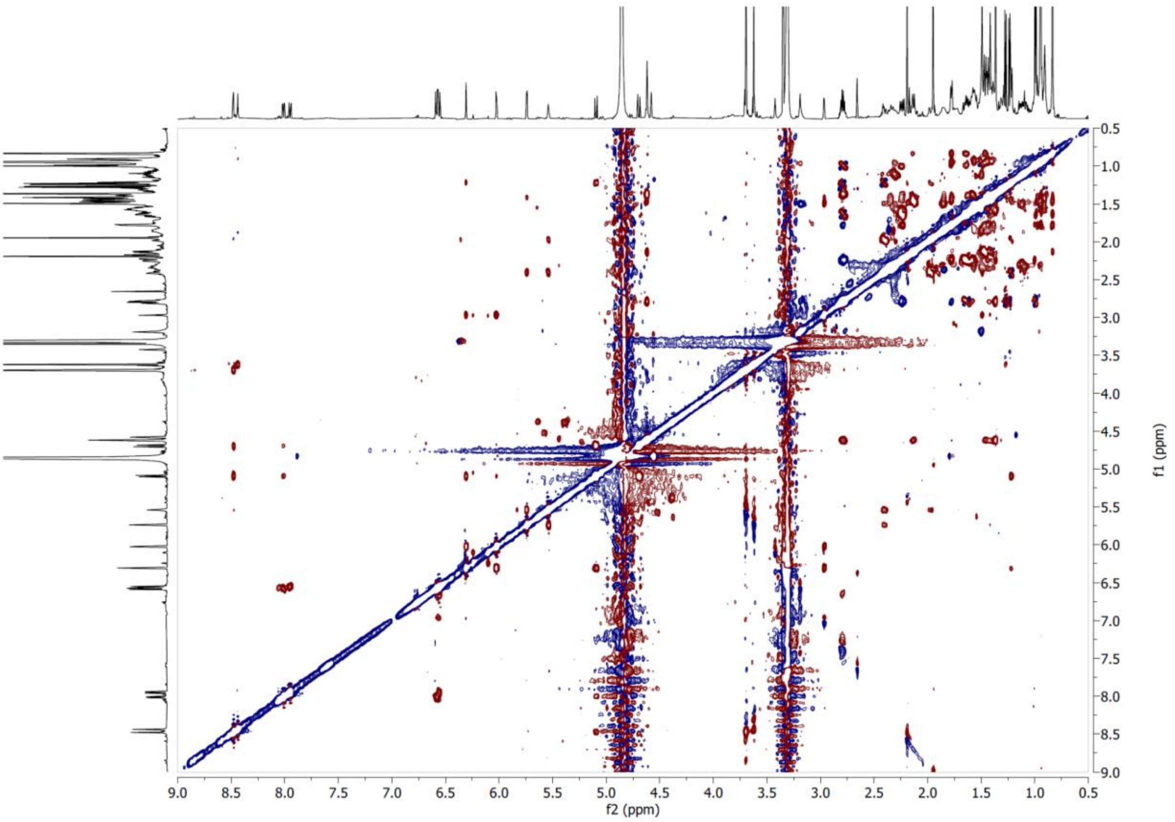

Supplementary Figure S6. ROESY NMR spectrum of compound **1** in CD_3OD at 600 MHz.

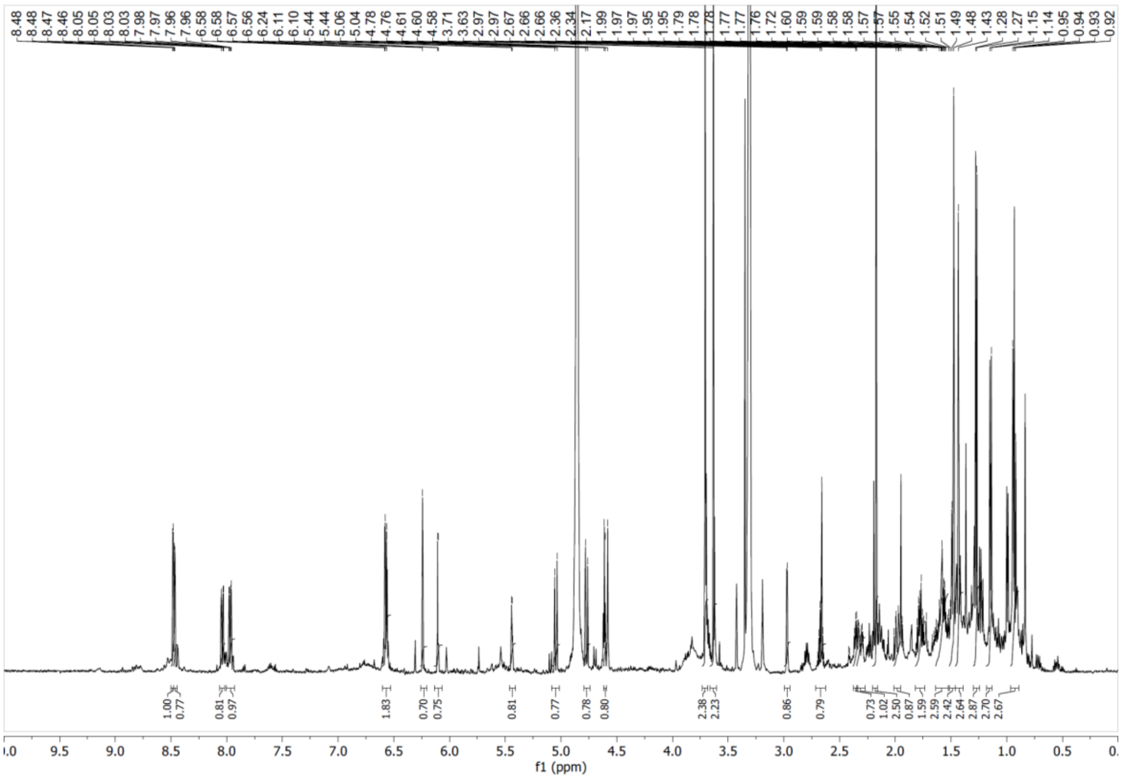

Supplementary Figure S7. ^1H NMR spectrum of compound **2** in CD_3OD at 600 MHz.

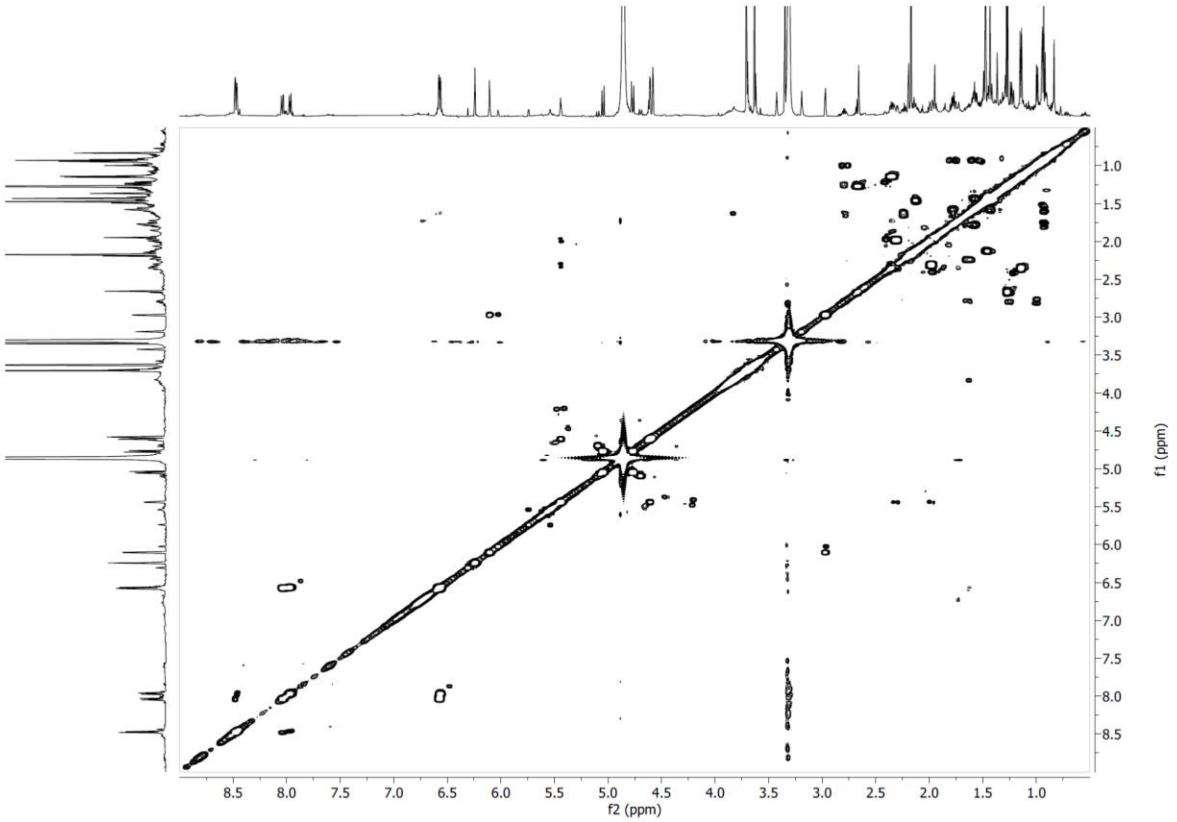

Supplementary Figure S8. COSY NMR spectrum of compound **2** in CD_3OD at 600 MHz.

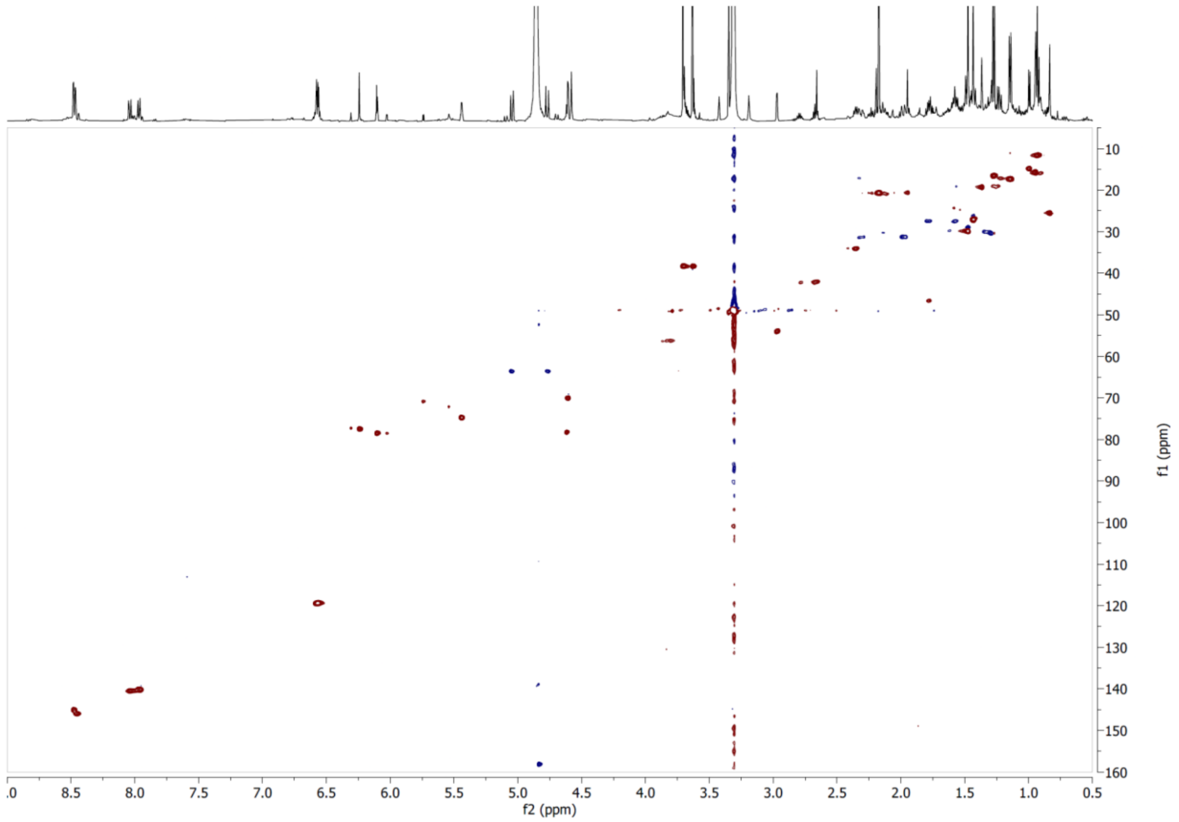

Supplementary Figure S9. HSQC NMR spectrum of compound **2** in CD_3OD.

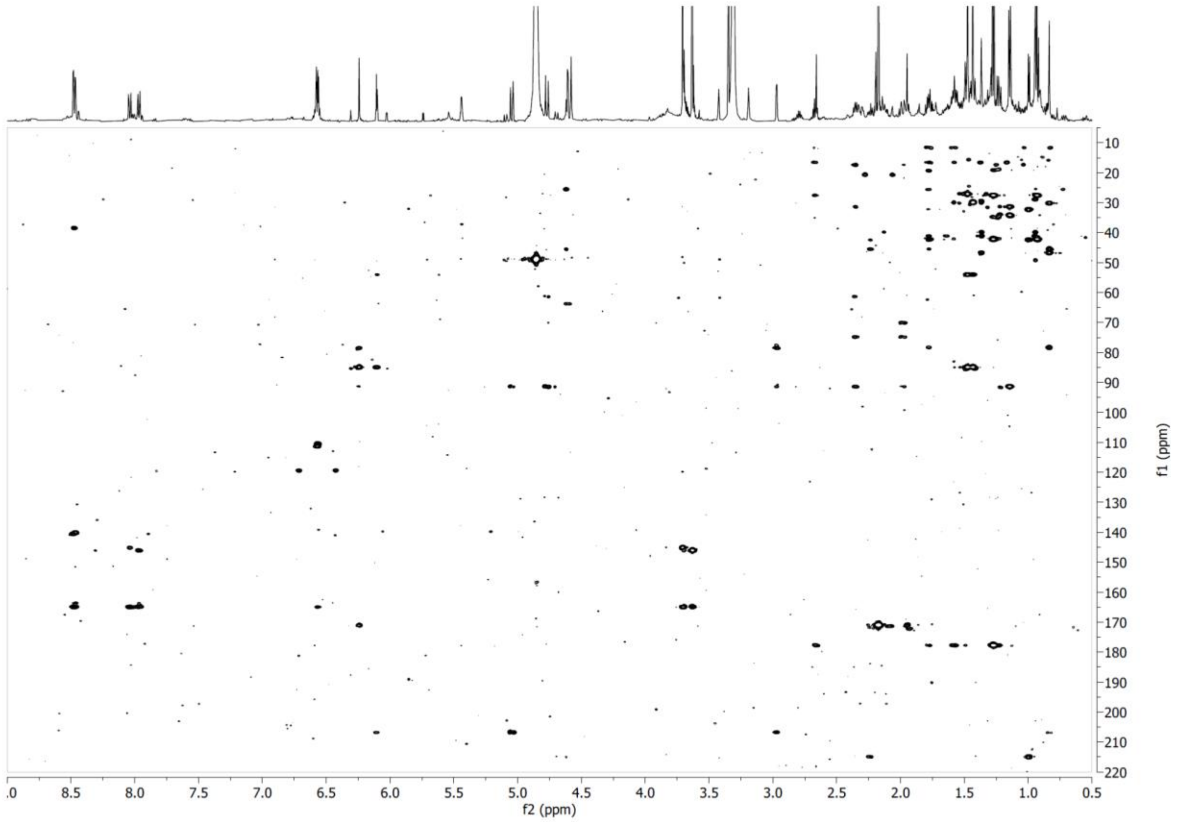

Supplementary Figure S10. HMBC NMR spectrum of compound **2** in CD_3OD.

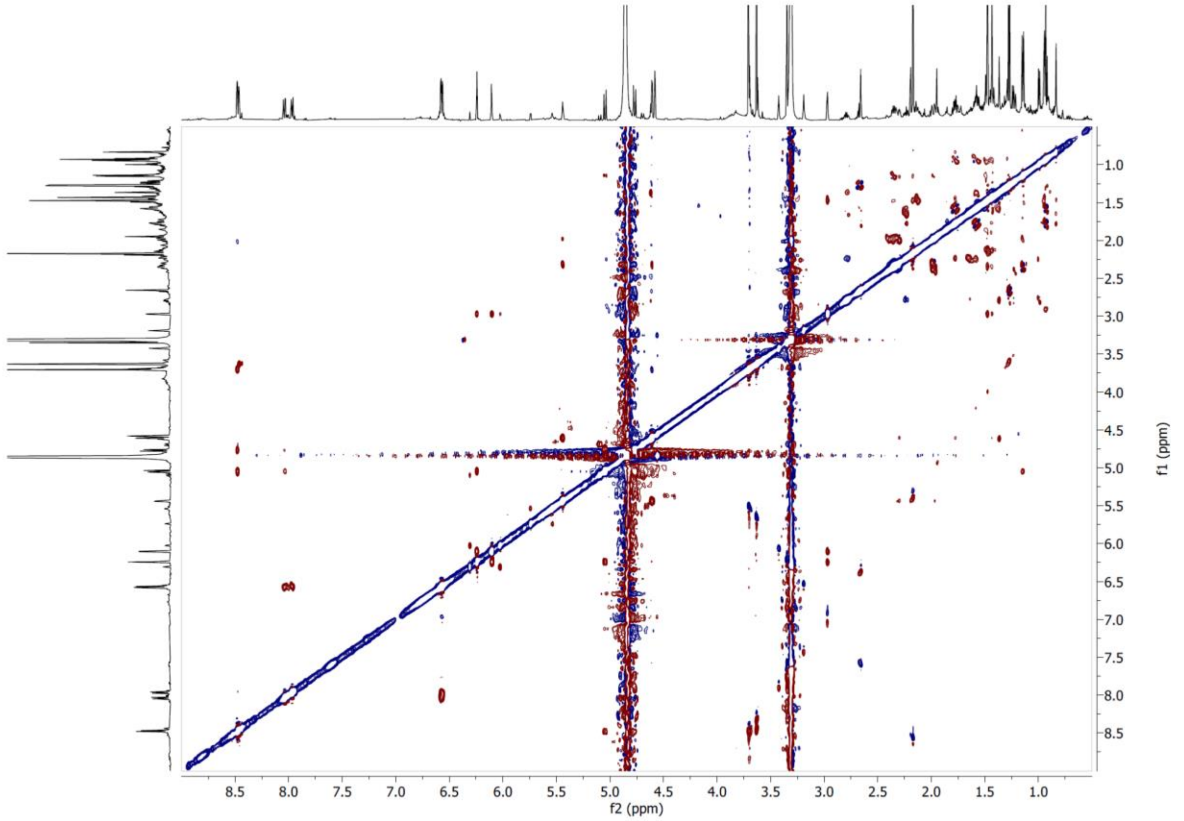

Supplementary Figure S11. ROESY NMR spectrum of compound **2** in CD_3OD at 600 MHz.

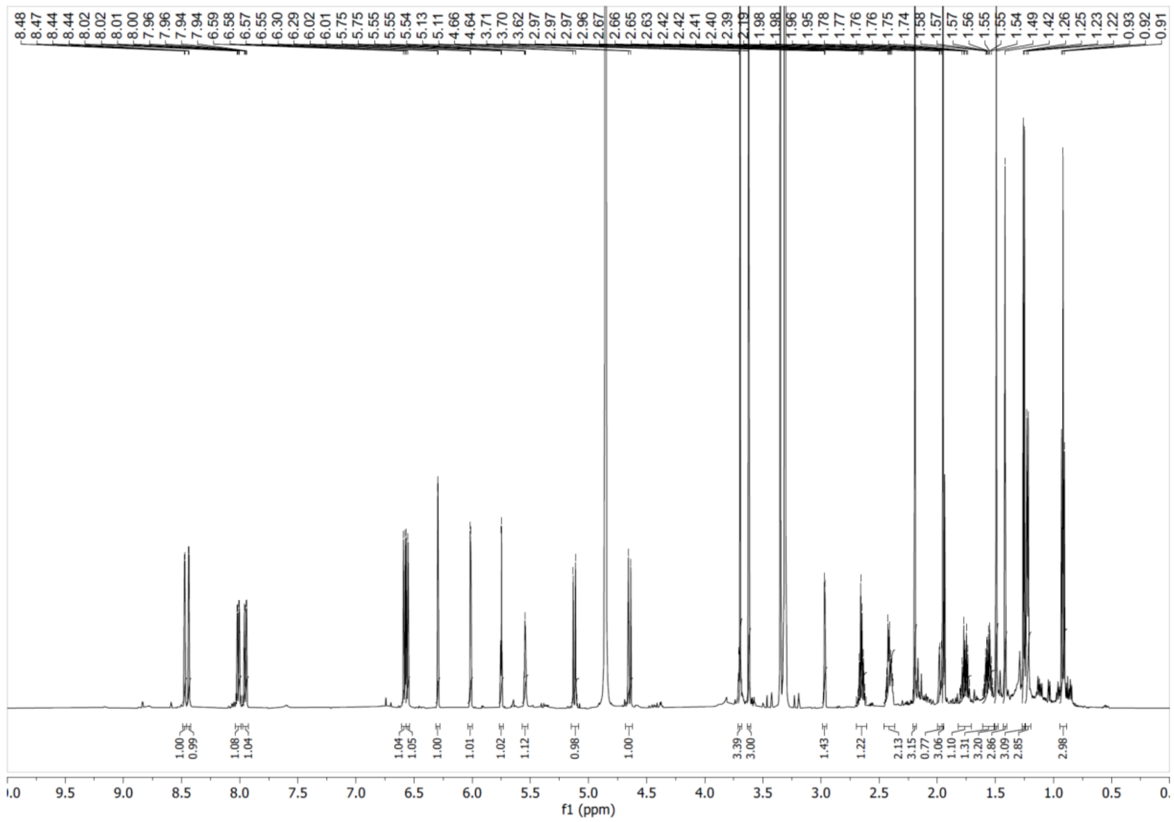

Supplementary Figure S12. ^{1}H NMR spectrum of compound **3** in CD_3OD at 600 MHz.

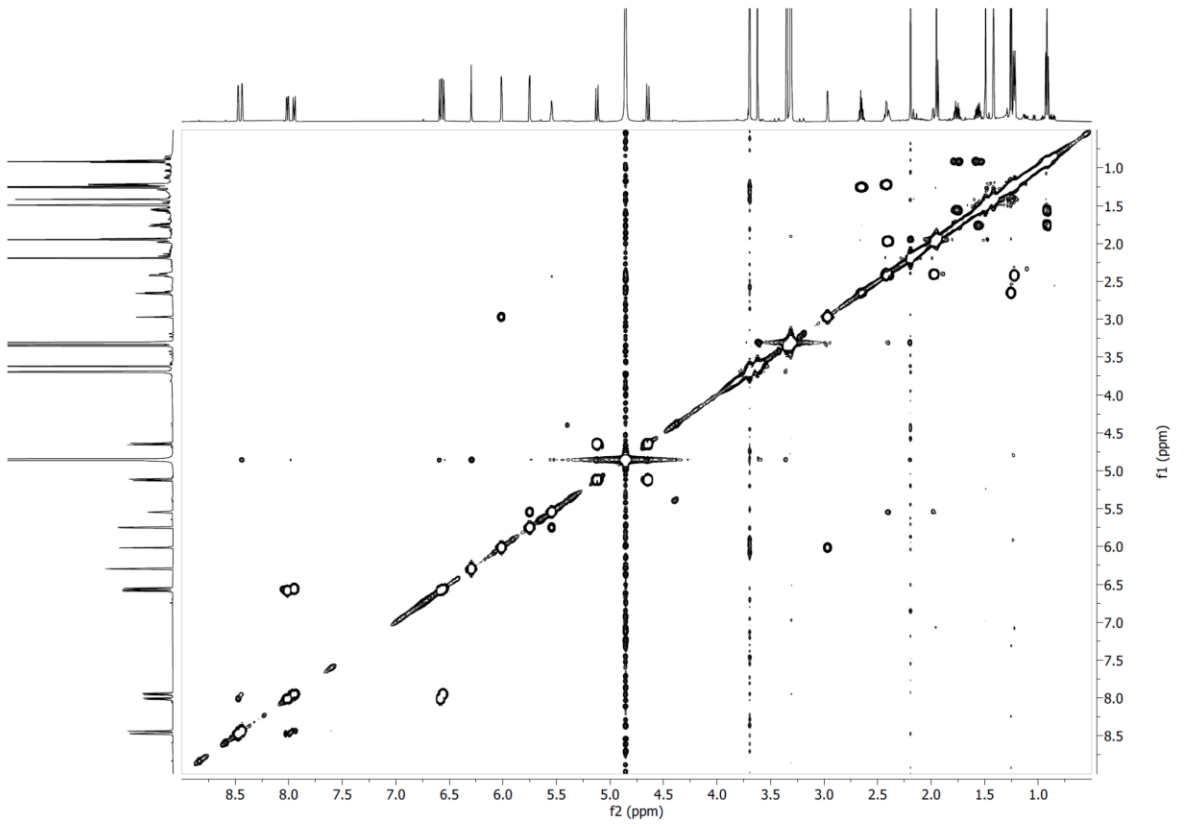

Supplementary Figure S13. COSY NMR spectrum of compound **3** in CD_3OD at 600 MHz.

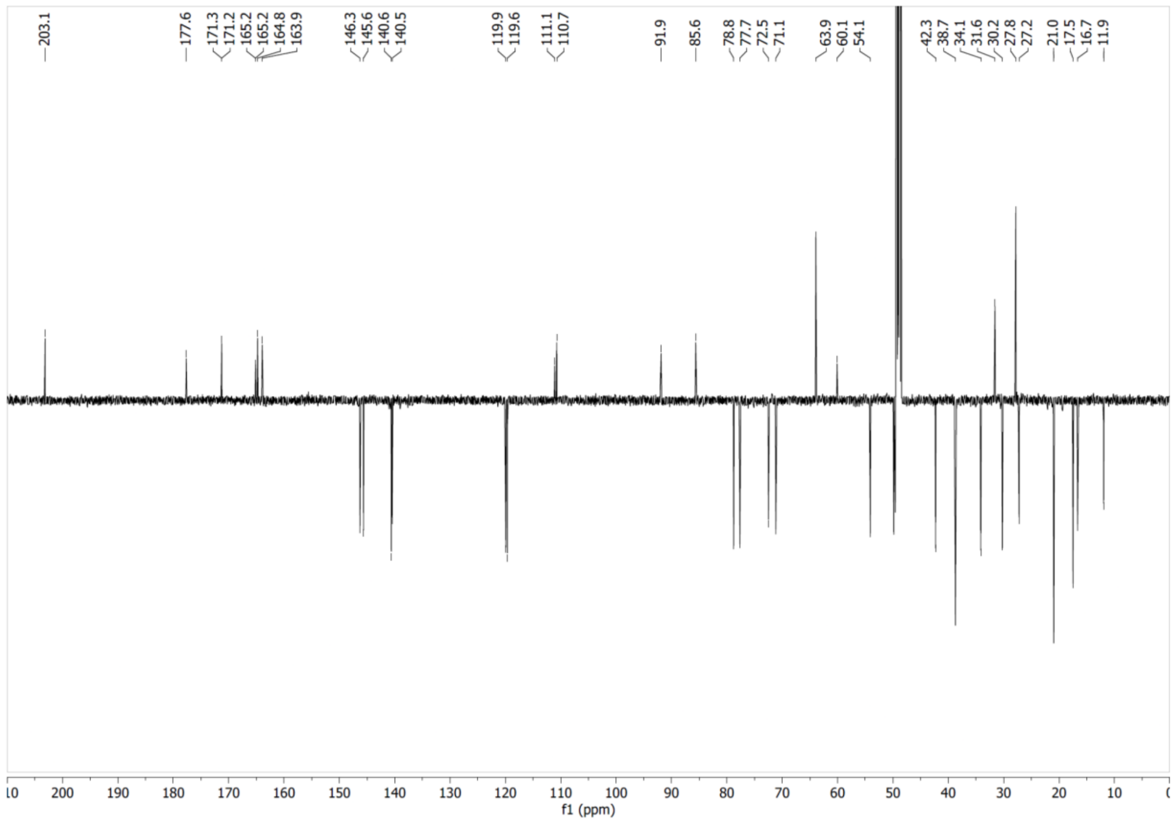

Supplementary Figure S14. ^{13}C NMR spectrum of compound **3** in CD_3OD at 151 MHz.

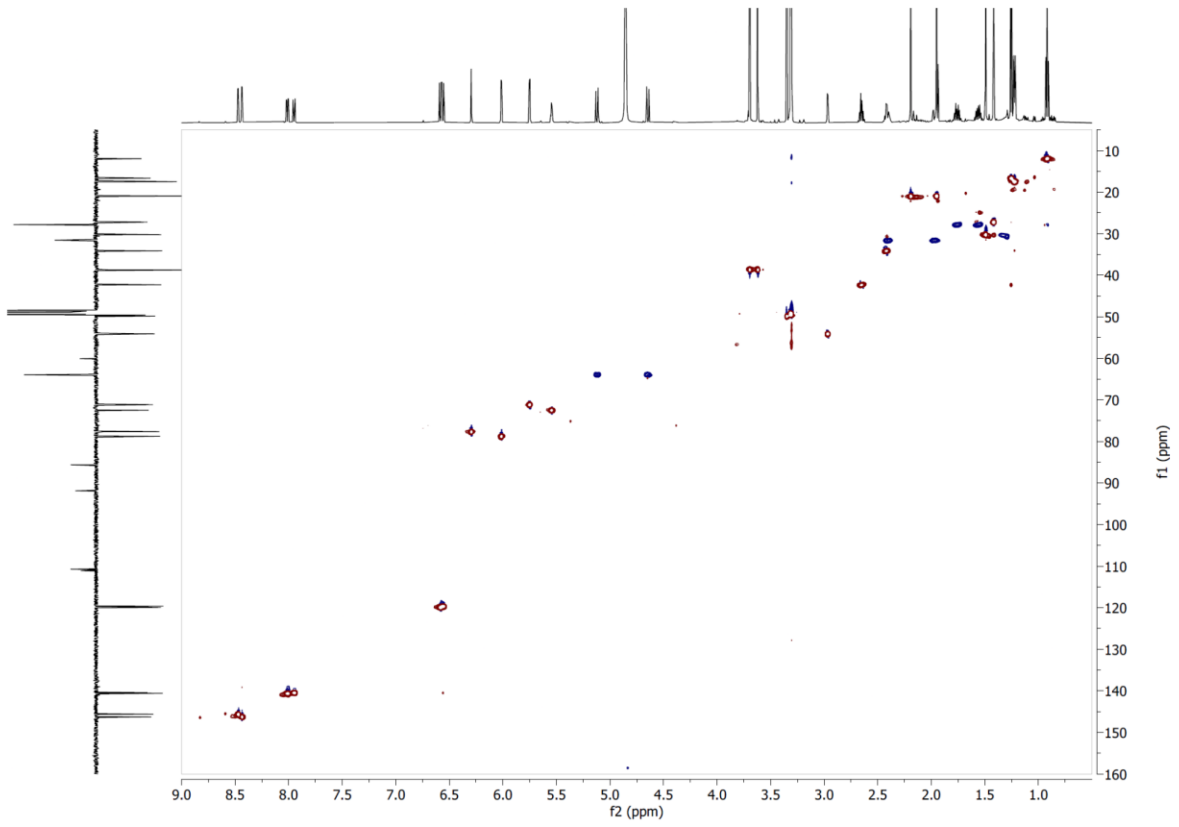

Supplementary Figure S15. HSQC NMR spectrum of compound **3** in CD_3OD.

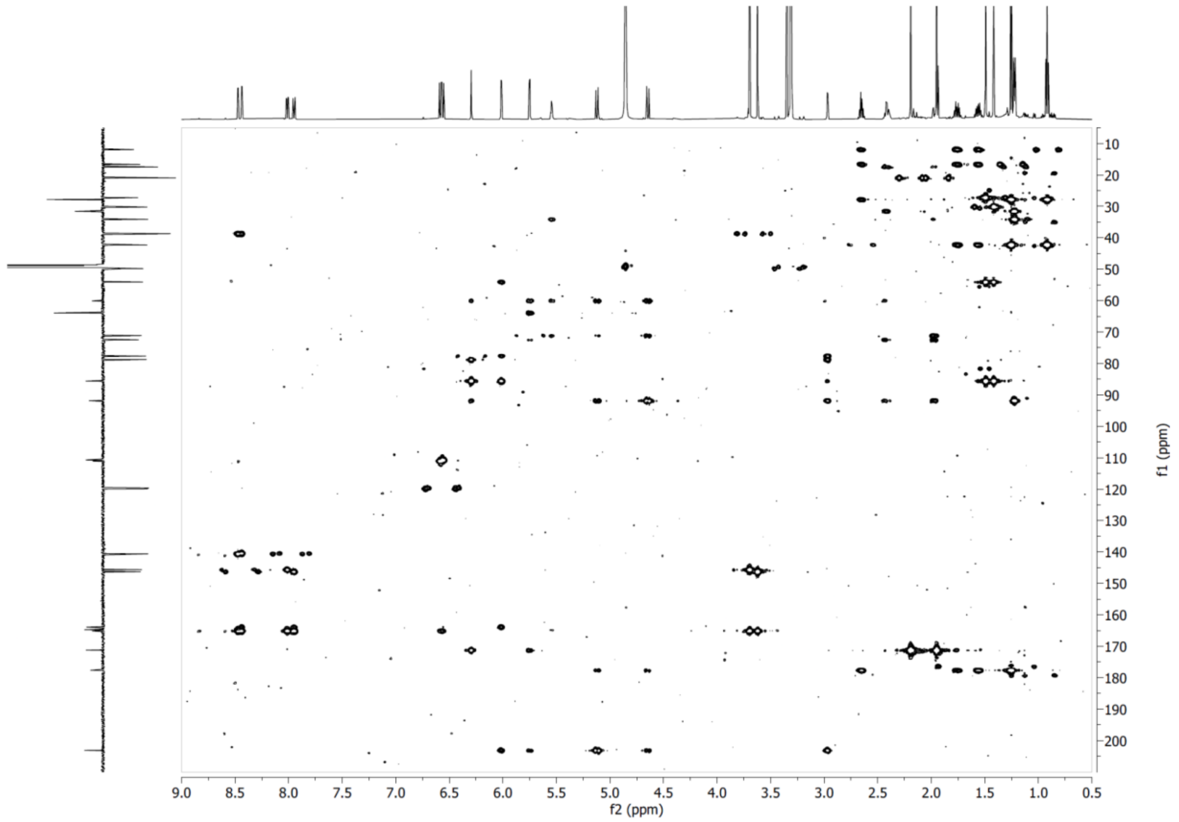

Supplementary Figure S16. HMBC NMR spectrum of compound **3** in CD_3OD.

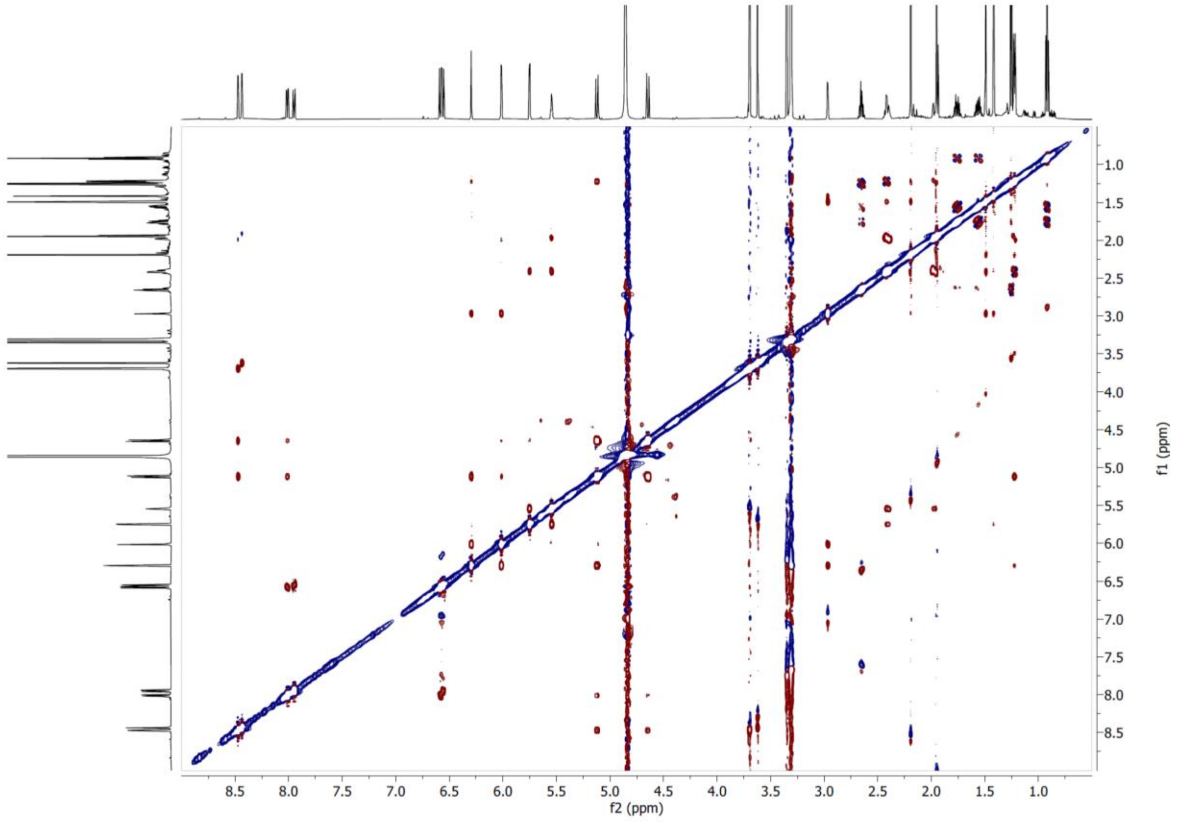

Supplementary Figure S17. ROESY NMR spectrum of compound **3** in CD_3OD at 600 MHz.

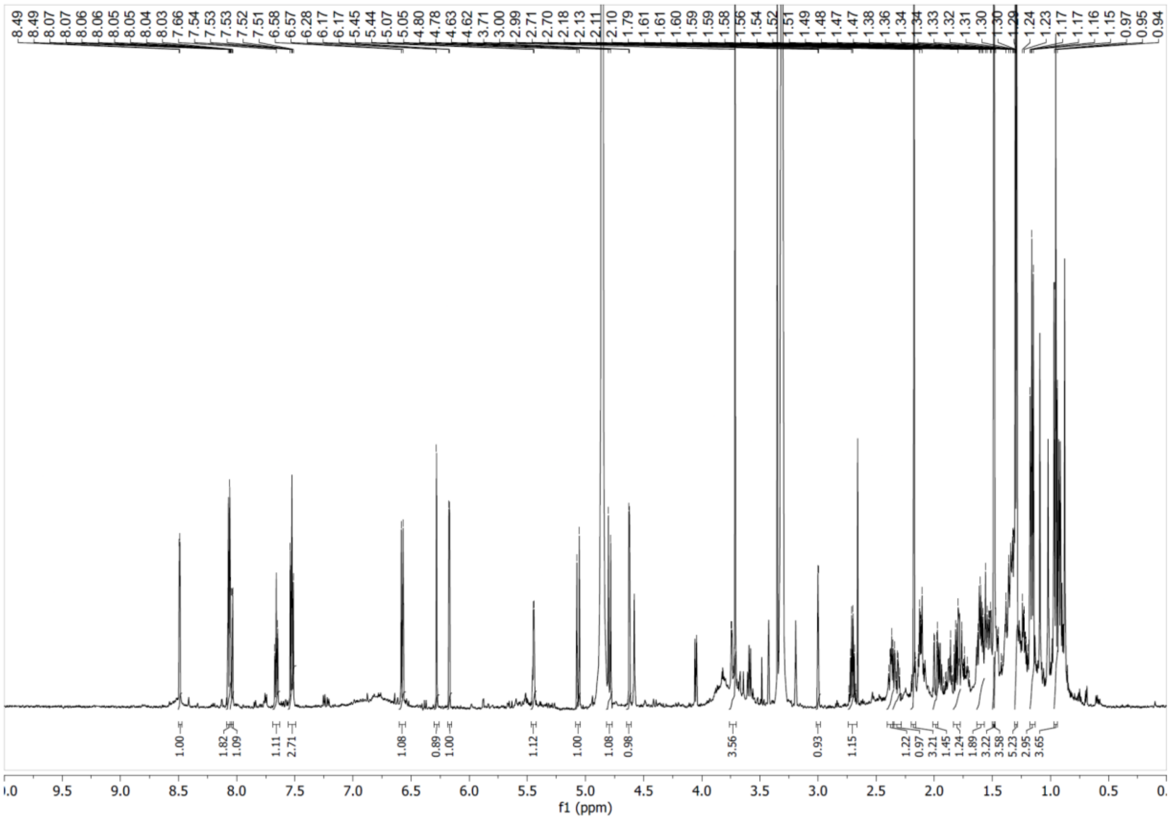

Supplementary Figure S18. ^{1}H NMR spectrum of compound **4** in CD_3OD at 600 MHz.

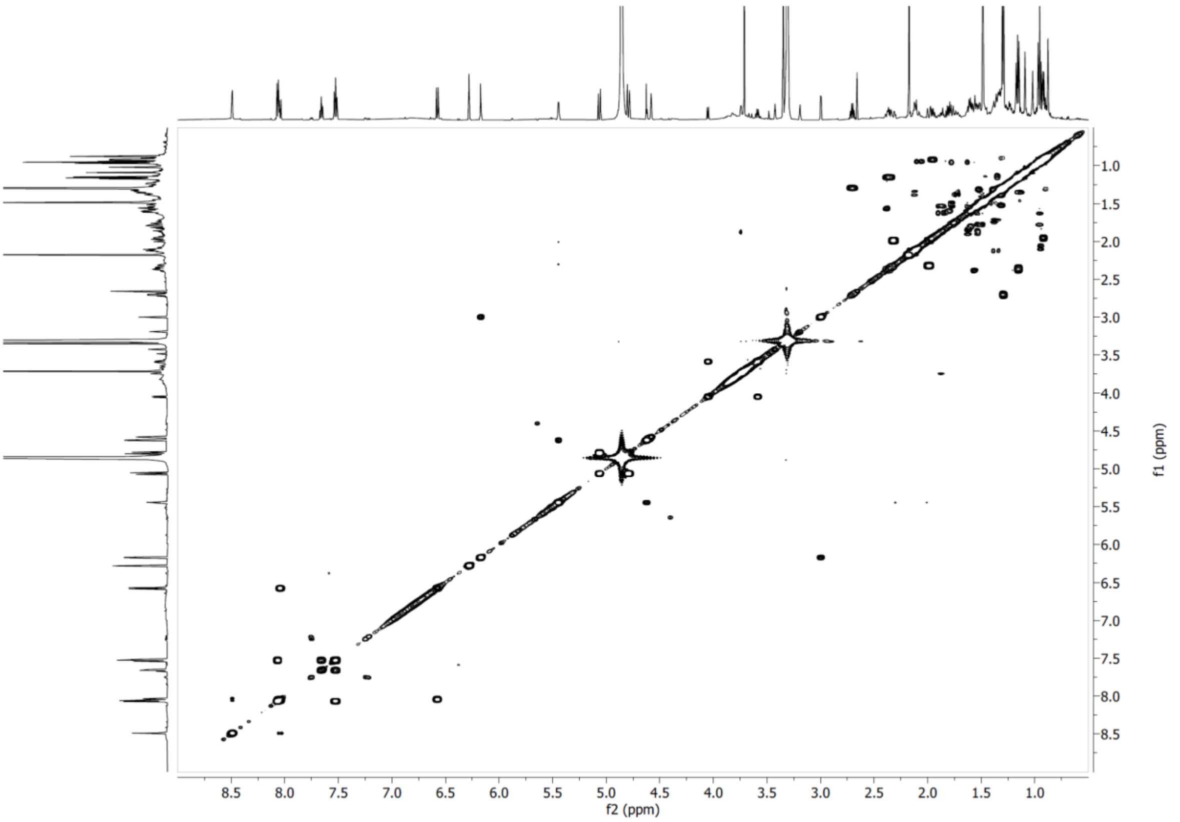

Supplementary Figure S19. COSY NMR spectrum of compound **4** in CD_3OD at 600 MHz.

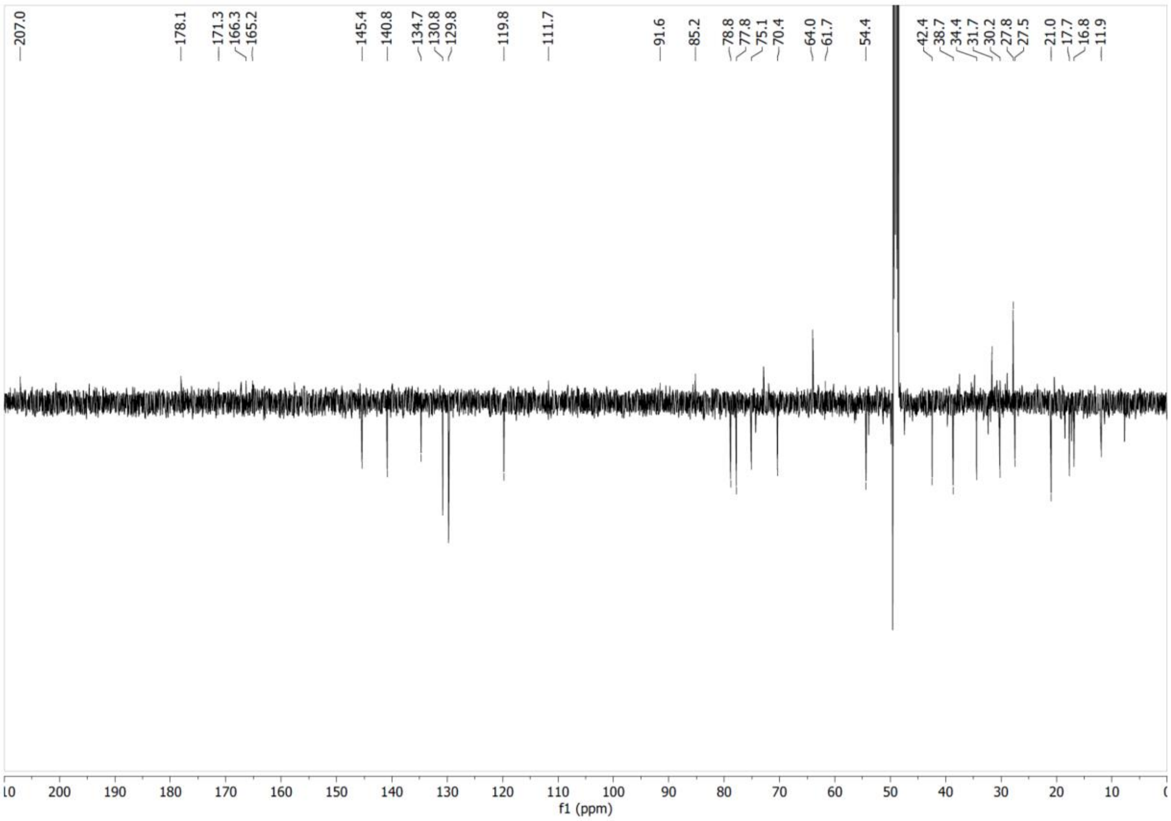

Supplementary Figure S20. ^{13}C NMR spectrum of compound **4** in CD_3OD at 151MHz.

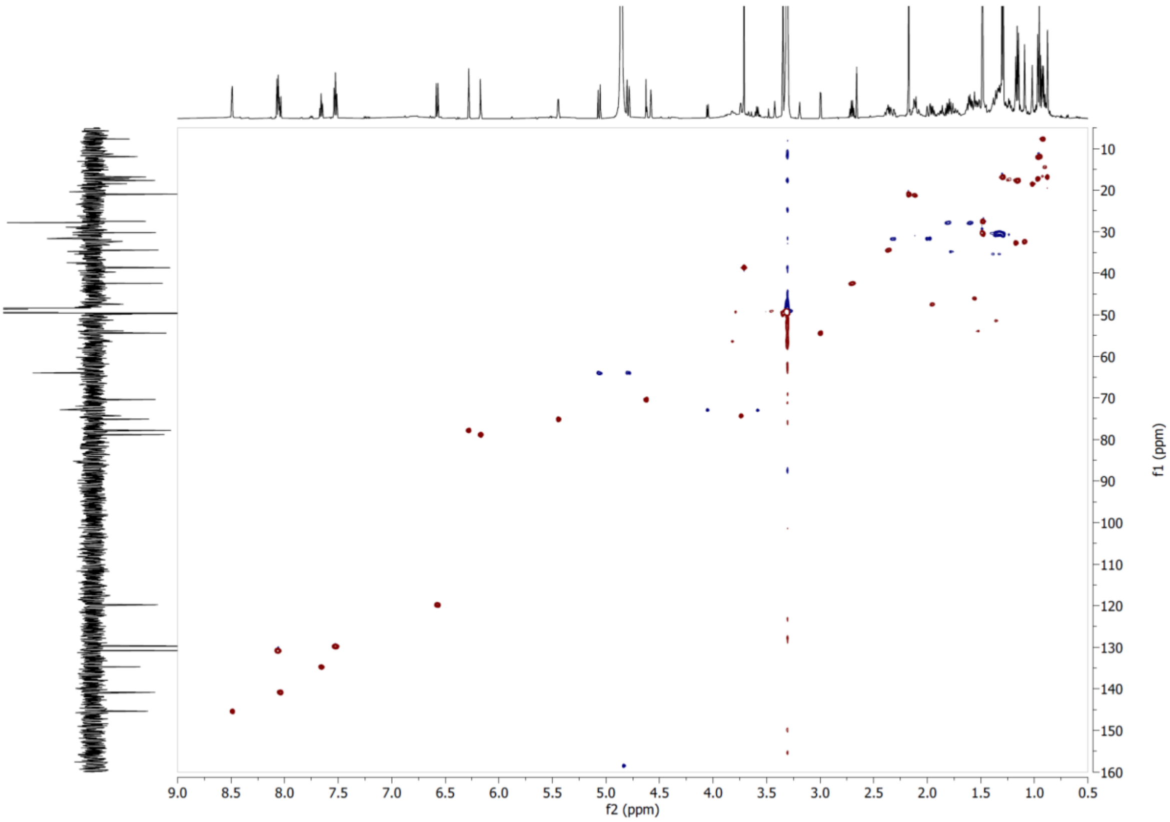

Supplementary Figure S21. HSQC NMR spectrum of compound **4** in CD_3OD.

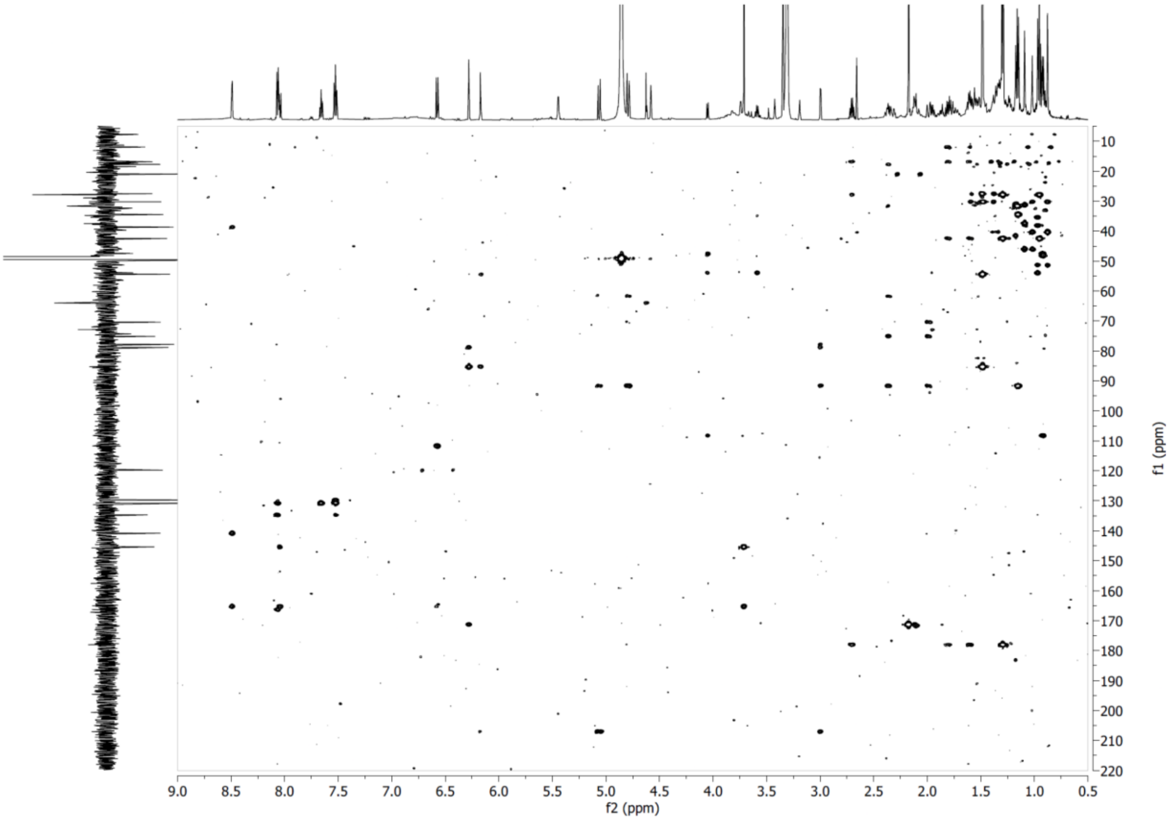

Supplementary Figure S22. HMBC NMR spectrum of compound **4** in CD_3OD.

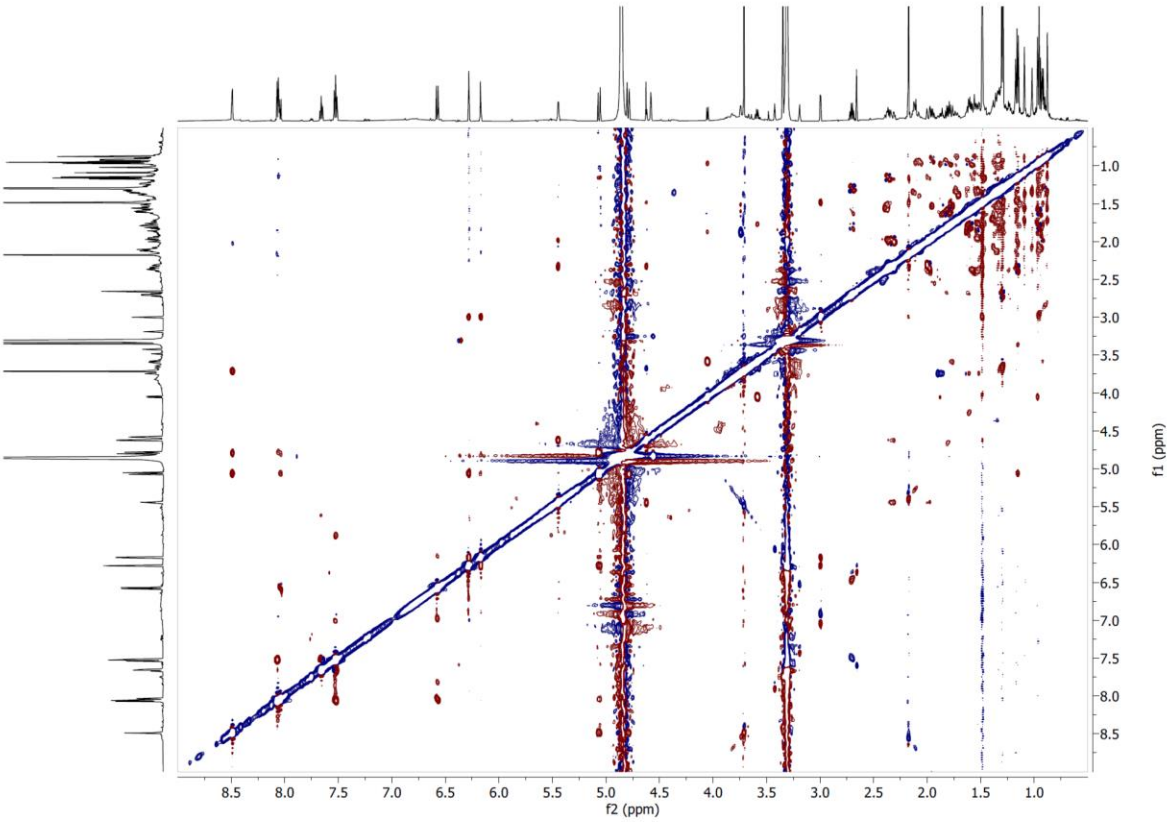

Supplementary Figure S23. ROESY NMR spectrum of compound **4** in CD_3OD at 600 MHz.

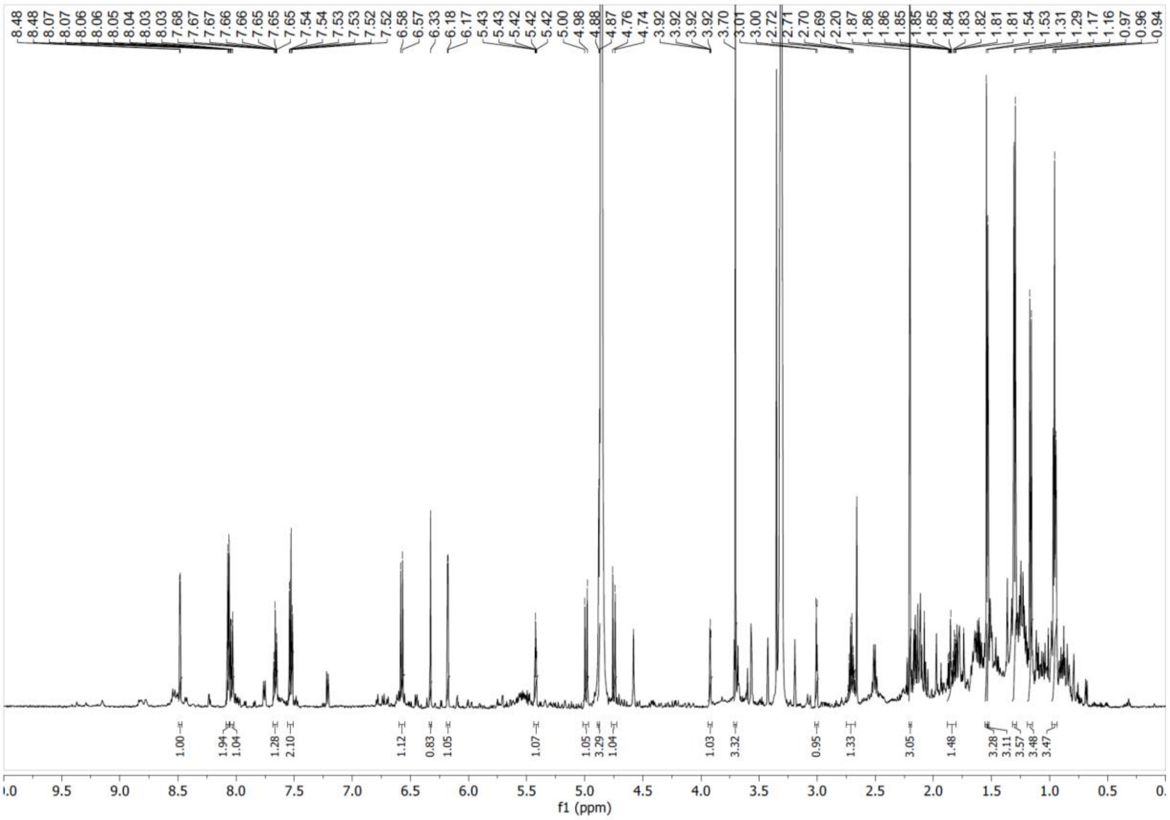

Supplementary Figure S24. ^1H NMR spectrum of compound **5** in CD_3OD at 600 MHz.

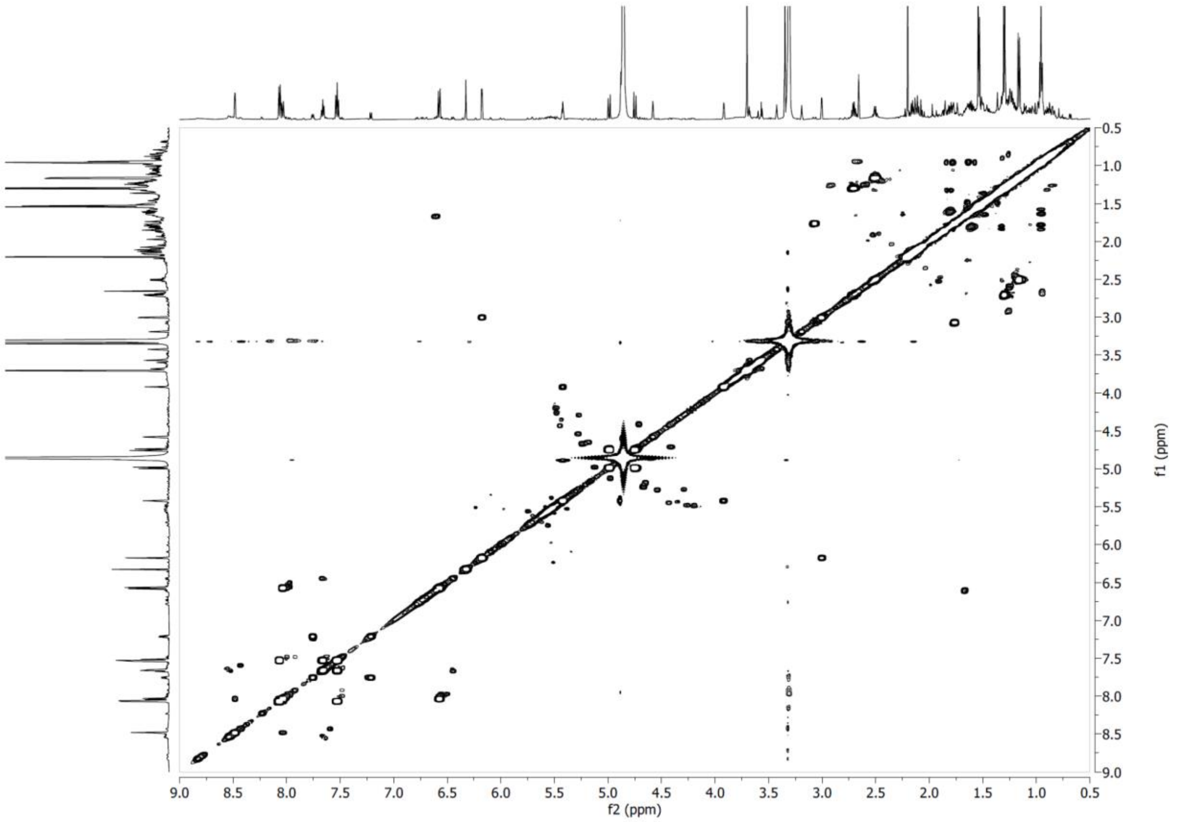

Supplementary Figure S25. COSY NMR spectrum of compound **5** in CD_3OD at 600 MHz.

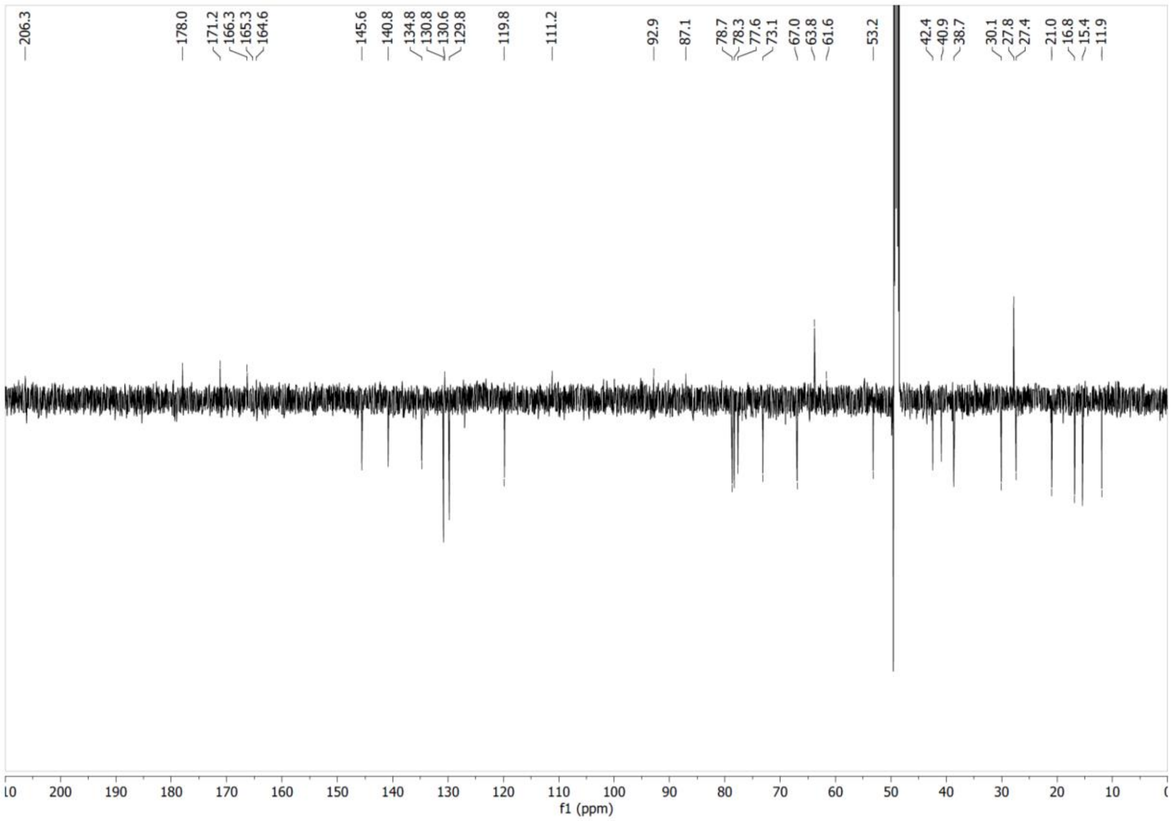

Supplementary Figure S26. ^{13}C NMR spectrum of compound **5** in CD_3OD at 151 MHz.

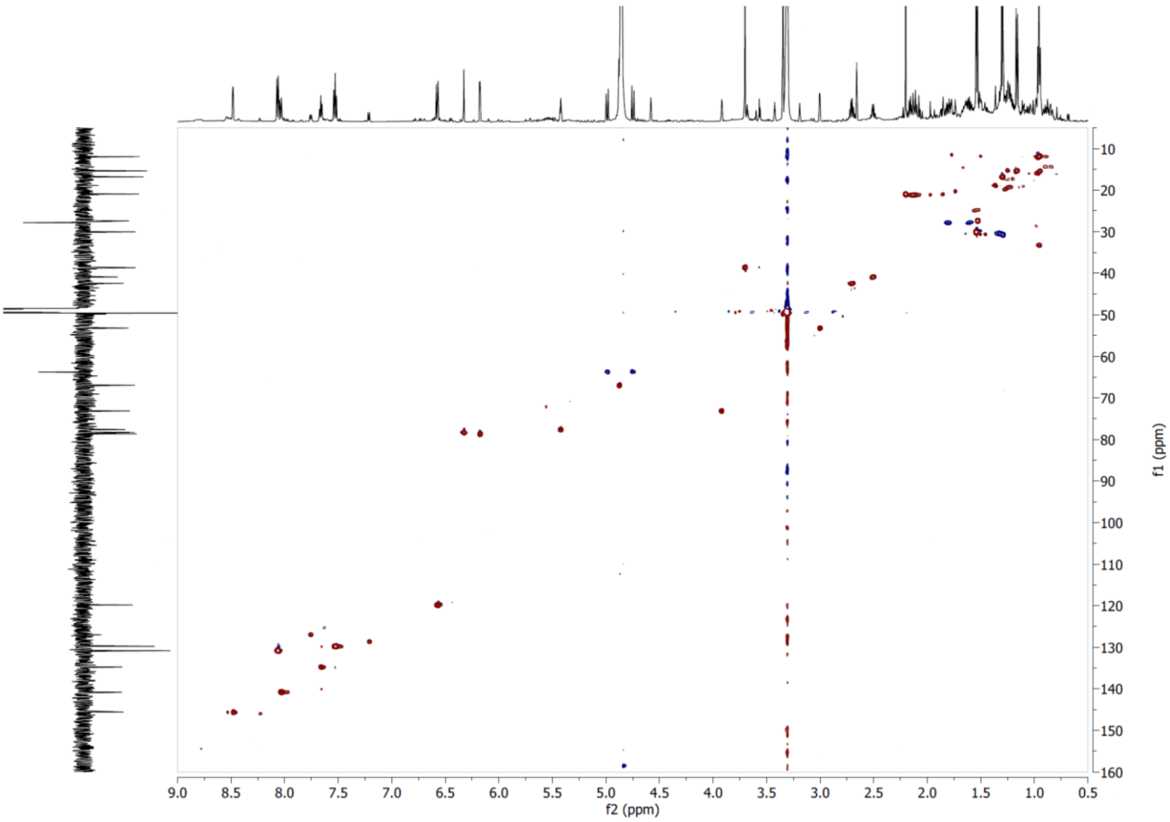

Supplementary Figure S27. HSQC NMR spectrum of compound **5** in CD_3OD.

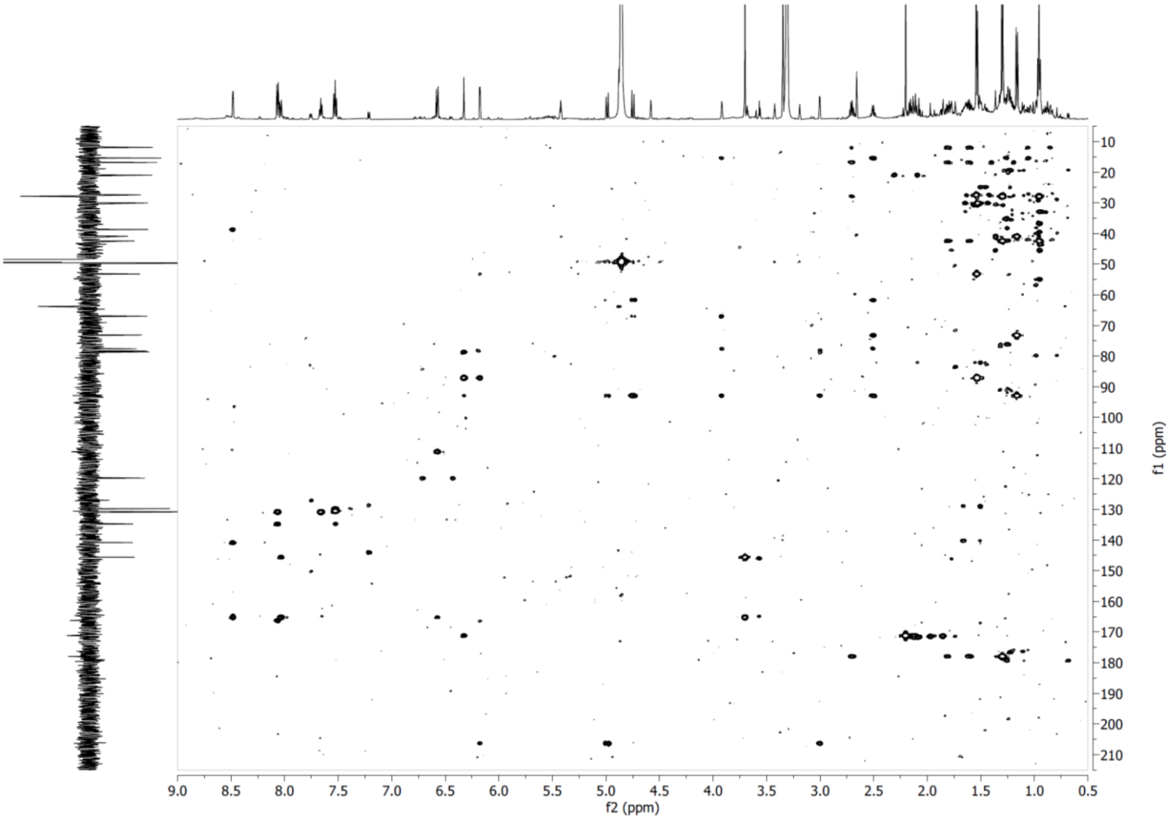

Supplementary Figure S28. HMBC NMR spectrum of compound **5** in CD_3OD.

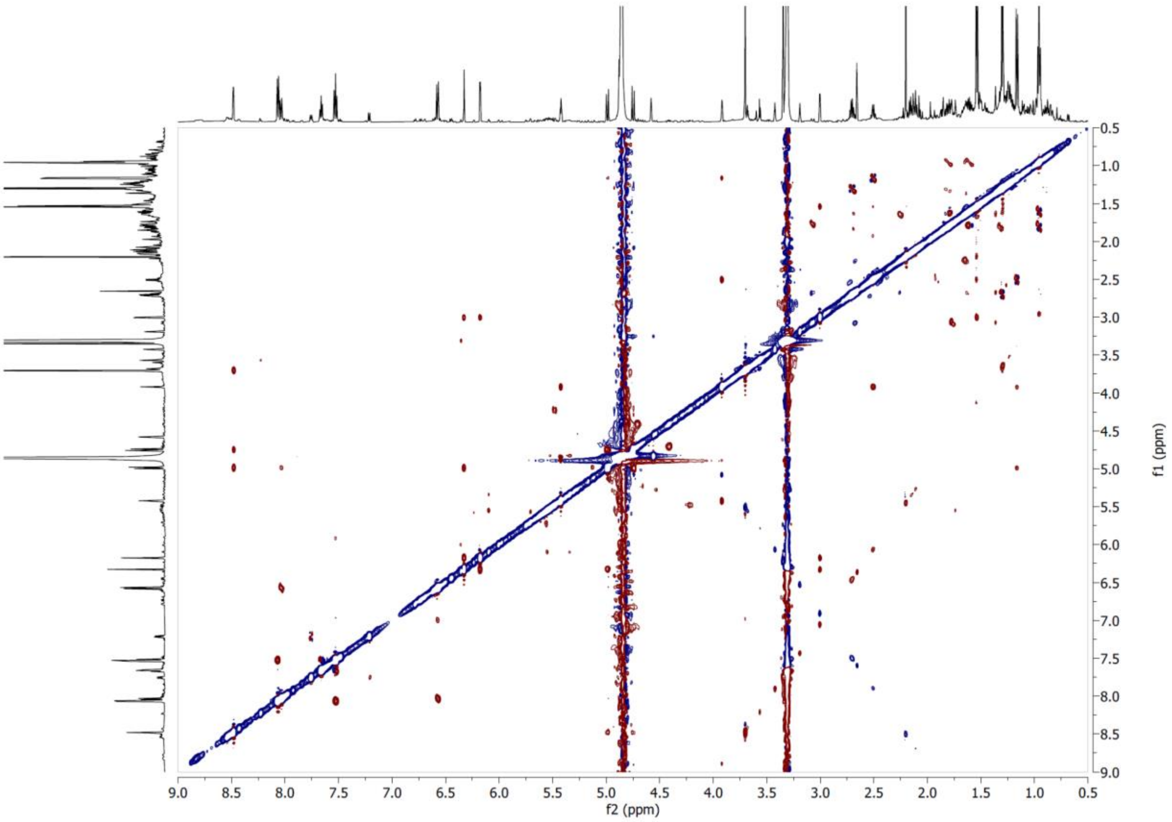

Supplementary Figure S29. ROESY NMR spectrum of compound **5** in CD_3OD at 600 MHz.

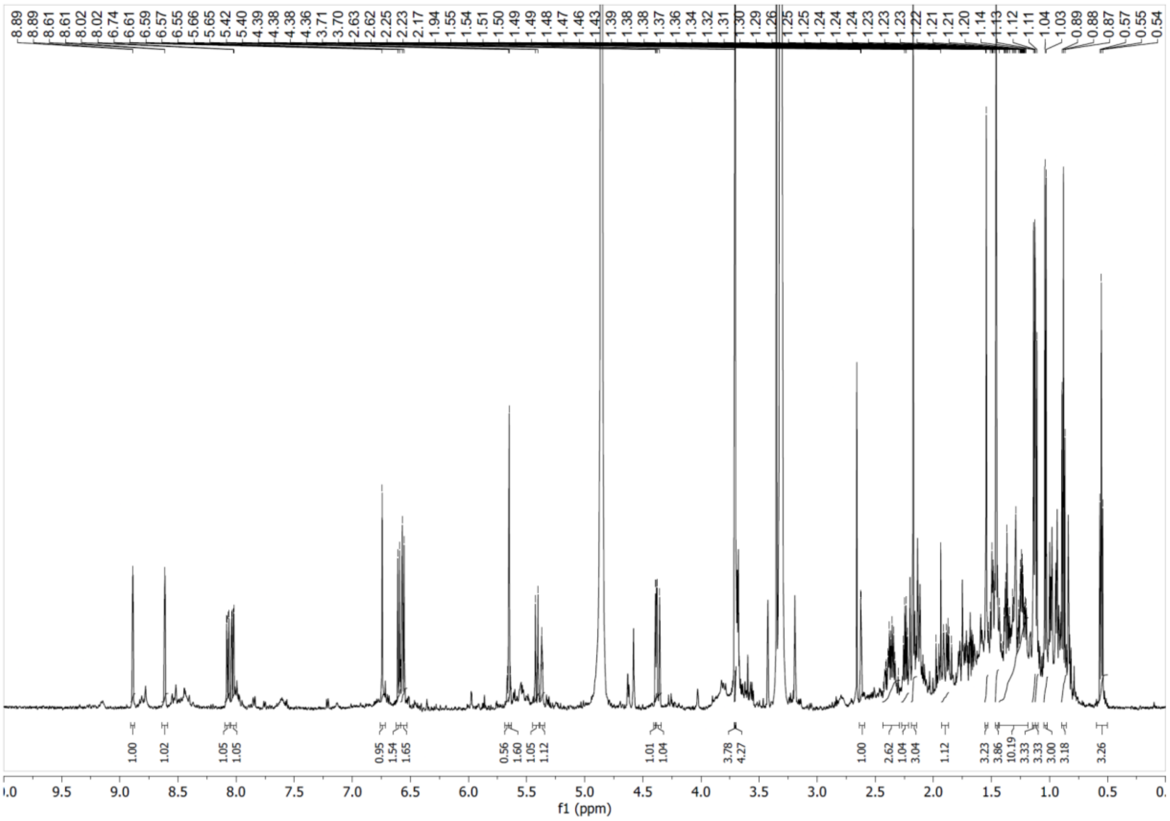

Supplementary Figure S30. ^1H NMR spectrum of compound **6** in CD_3OD at 600 MHz.

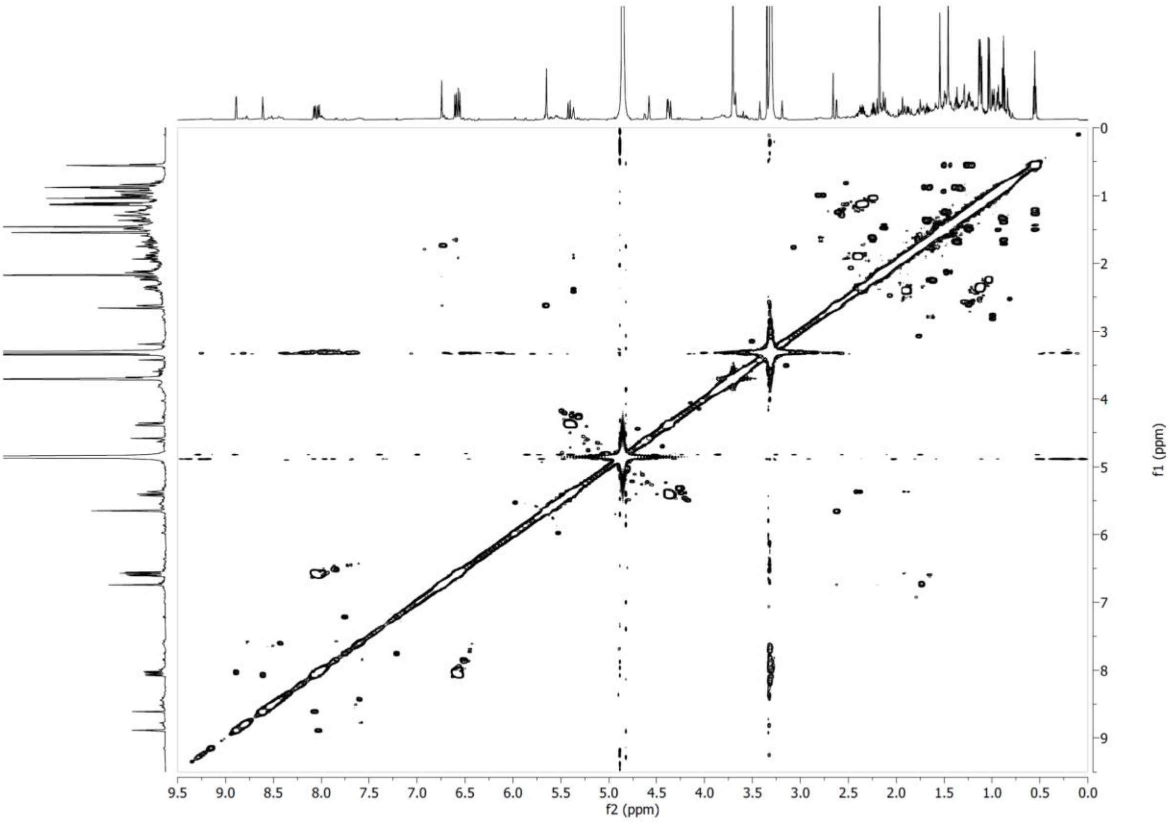

Supplementary Figure S31. COSY NMR spectrum of compound **6** in CD_3OD at 600 MHz.

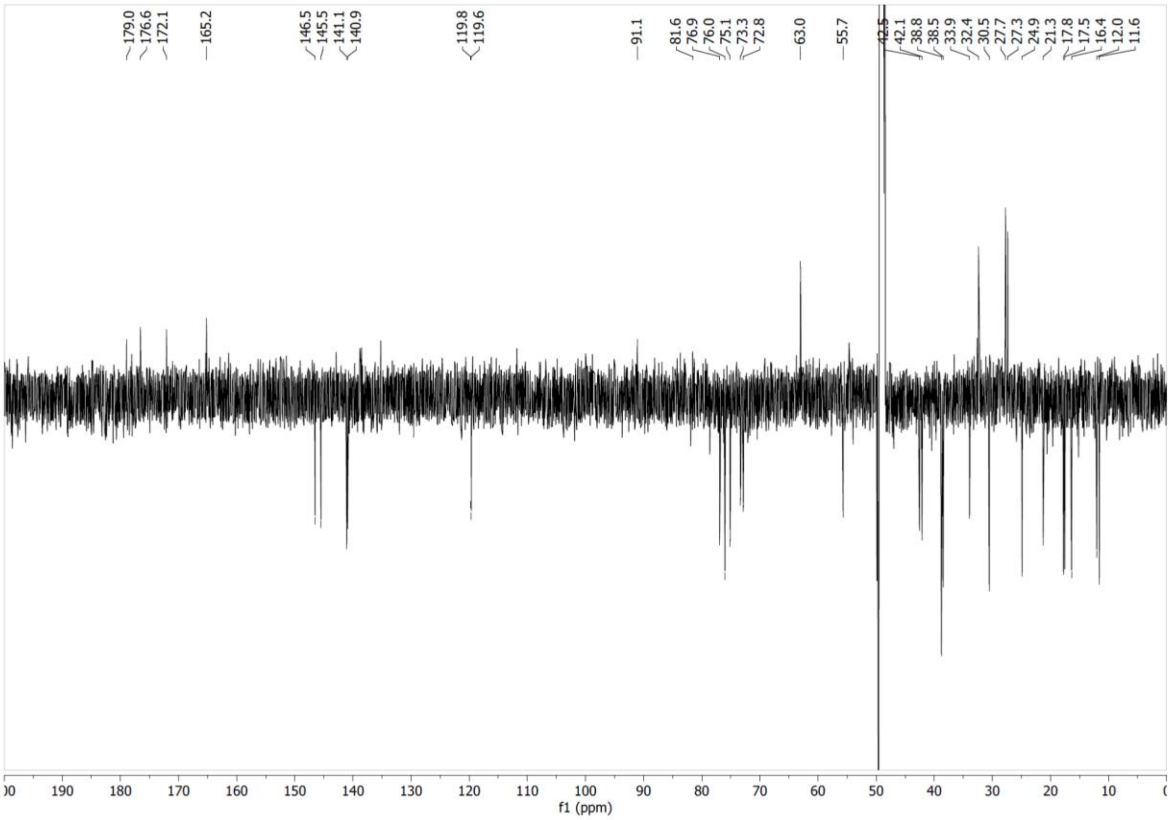

Supplementary Figure S32. ^{13}C NMR spectrum of compound **6** in CD_3OD at 151 MHz.

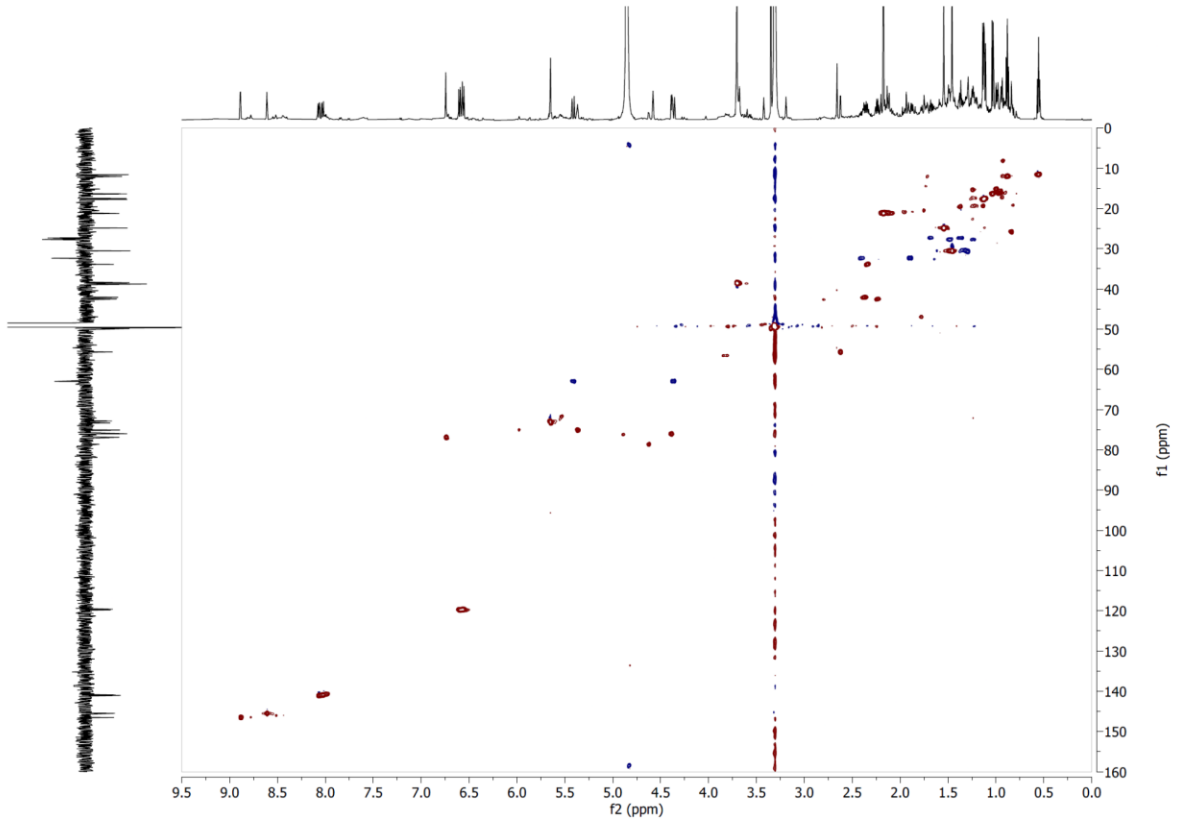

Supplementary Figure S33. HSQC NMR spectrum of compound **6** in CD_3OD.

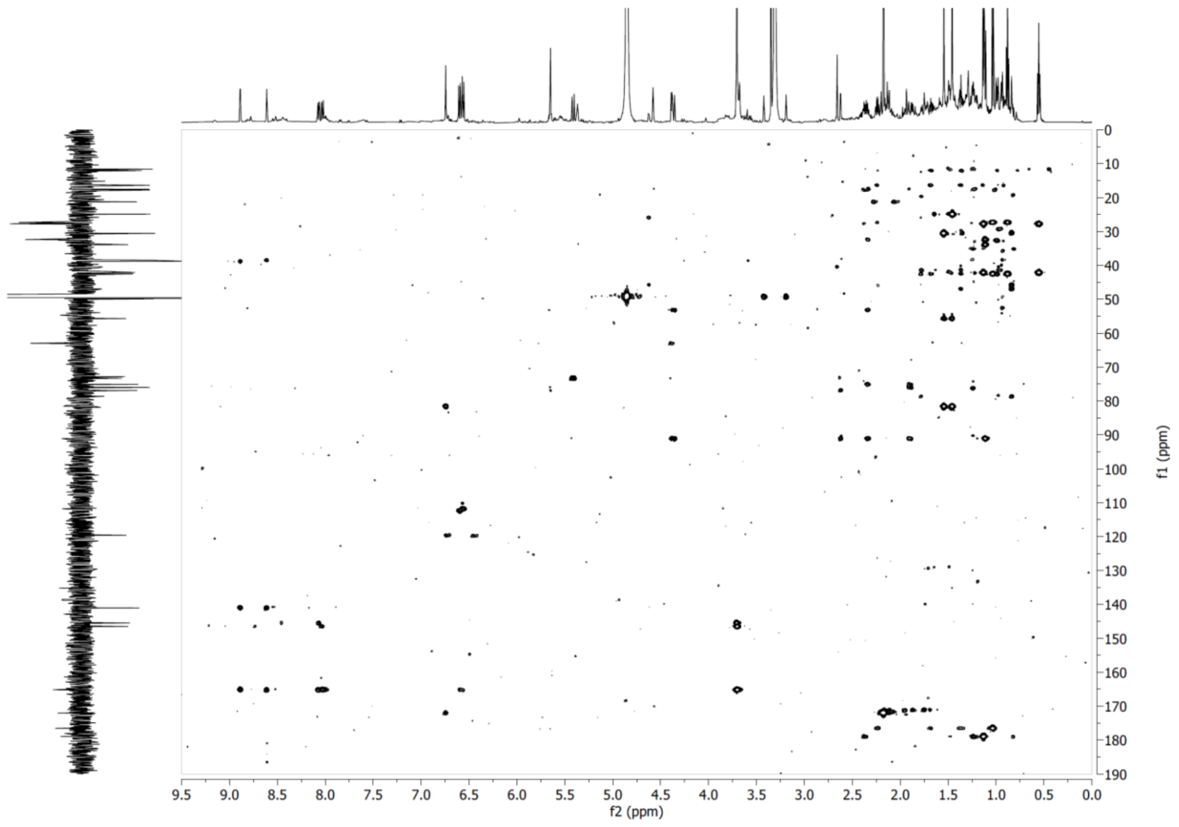

Supplementary Figure S34. HMBC NMR spectrum of compound **6** in CD_3OD.

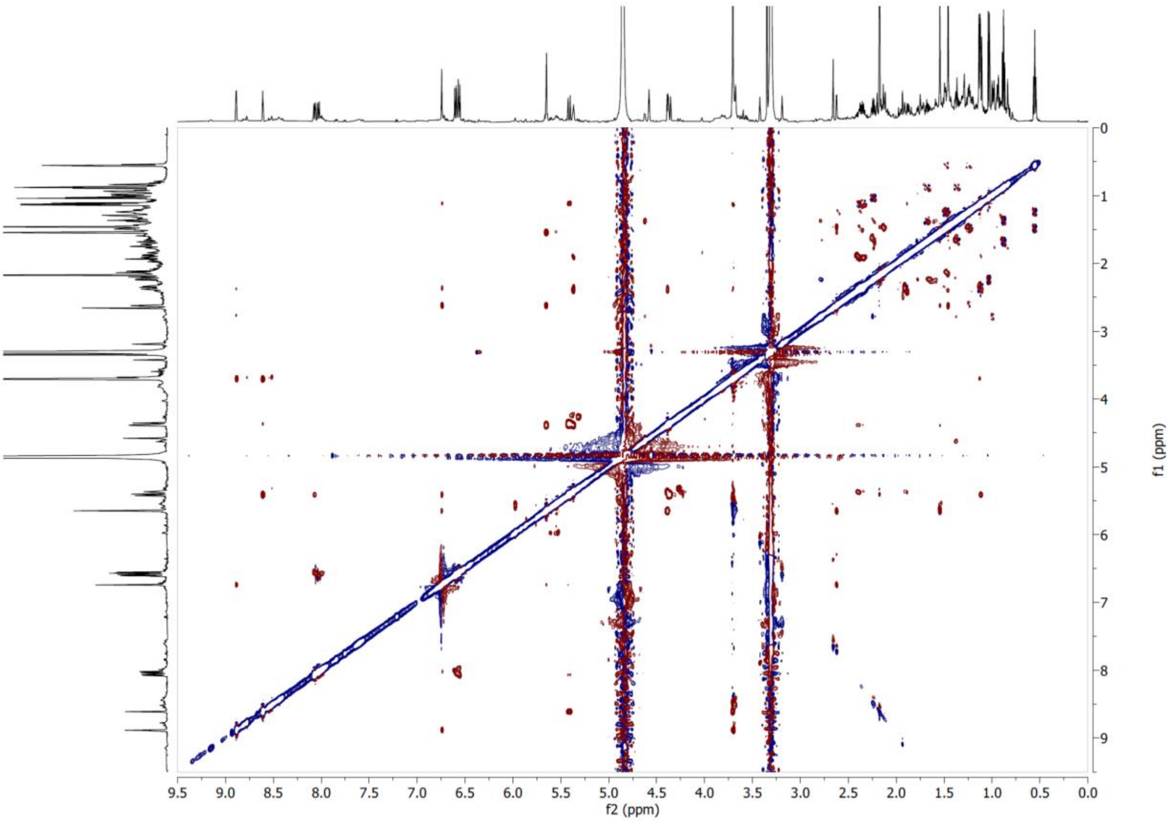

Supplementary Figure S35. ROESY NMR spectrum of compound **6** in CD_3OD at 600 MHz.

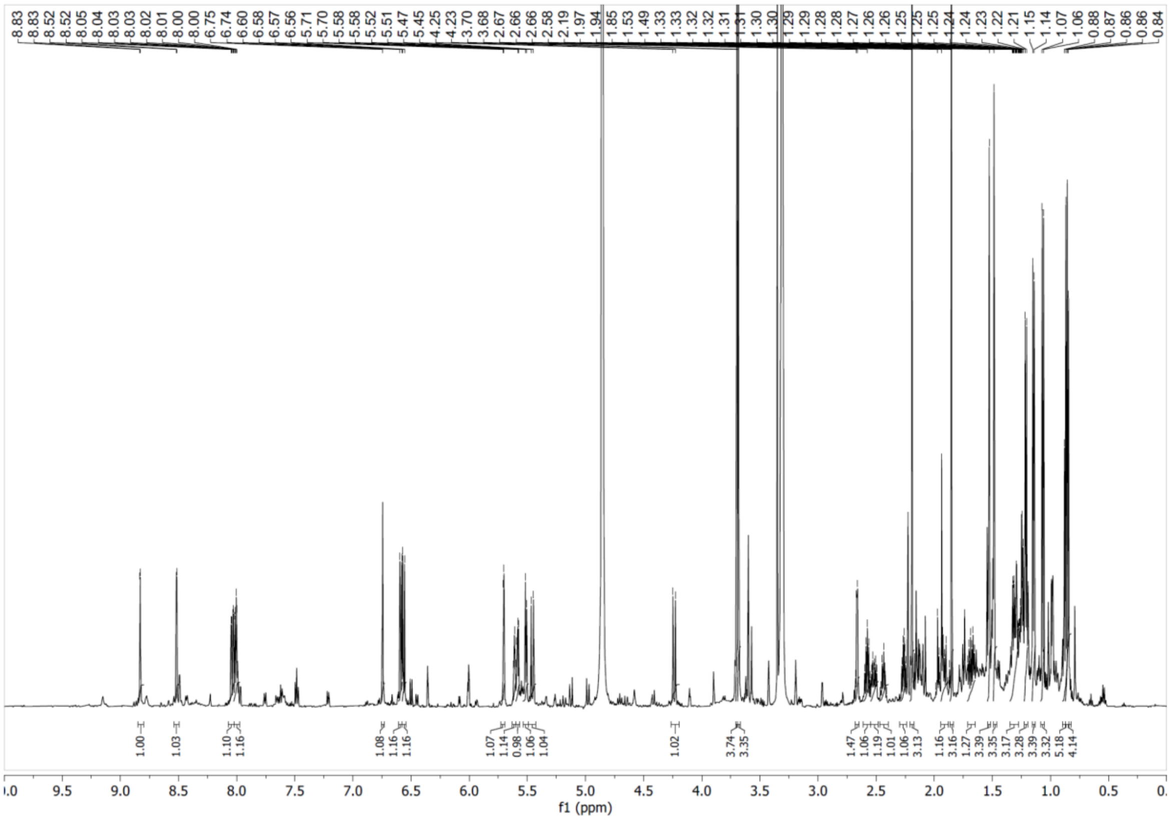

Supplementary Figure S36. ^1H NMR spectrum of compound **7** in CD_3OD at 600 MHz.

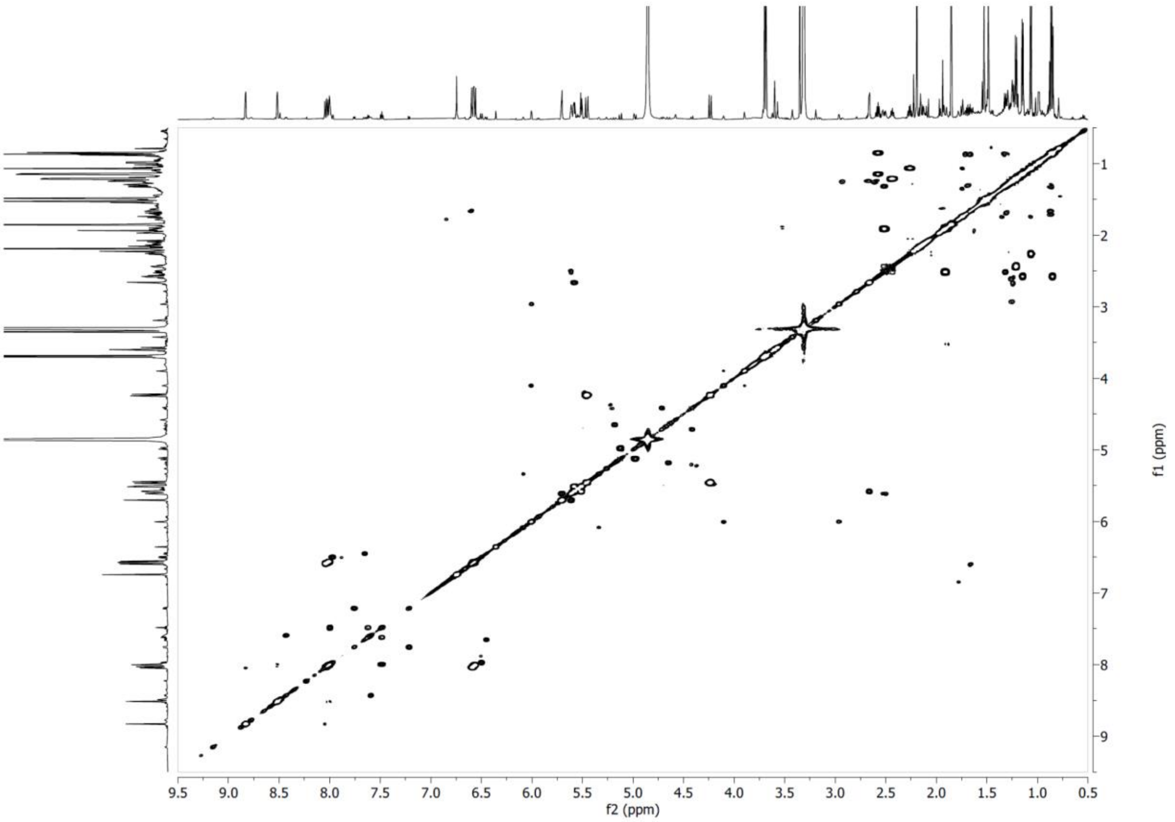

Supplementary Figure S37. COSY NMR spectrum of compound **7** in CD_3OD at 600 MHz.

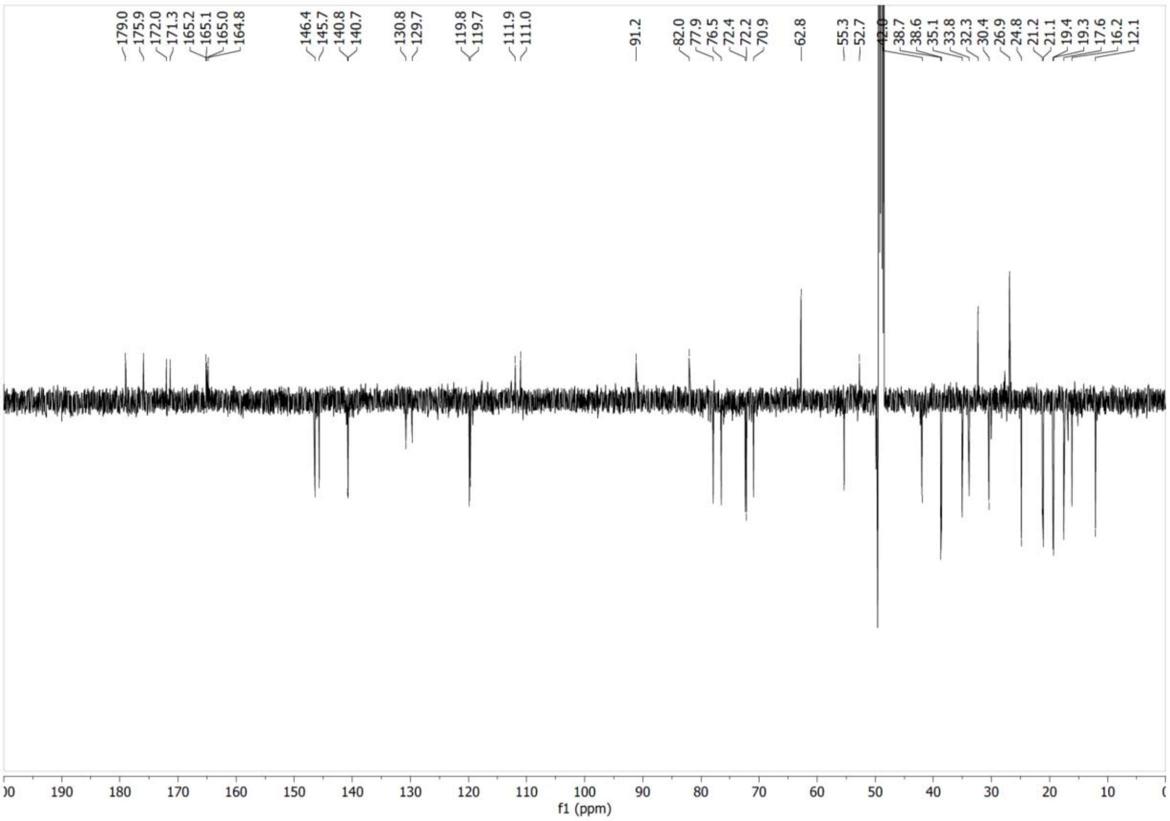

Supplementary Figure S38. ^{13}C NMR spectrum of compound **7** in CD_3OD at 151 MHz.

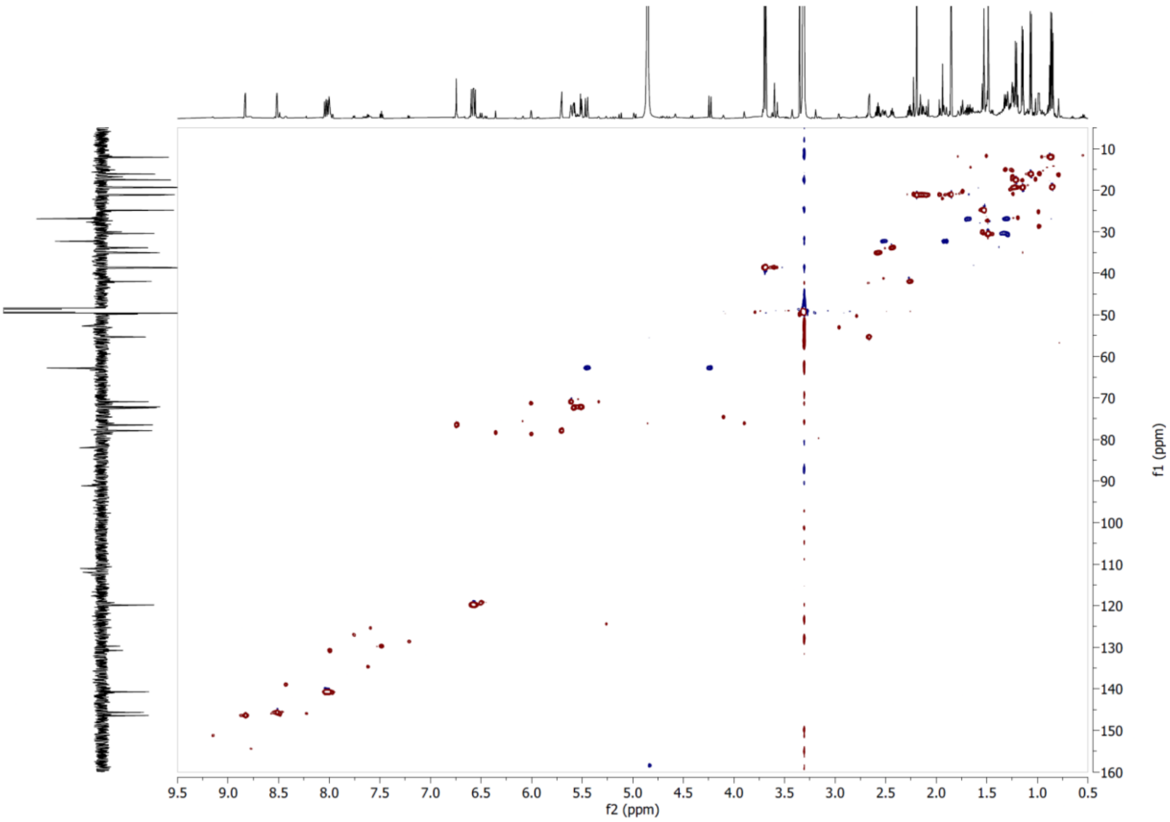

Supplementary Figure S39. HSQC NMR spectrum of compound **7** in CD_3OD.

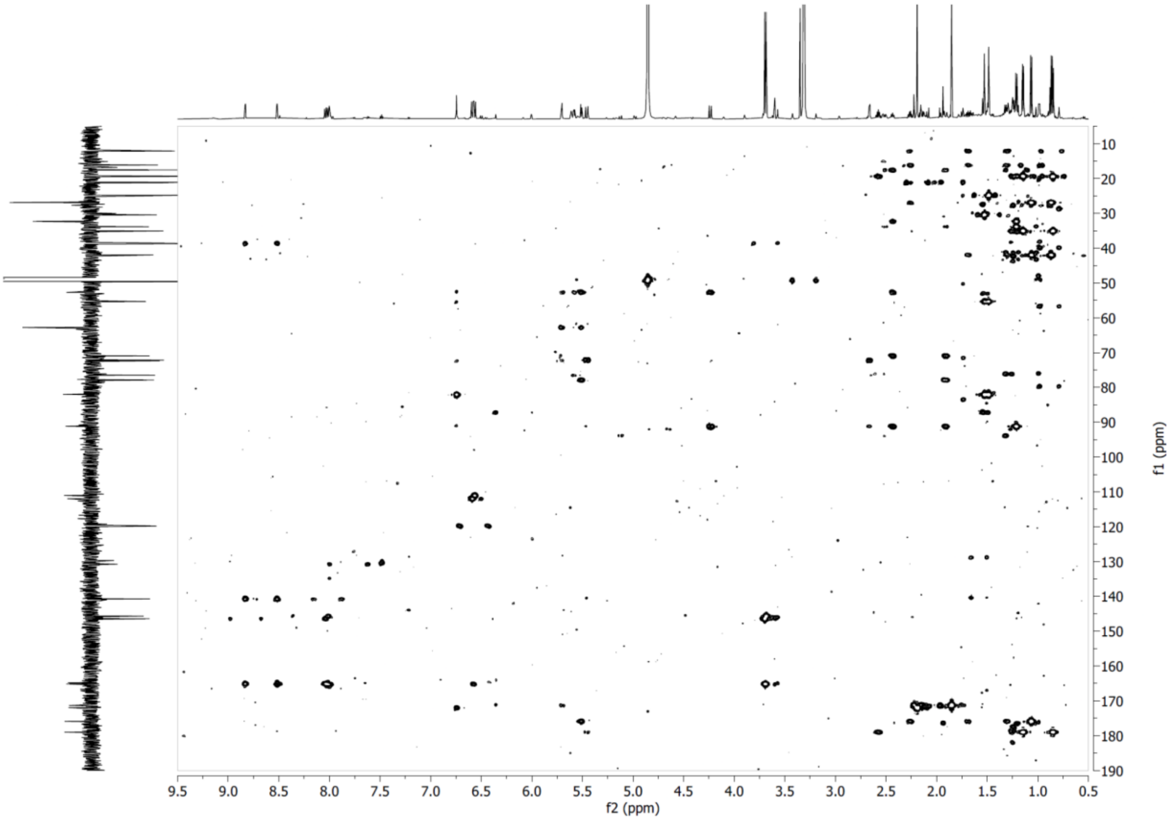

Supplementary Figure S40. HMBC NMR spectrum of compound **7** in CD_3OD.

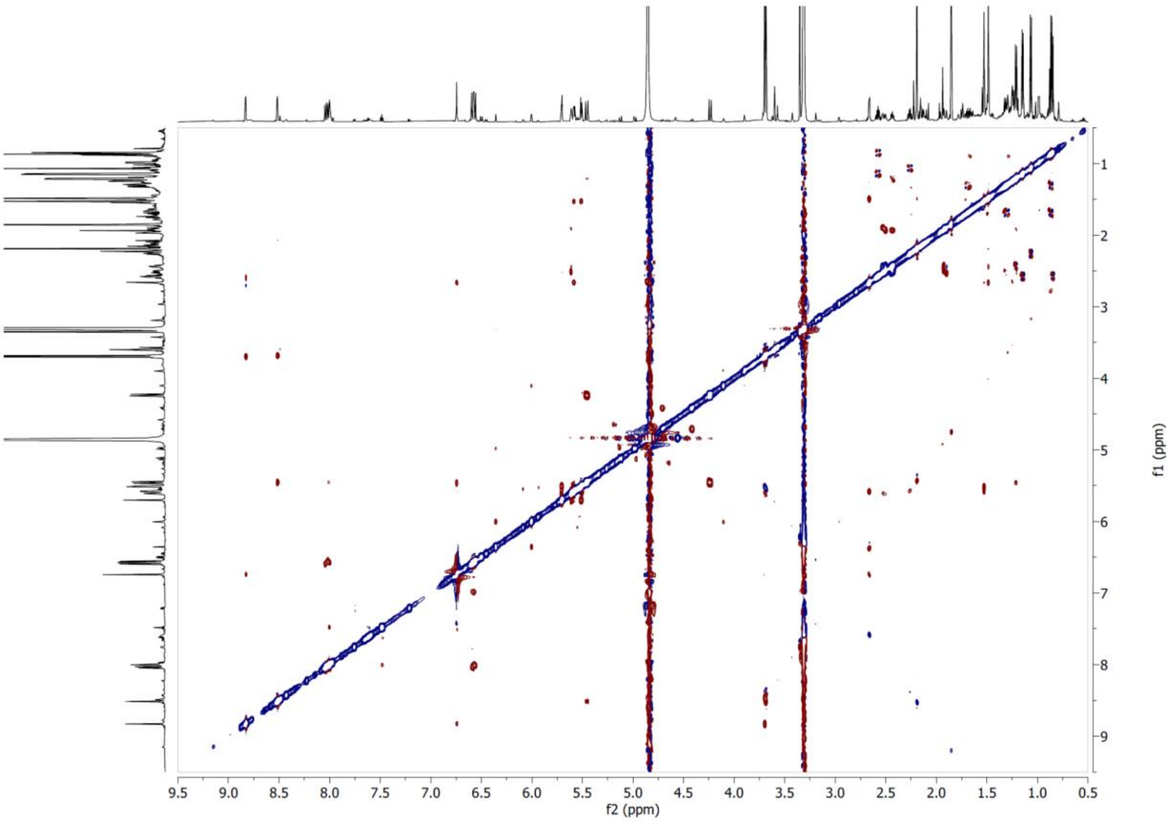

Supplementary Figure S41. ROESY NMR spectrum of compound **7** in CD_3OD at 600 MHz.

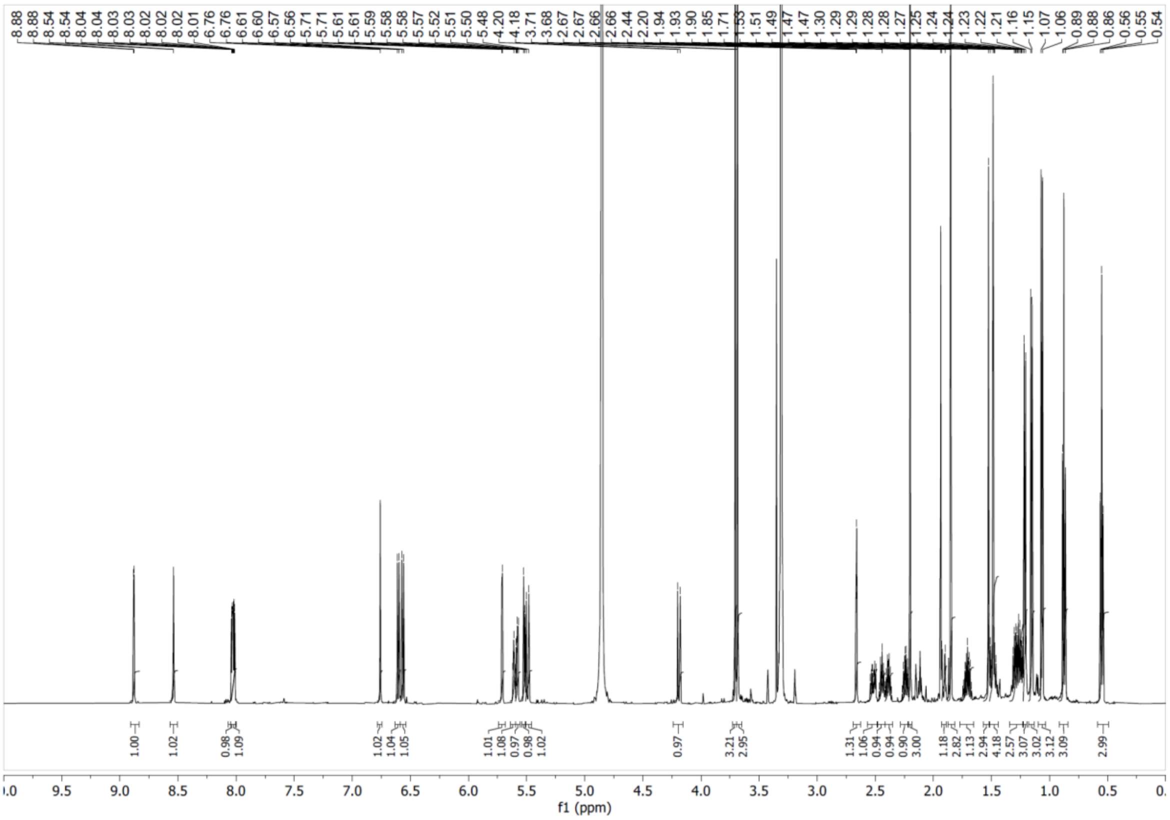

Supplementary Figure S42. ^{1}H NMR spectrum of compound **8** in CD_3OD at 600 MHz.

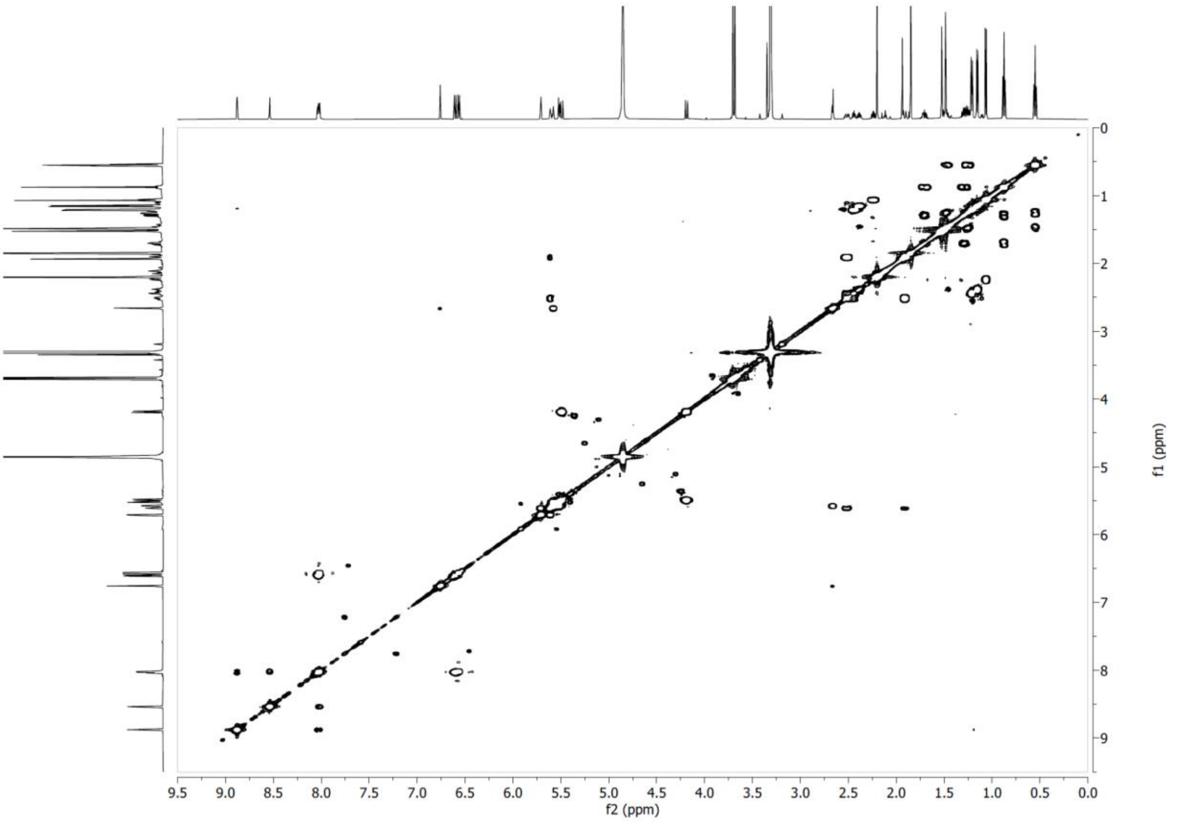

Supplementary Figure S43. COSY NMR spectrum of compound **8** in CD_3OD at 600 MHz.

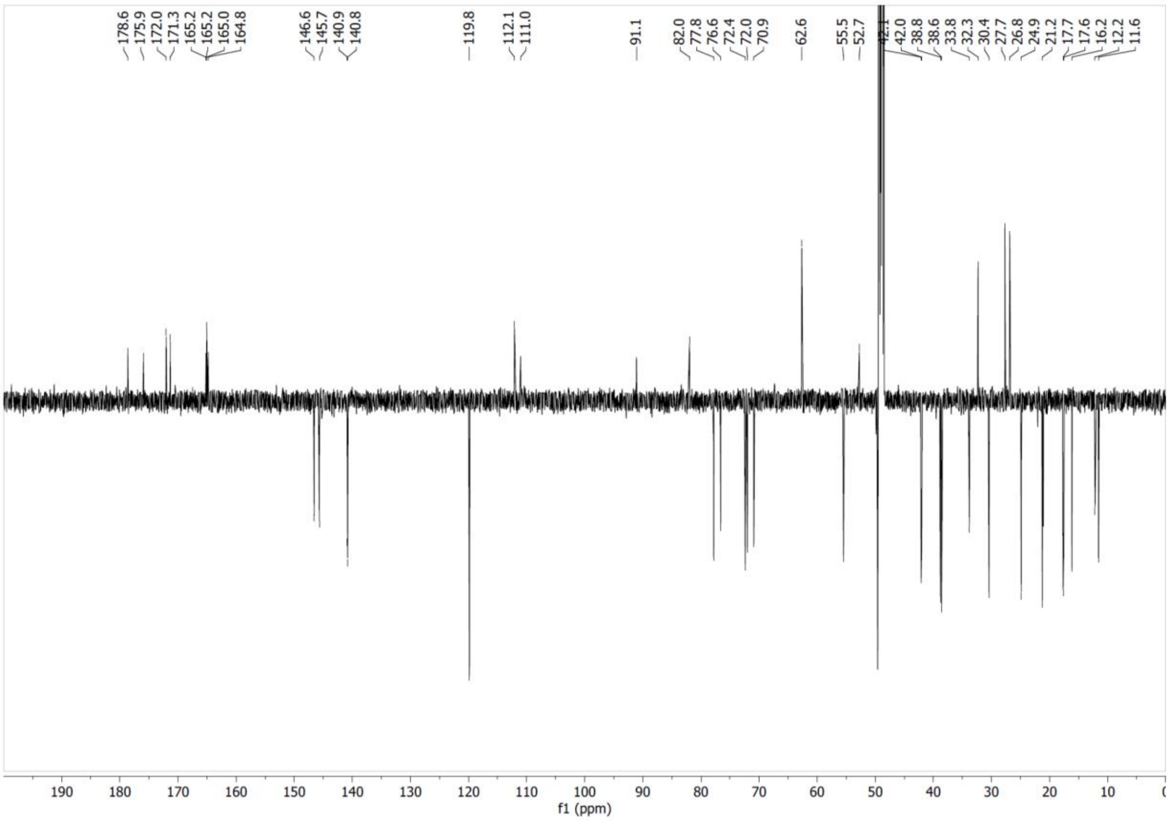

Supplementary Figure S44. ^{13}C NMR spectrum of compound **8** in CD_3OD at 151 MHz.

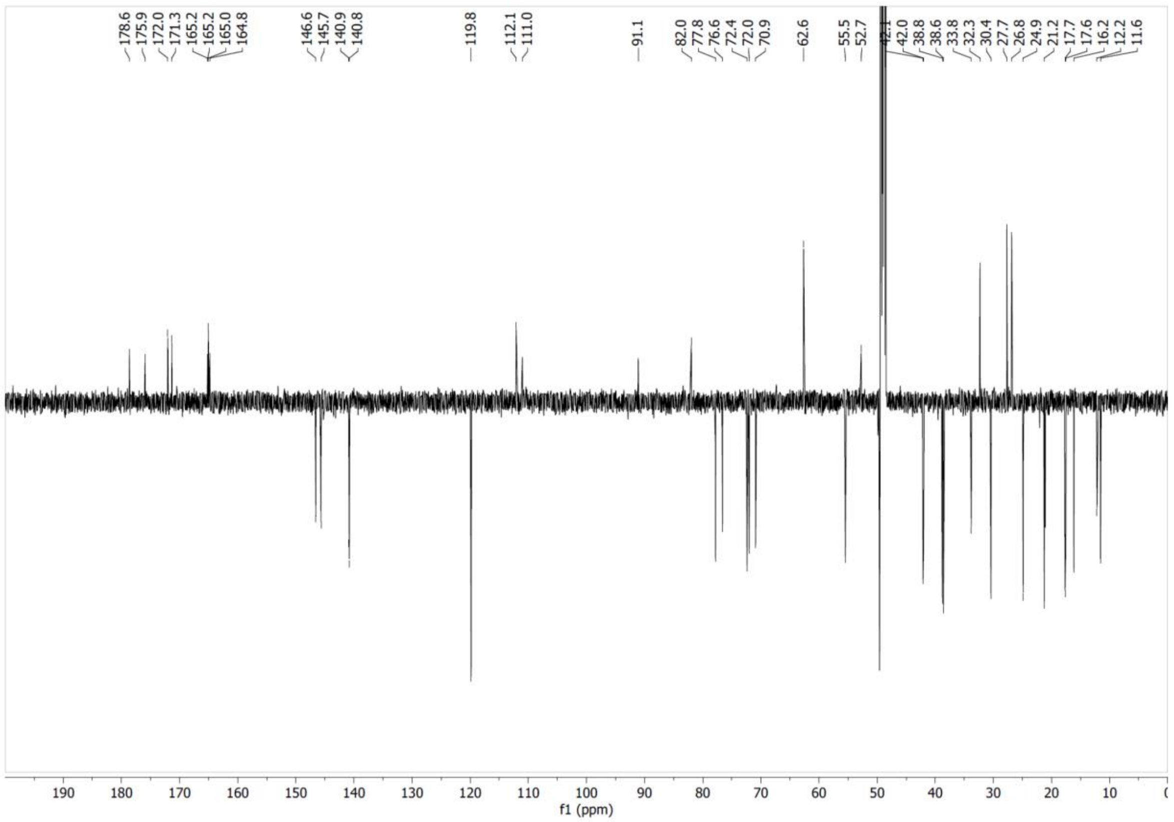

Supplementary Figure S45. HSQC NMR spectrum of compound **8** in CD_3OD.

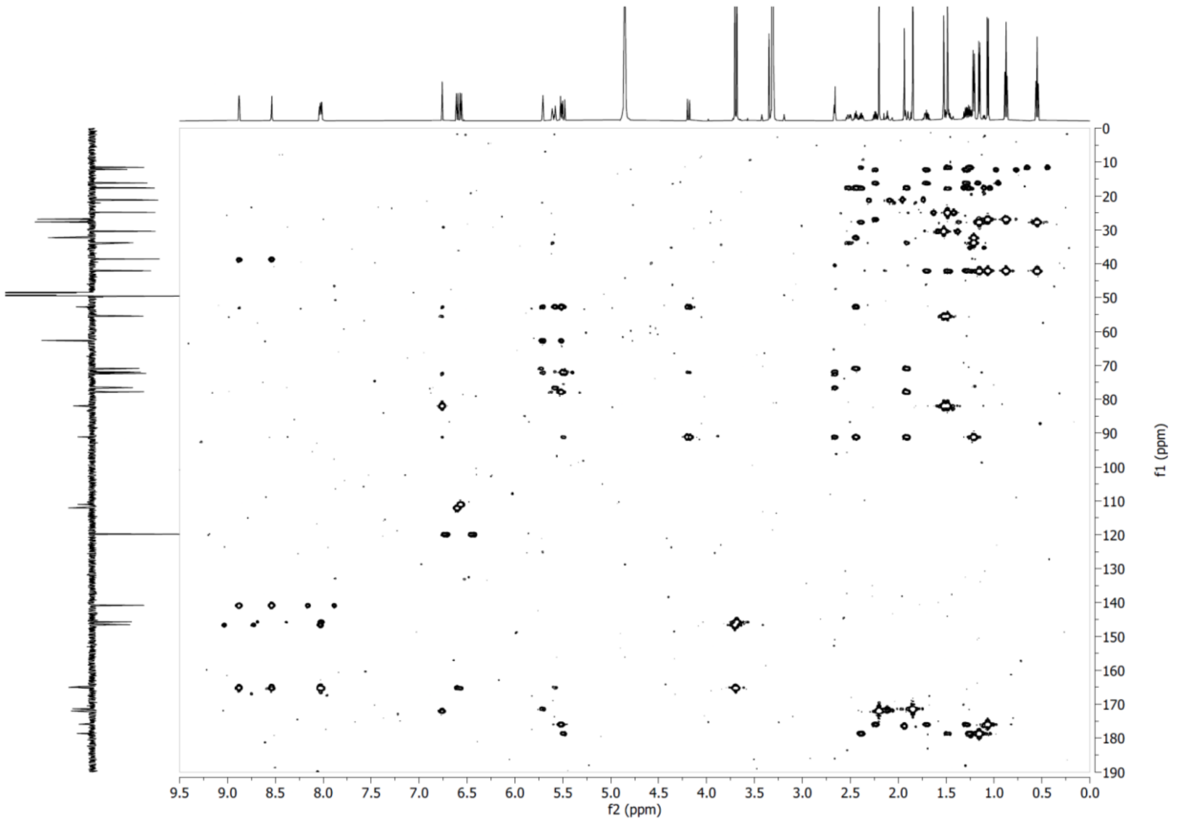

Supplementary Figure S46. HMBC NMR spectrum of compound **8** in CD_3OD.

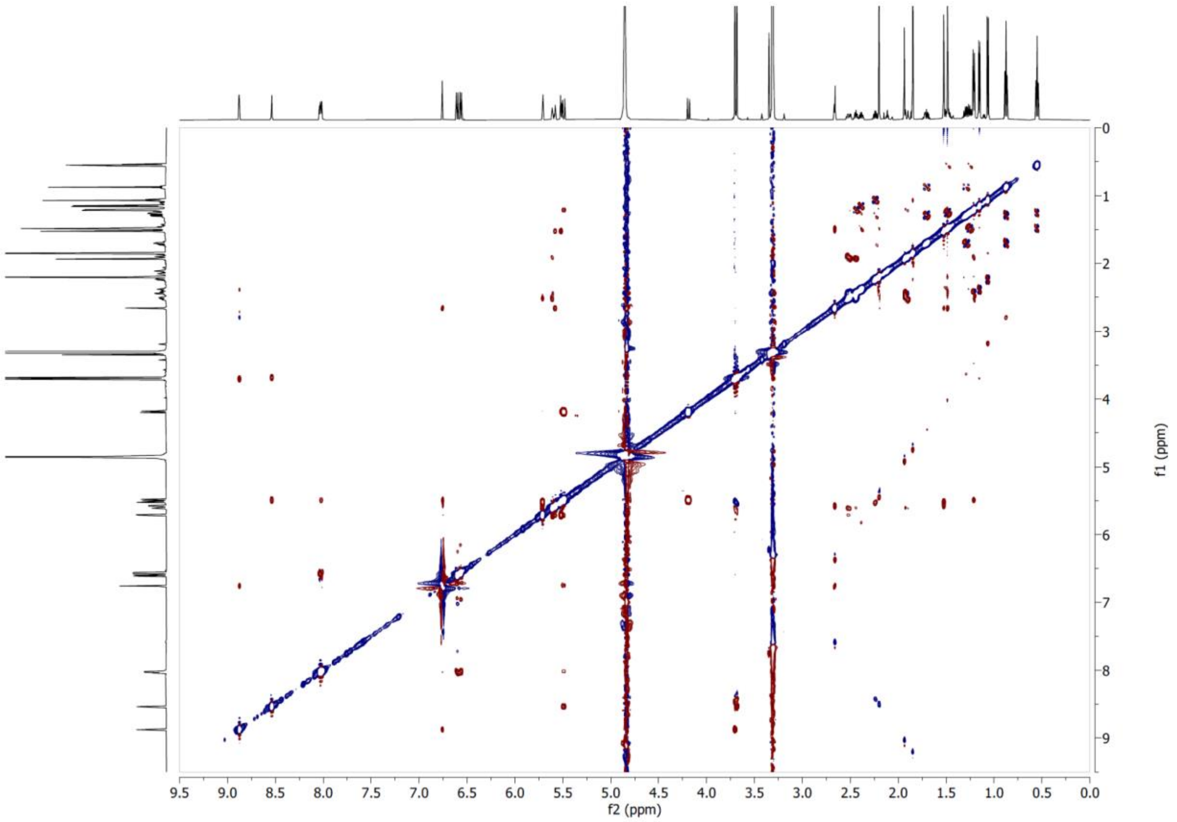

Supplementary Figure S47. ROESY NMR spectrum of compound **8** in CD_3OD at 600 MHz.

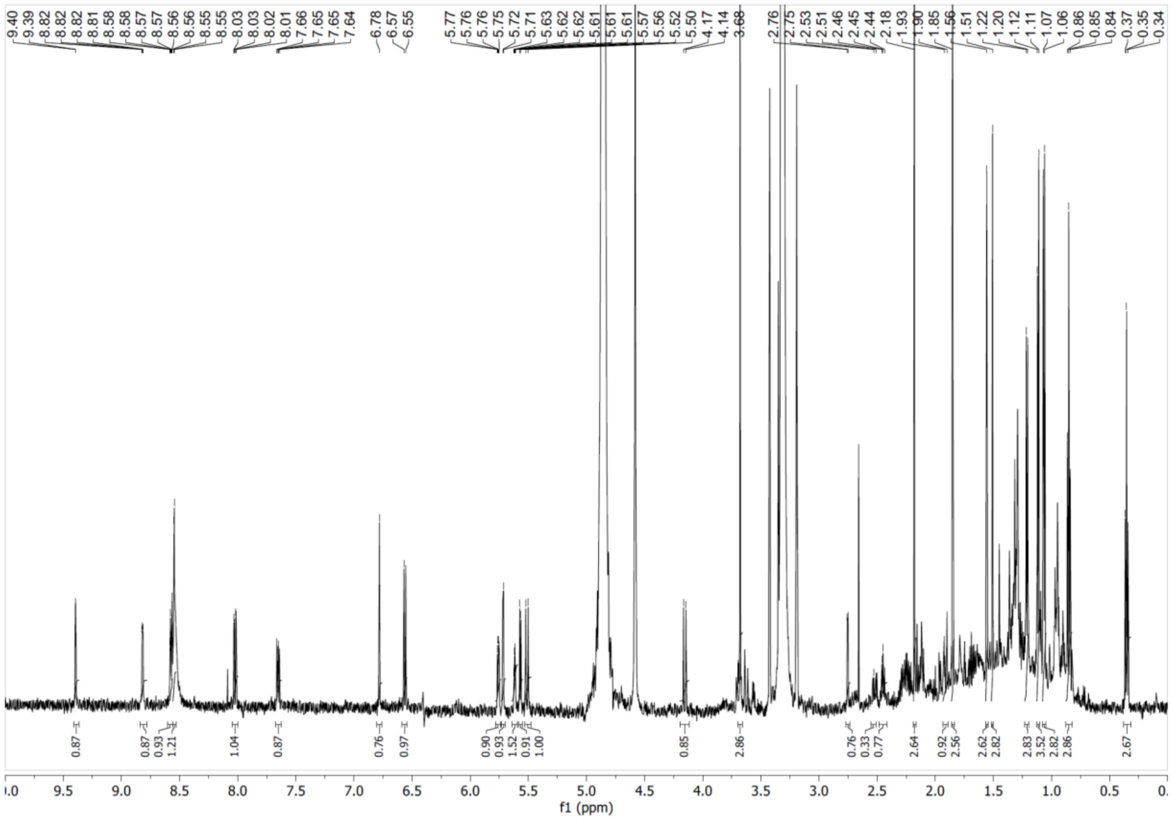

Supplementary Figure S48. ^1H NMR spectrum of compound **9** in CD_3OD at 600 MHz.

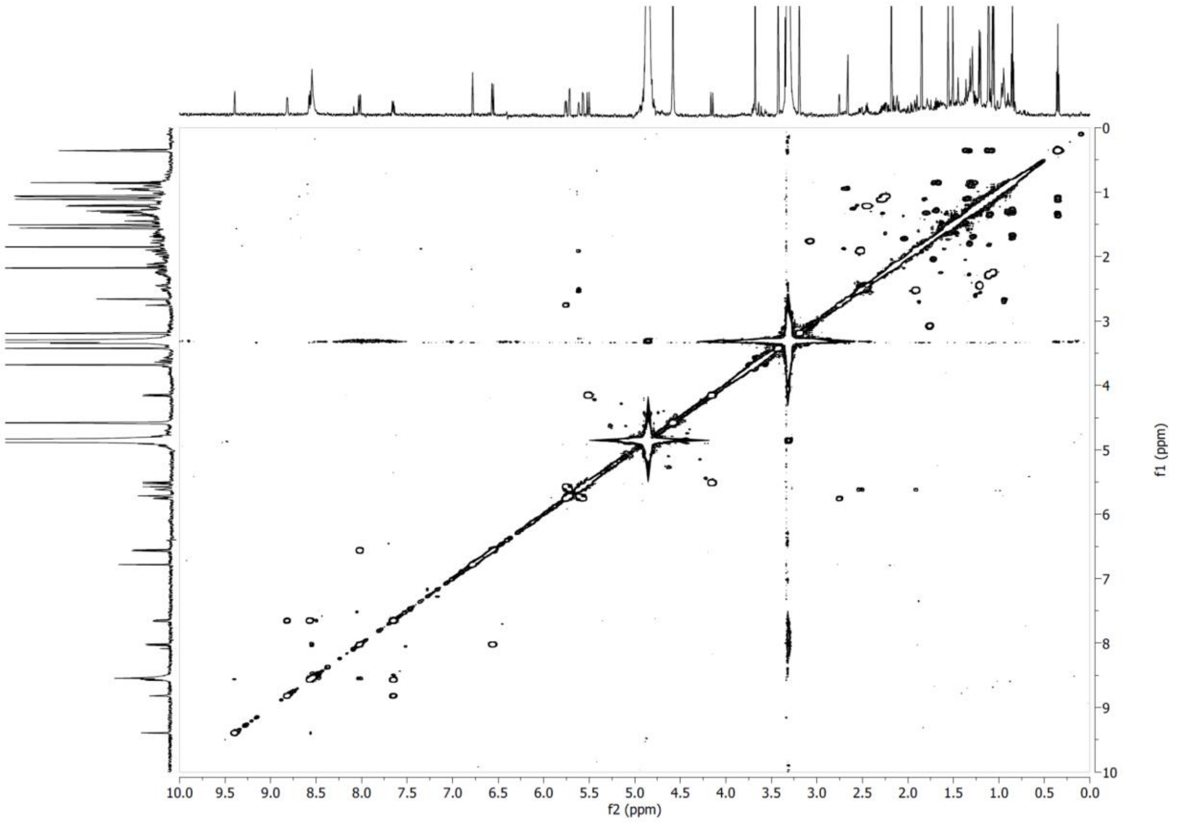

Supplementary Figure S49. COSY NMR spectrum of compound **9** in CD_3OD at 600 MHz.

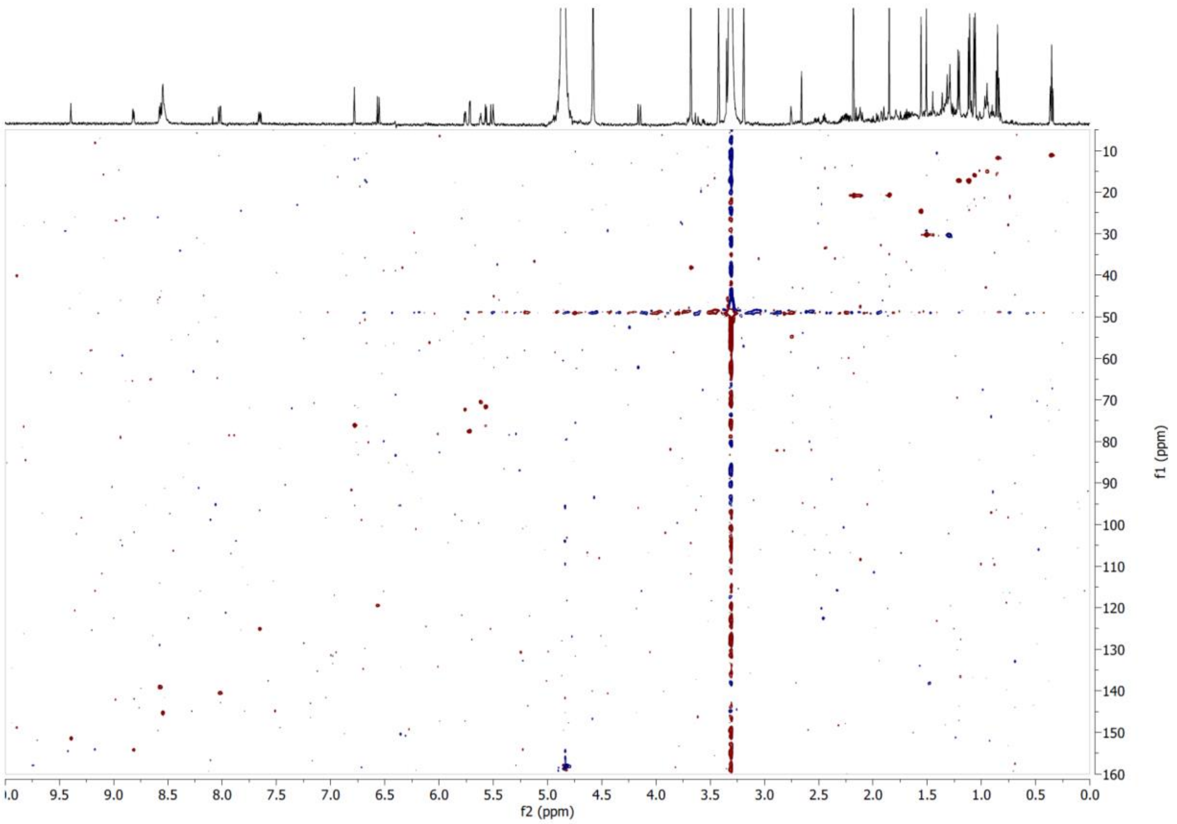

Supplementary Figure S50. HSQC NMR spectrum of compound **9** in CD_3OD.

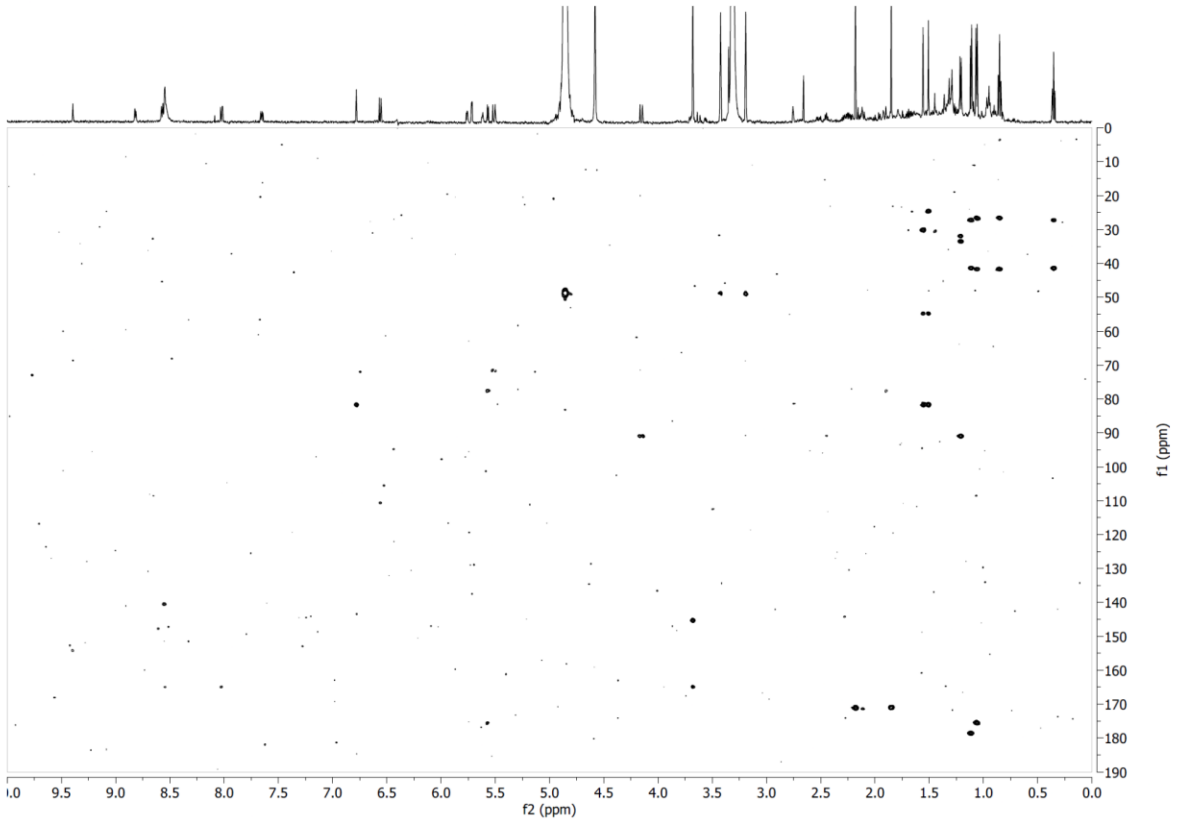

Supplementary Figure S51. HMBC NMR spectrum of compound **9** in CD_3OD.

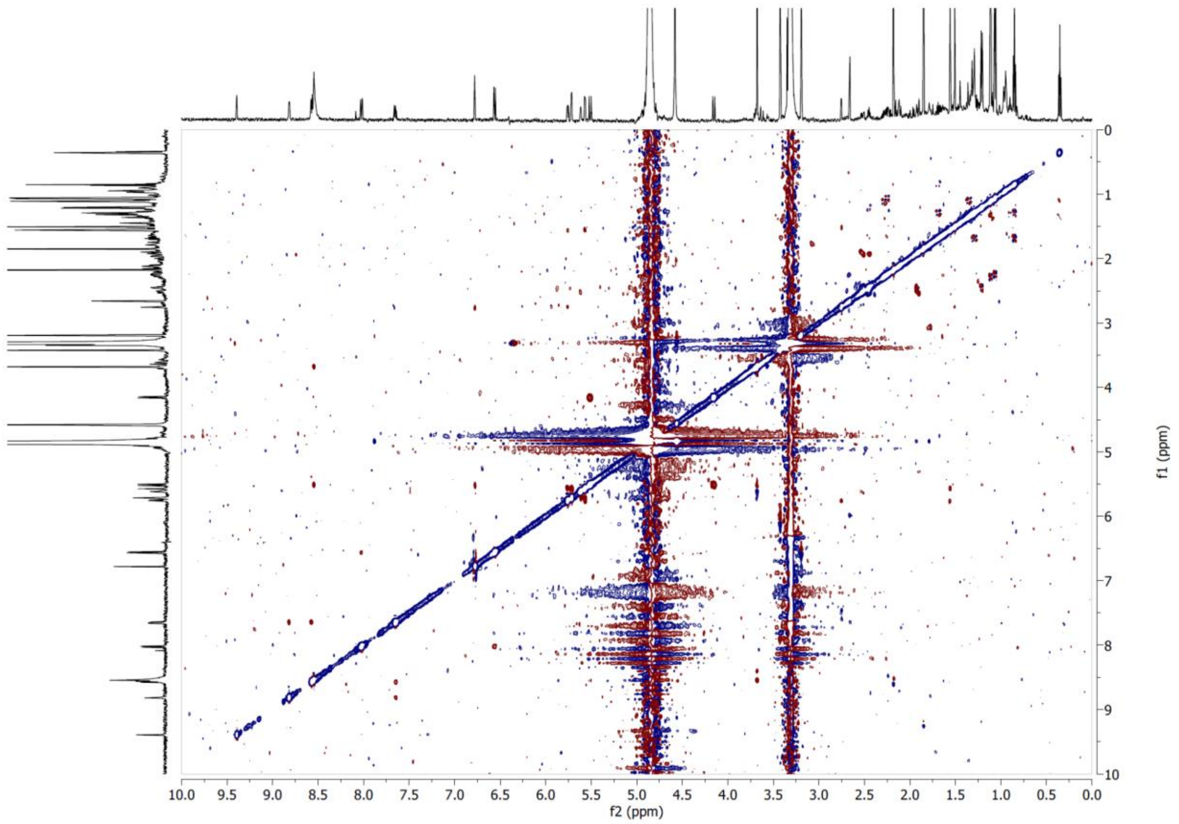

Supplementary Figure S52. ROESY NMR spectrum of compound **9** in CD_3OD at 600 MHz.

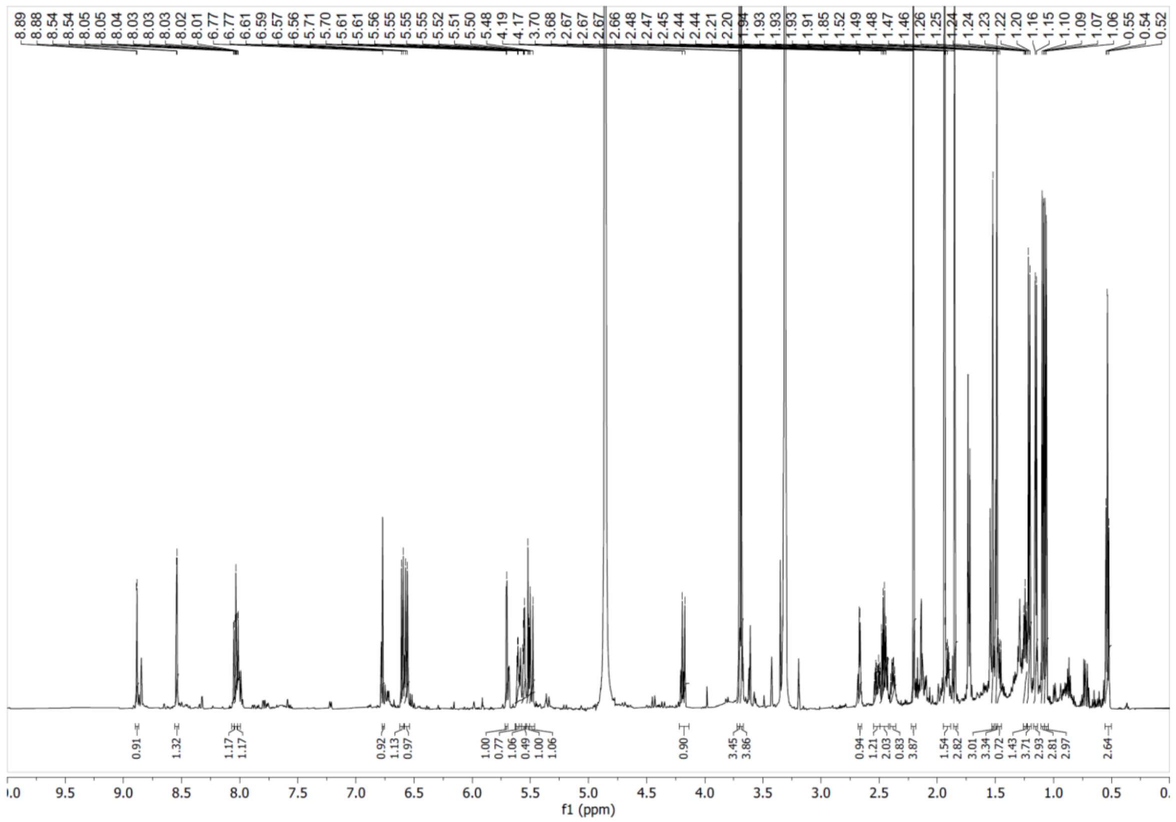

Supplementary Figure S53. ^{1}H NMR spectrum of compound **10** in CD_3OD at 600 MHz.

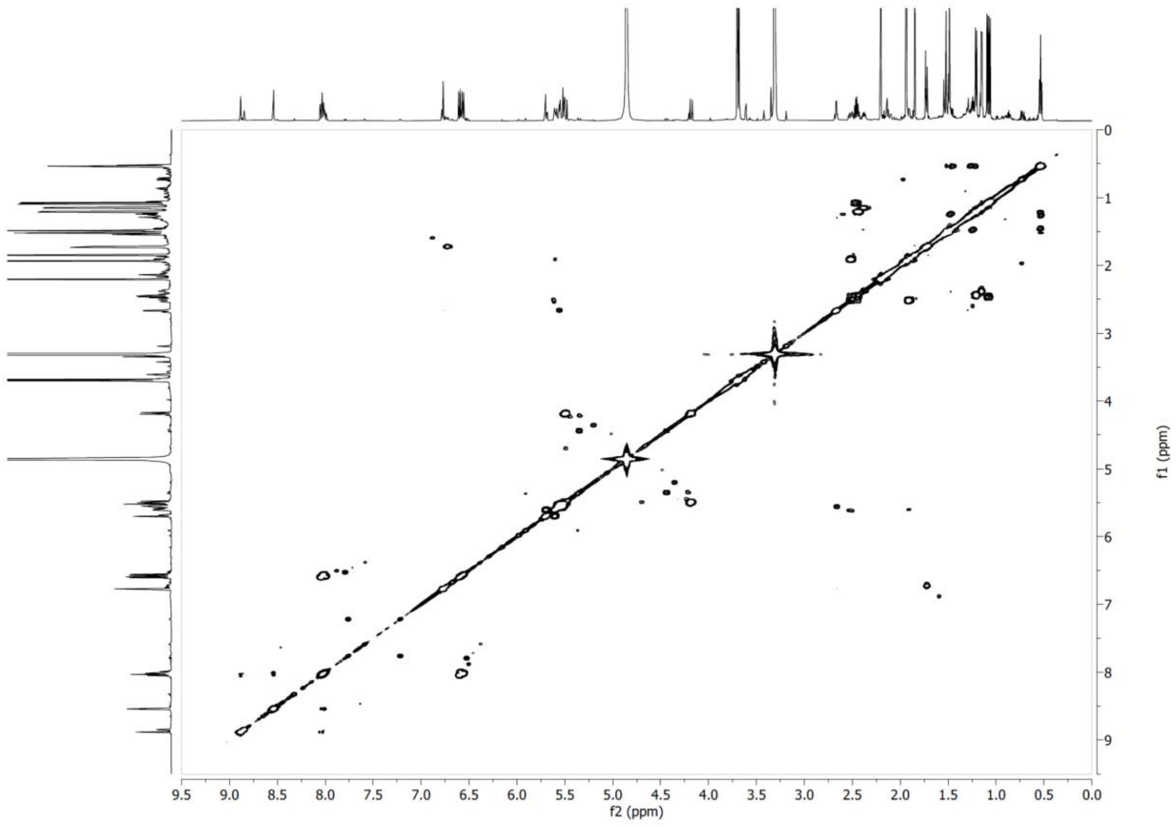

Supplementary Figure S54. COSY NMR spectrum of compound **10** in CD_3OD at 600 MHz.

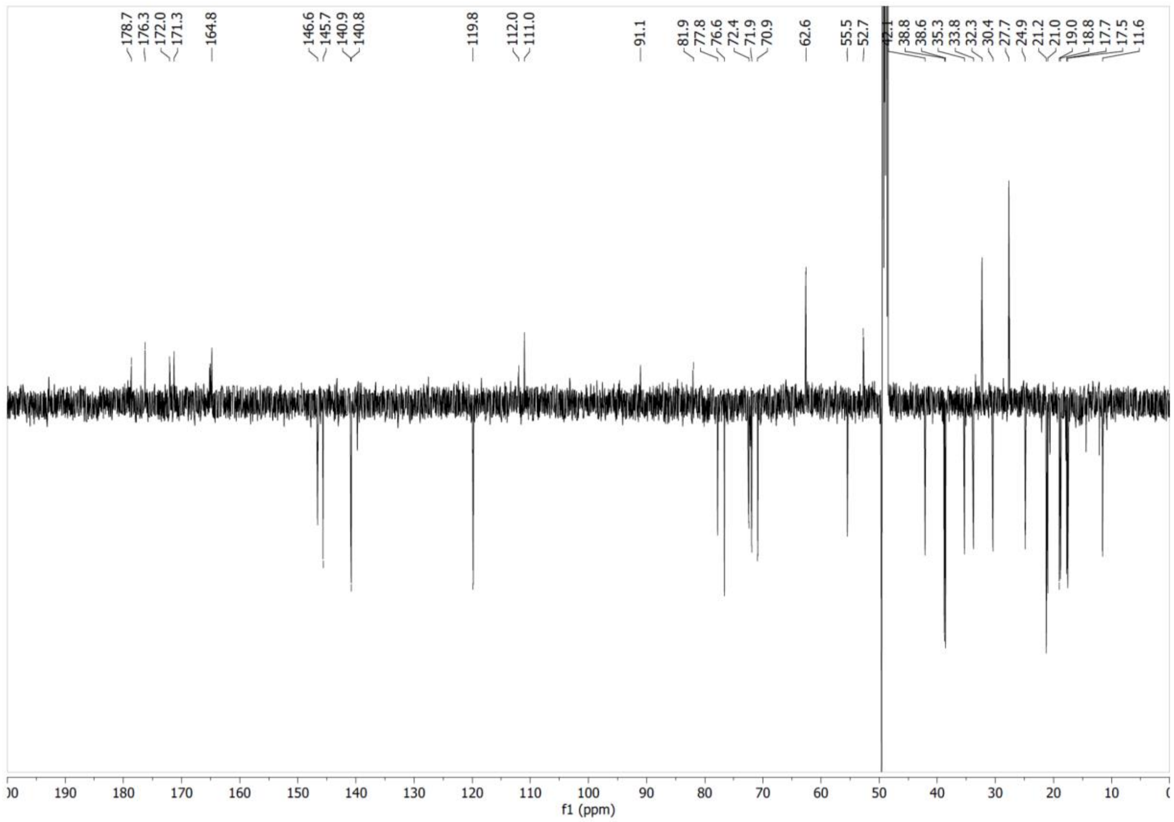

Supplementary Figure S55. ^{13}C NMR spectrum of compound **10** in CD_3OD at 151 MHz.

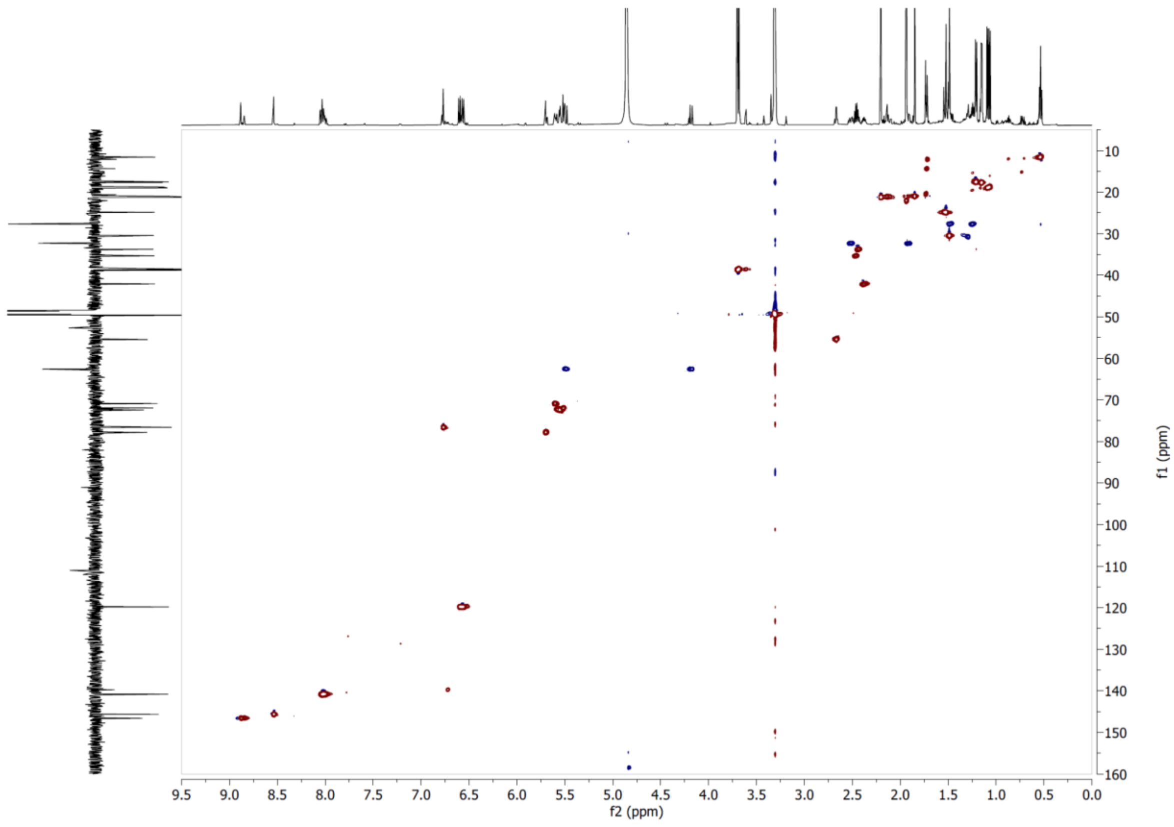

Supplementary Figure S56. HSQC NMR spectrum of compound **10** in CD_3OD.

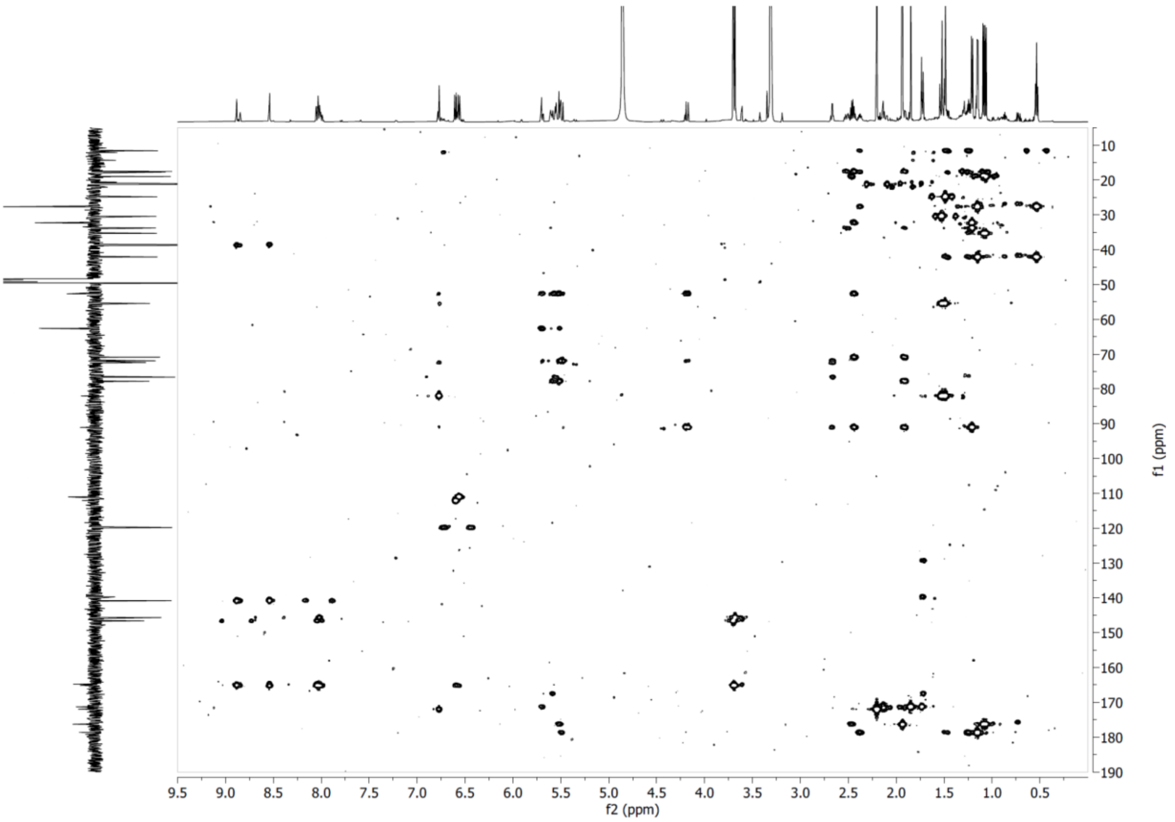

Supplementary Figure S57. HMBC NMR spectrum of compound **10** in CD_3OD.

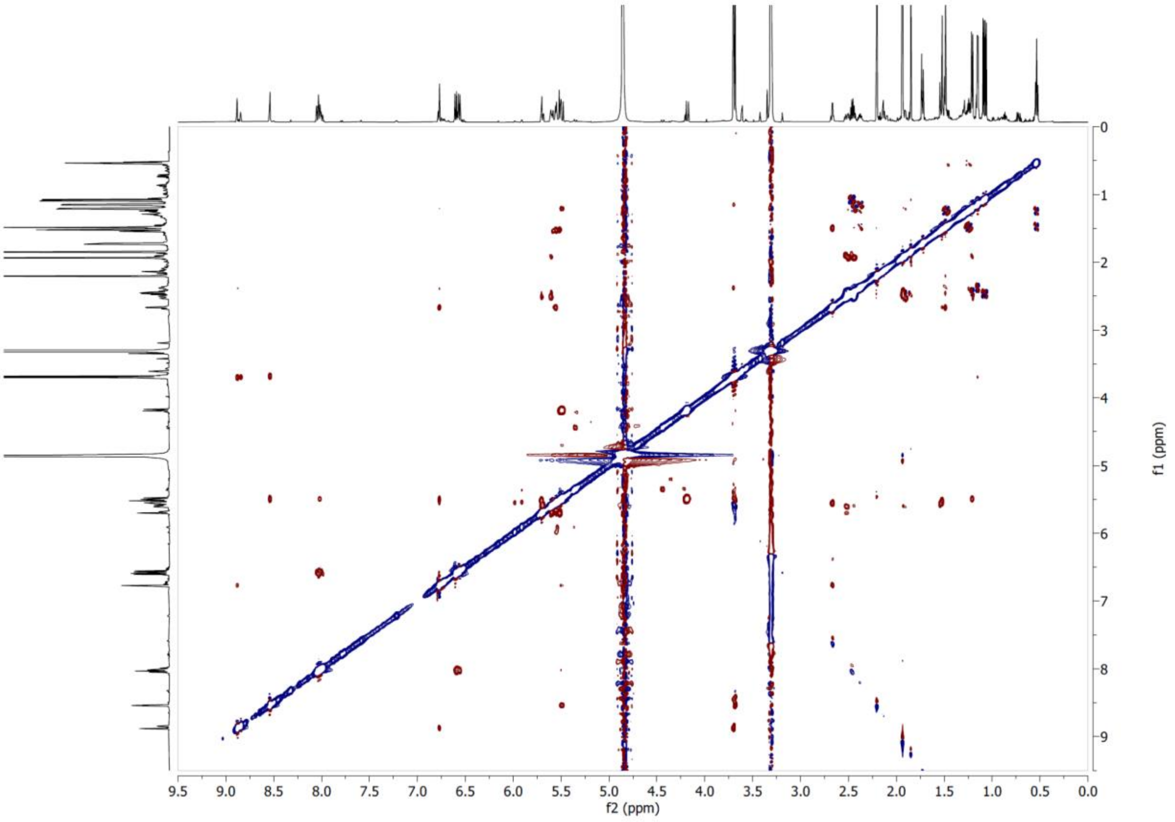

Supplementary Figure S58. ROESY NMR spectrum of compound **10** in CD_3OD at 600 MHz.

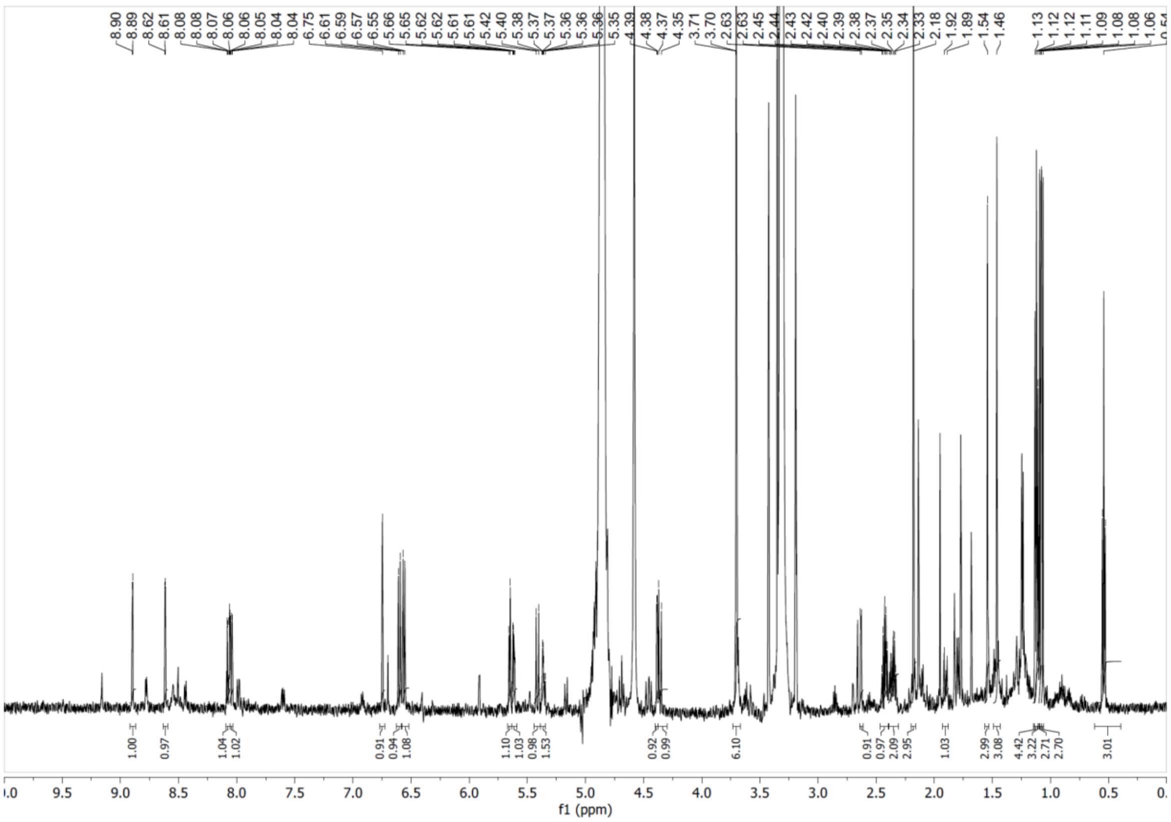

Supplementary Figure S59. ^{1}H NMR spectrum of compound **11** in CD_3OD at 600 MHz.

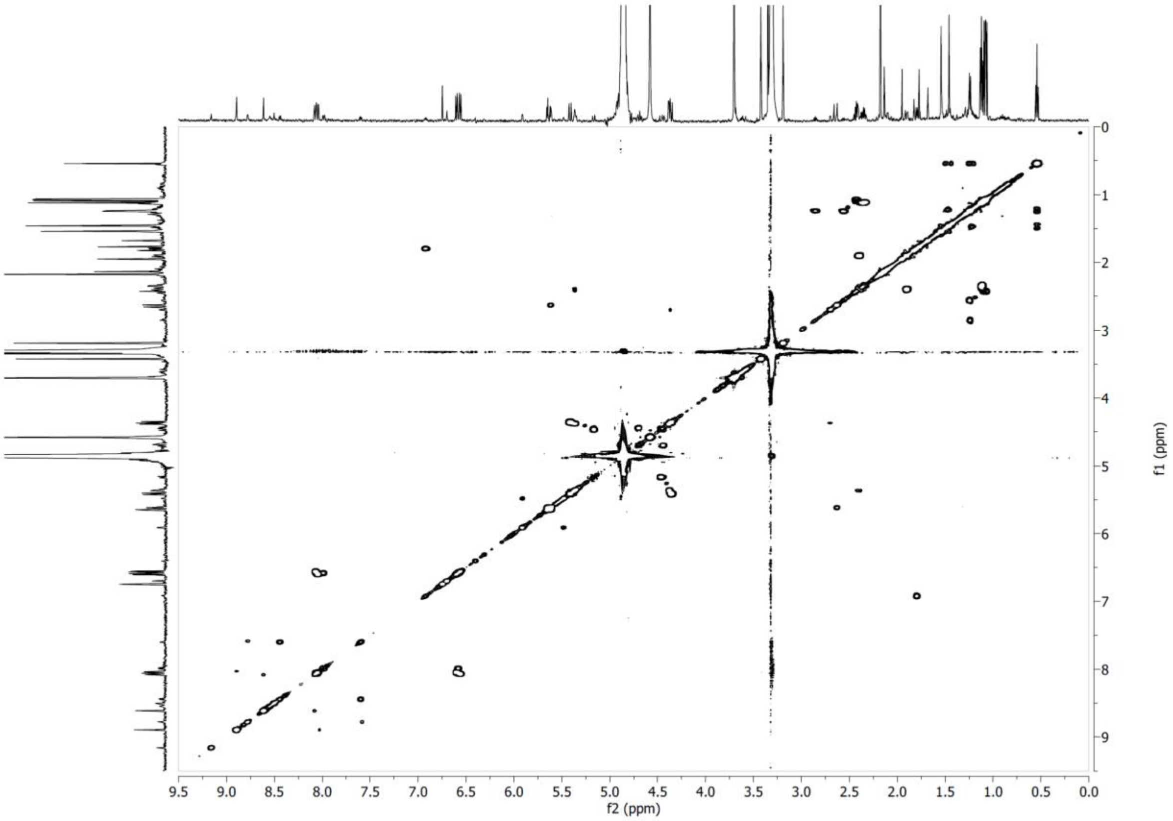

Supplementary Figure S60. COSY NMR spectrum of compound **11** in CD_3OD at 600 MHz.

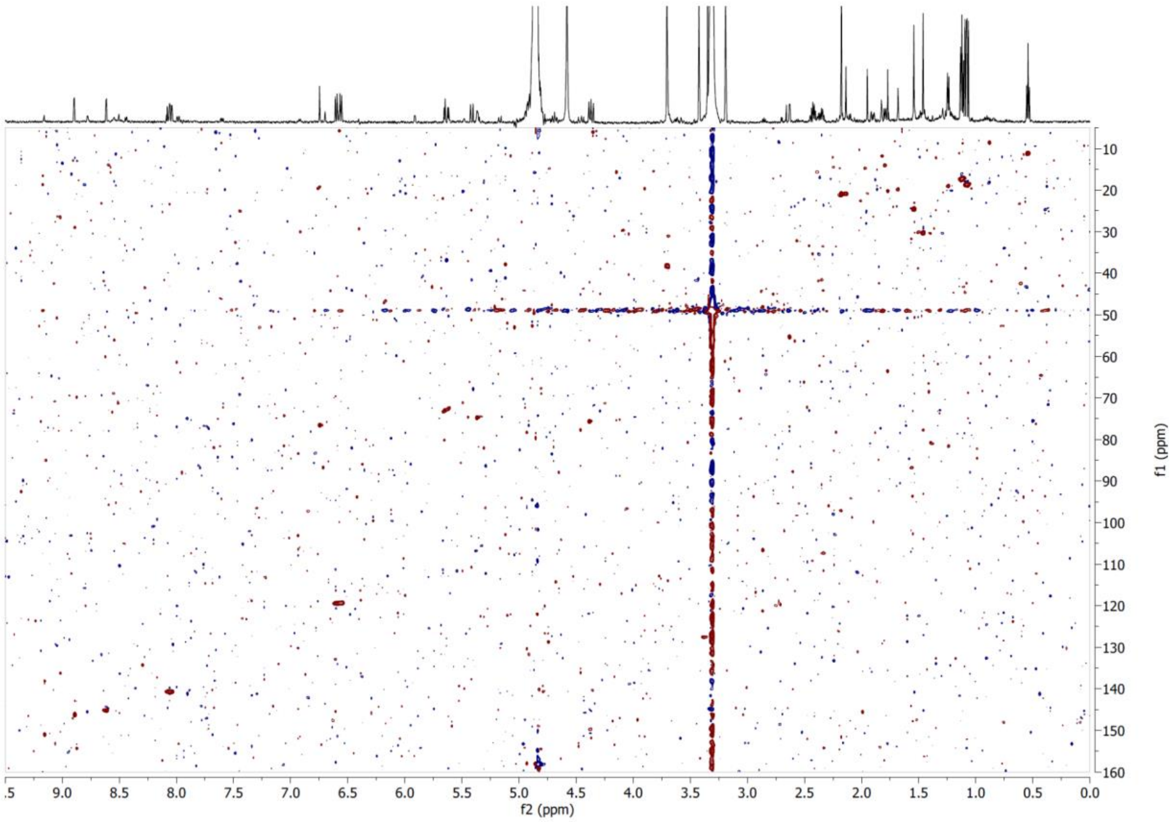

Supplementary Figure S61. HSQC NMR spectrum of compound **11** in CD_3OD.

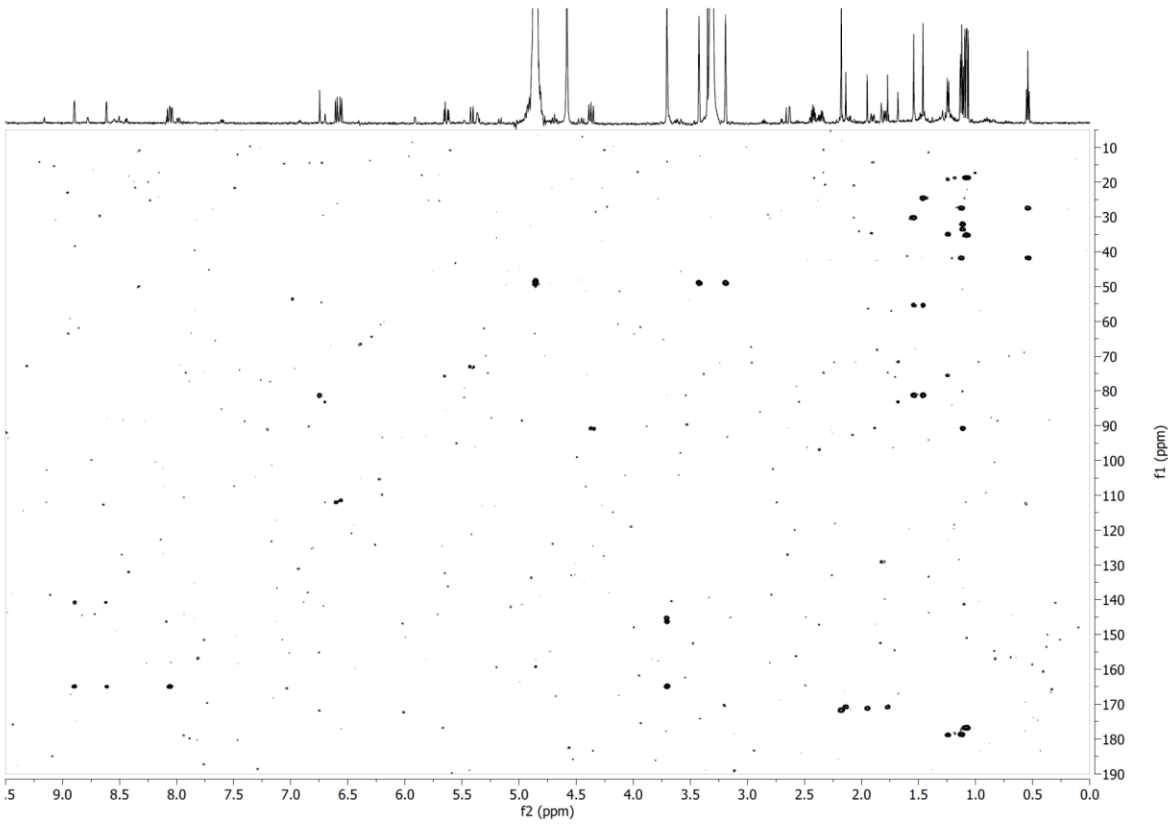

Supplementary Figure S62. HMBC NMR spectrum of compound **11** in CD_3OD.

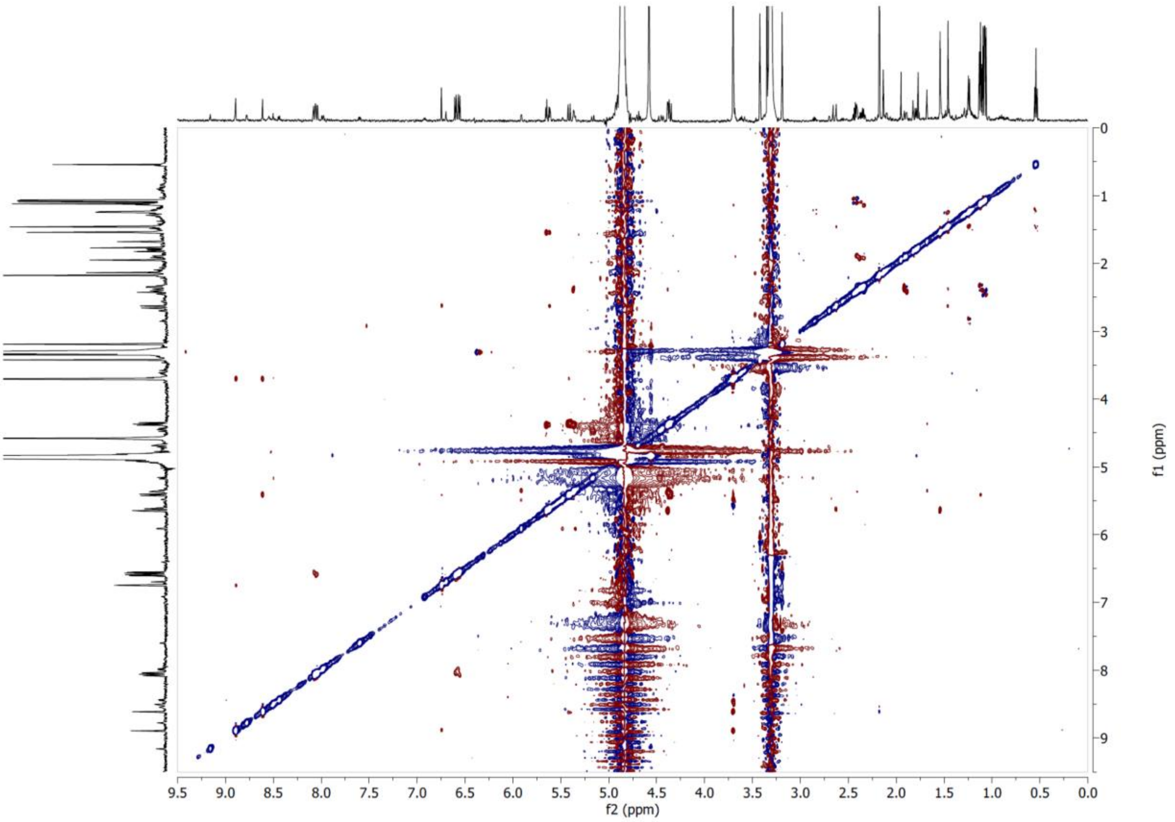

Supplementary Figure S63. ROESY NMR spectrum of compound **11** in CD_3OD at 600 MHz.

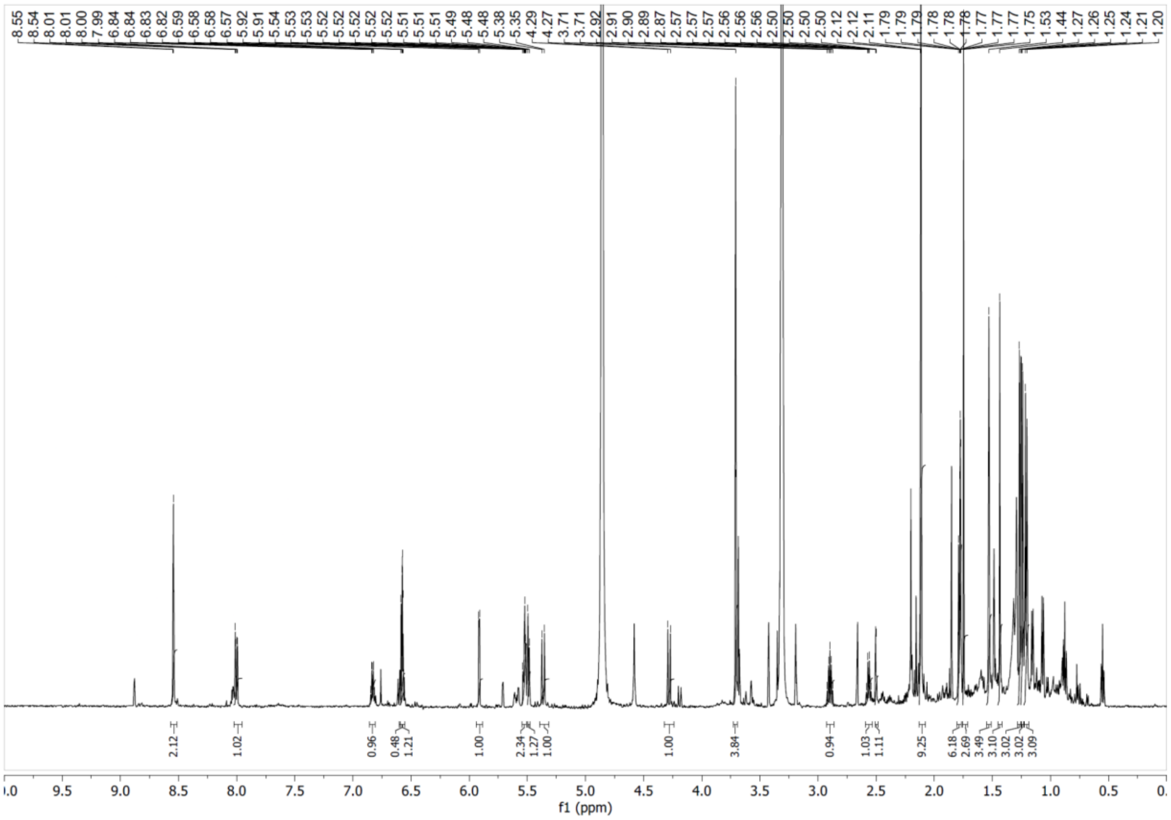

Supplementary Figure S64. ^1H NMR spectrum of compound **12** in CD_3OD at 600 MHz.

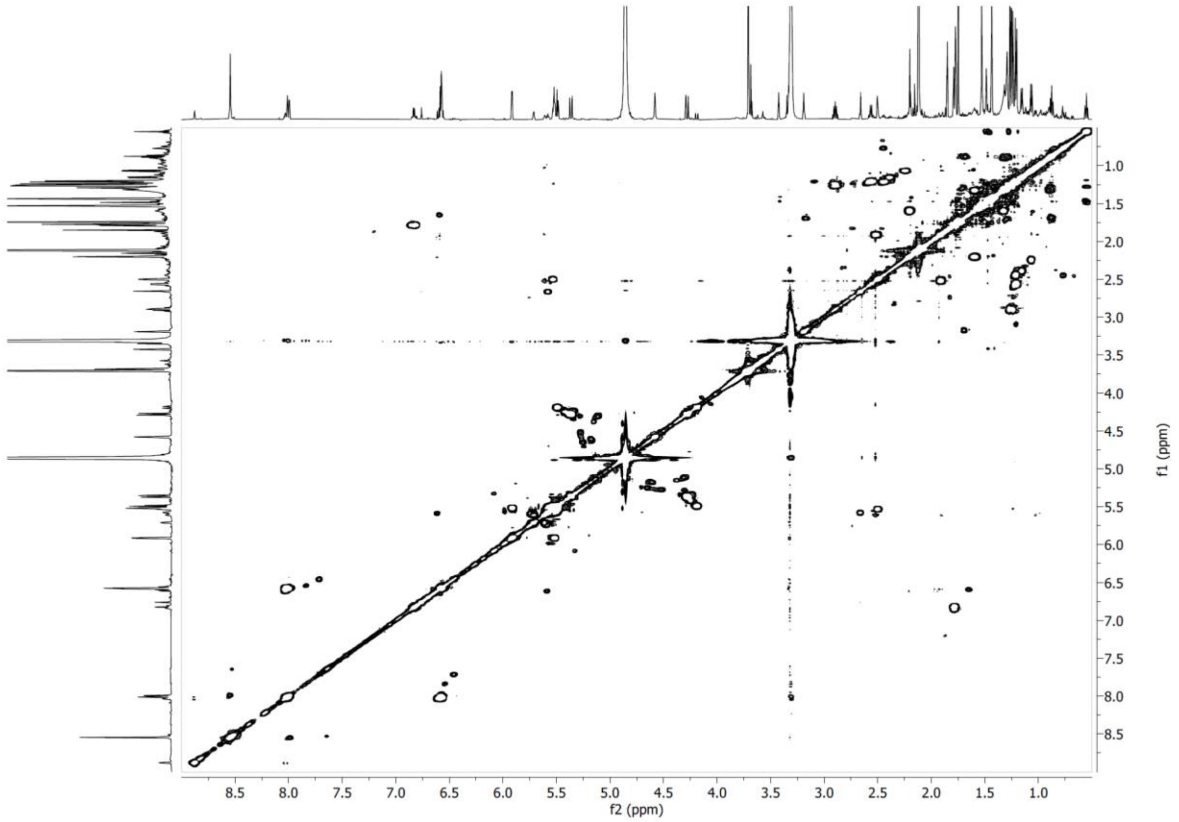

Supplementary Figure S65. COSY NMR spectrum of compound **12** in CD_3OD at 600 MHz.

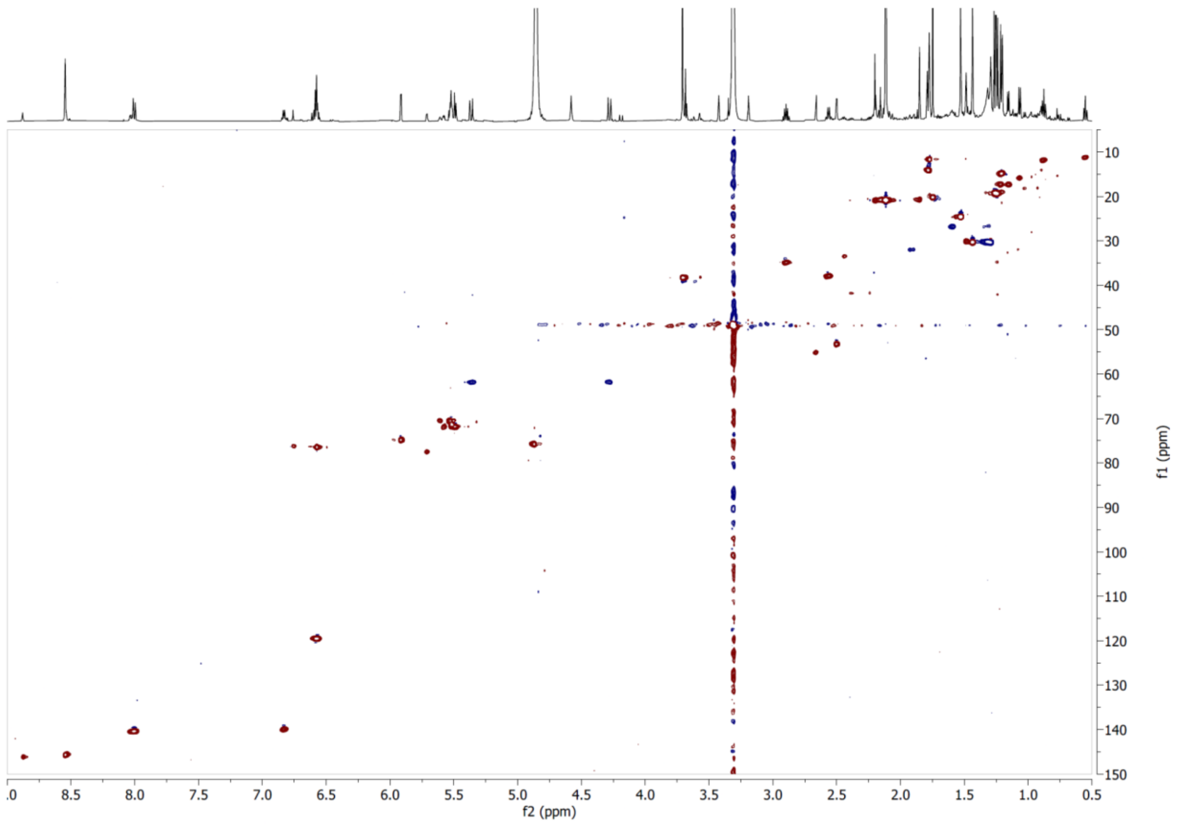

Supplementary Figure S66. HSQC NMR spectrum of compound **12** in CD_3OD.

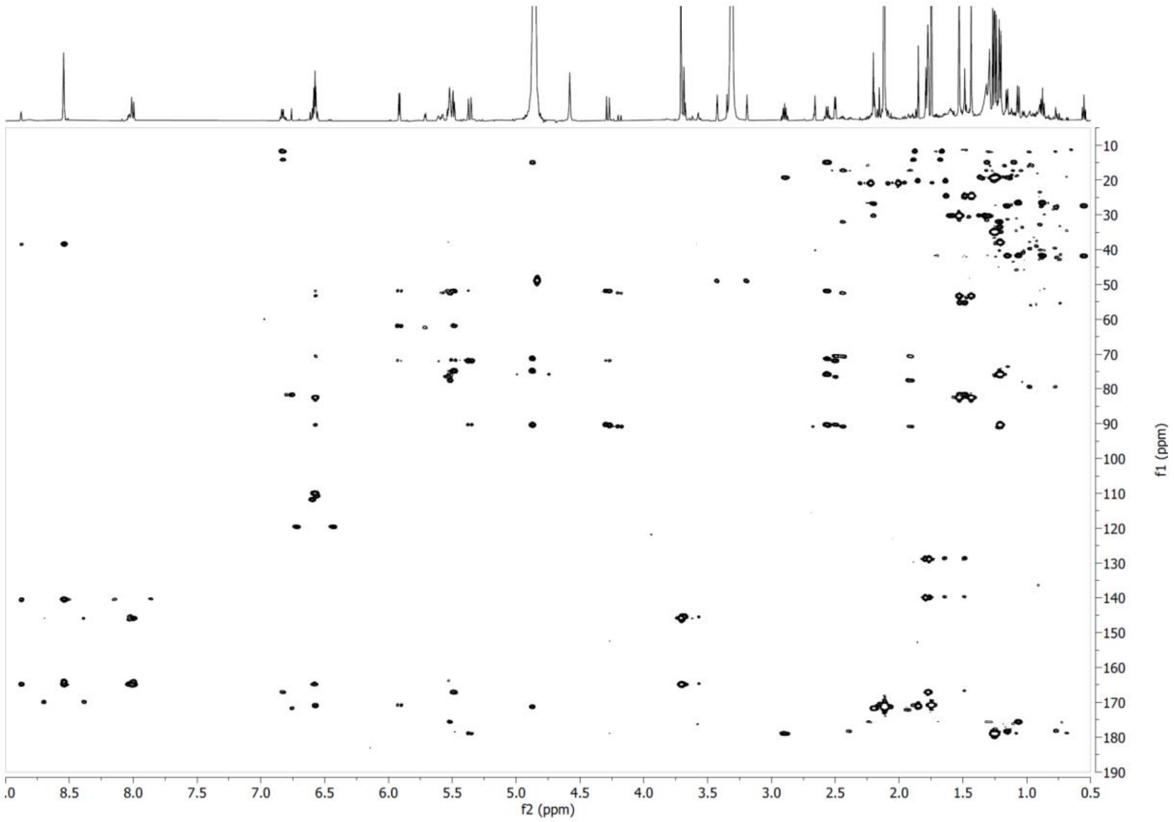

Supplementary Figure S67. HMBC NMR spectrum of compound **12** in CD_3OD.

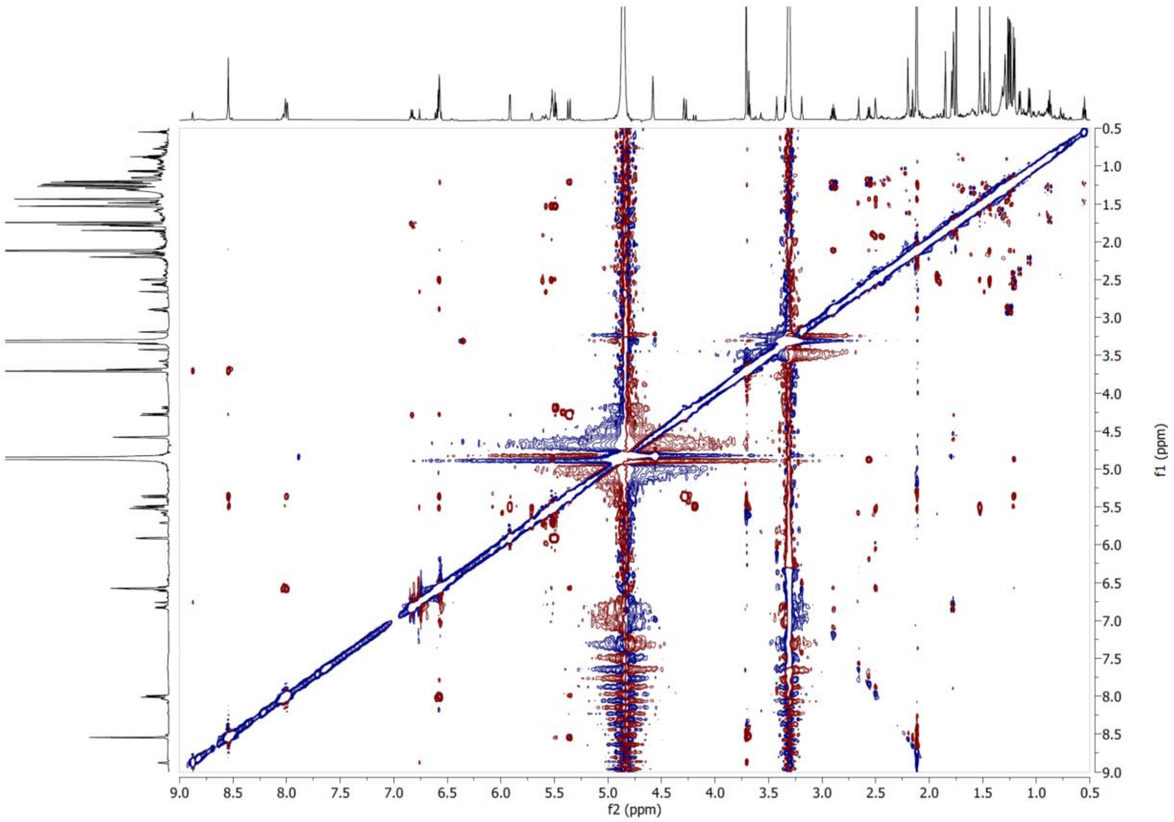

Supplementary Figure S68. ROESY NMR spectrum of compound **12** in CD_3OD at 600 MHz.

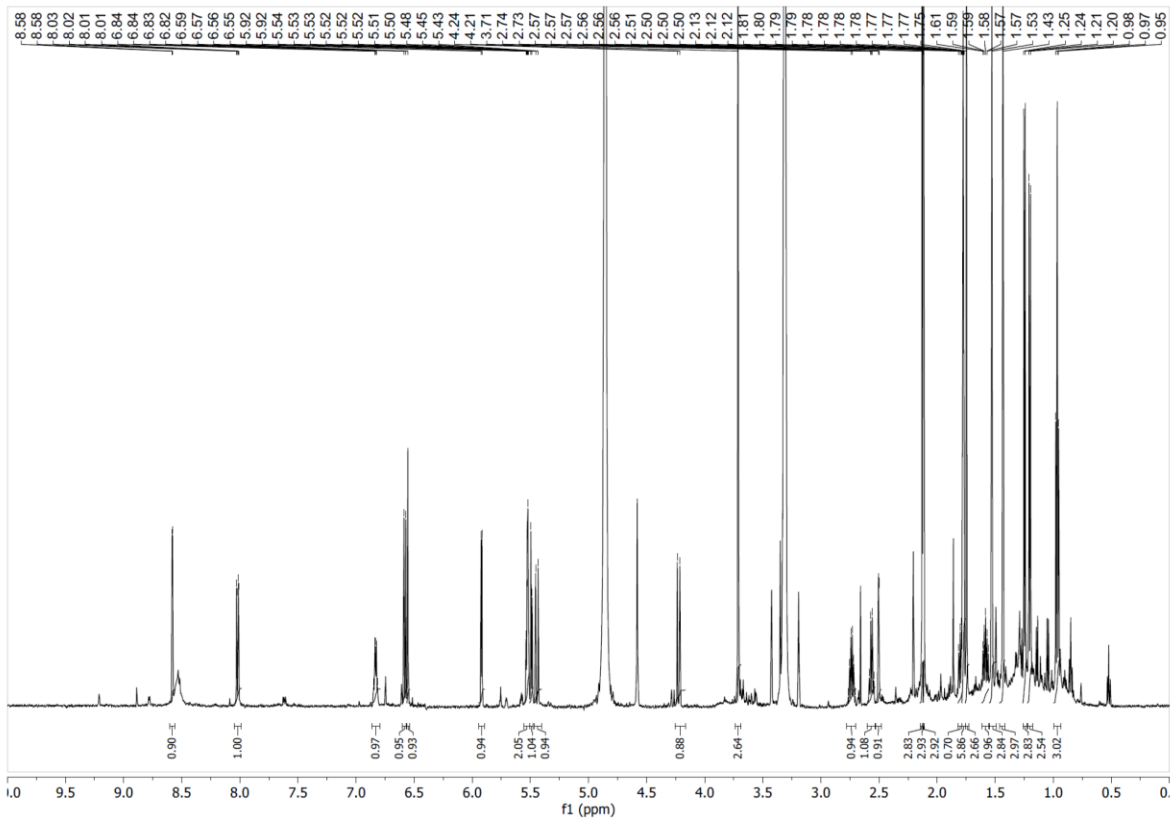

Supplementary Figure S69. ^{1}H NMR spectrum of compound **13** in CD_3OD at 600 MHz.

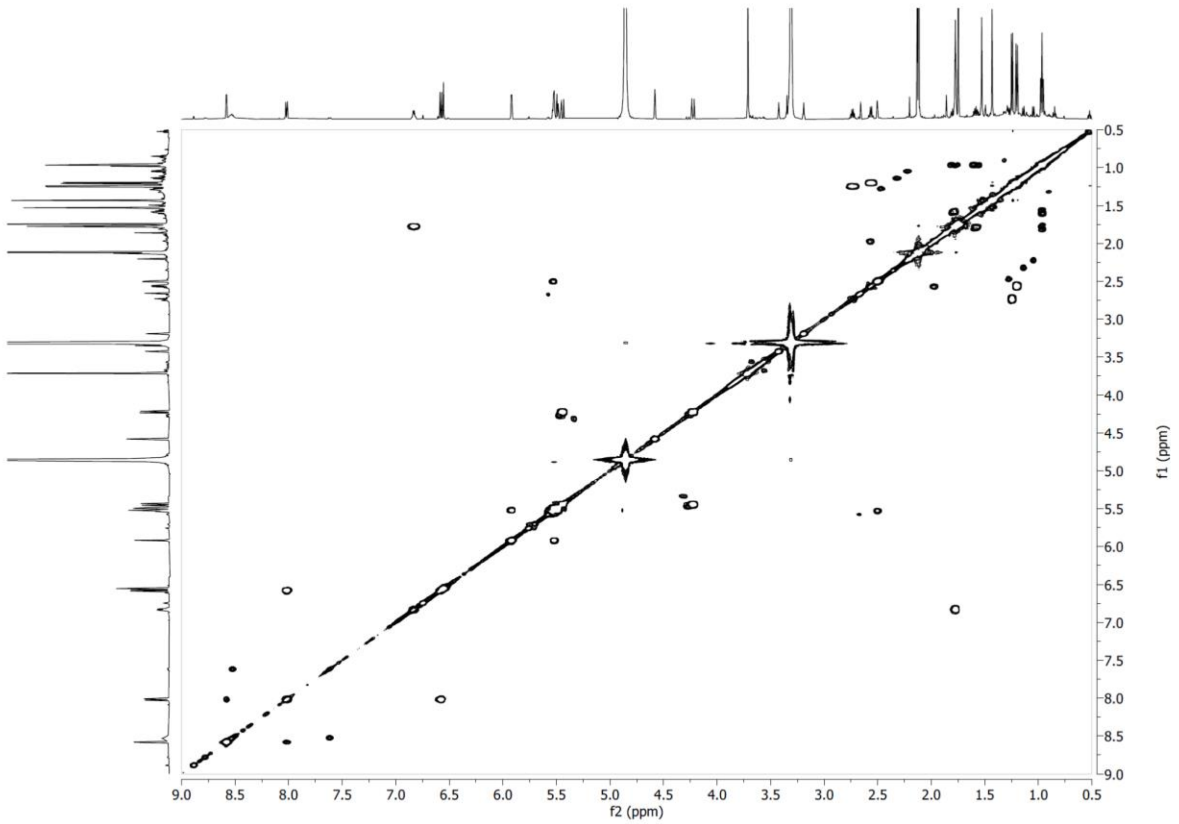

Supplementary Figure S70. COSY NMR spectrum of compound **13** in CD_3OD at 600 MHz.

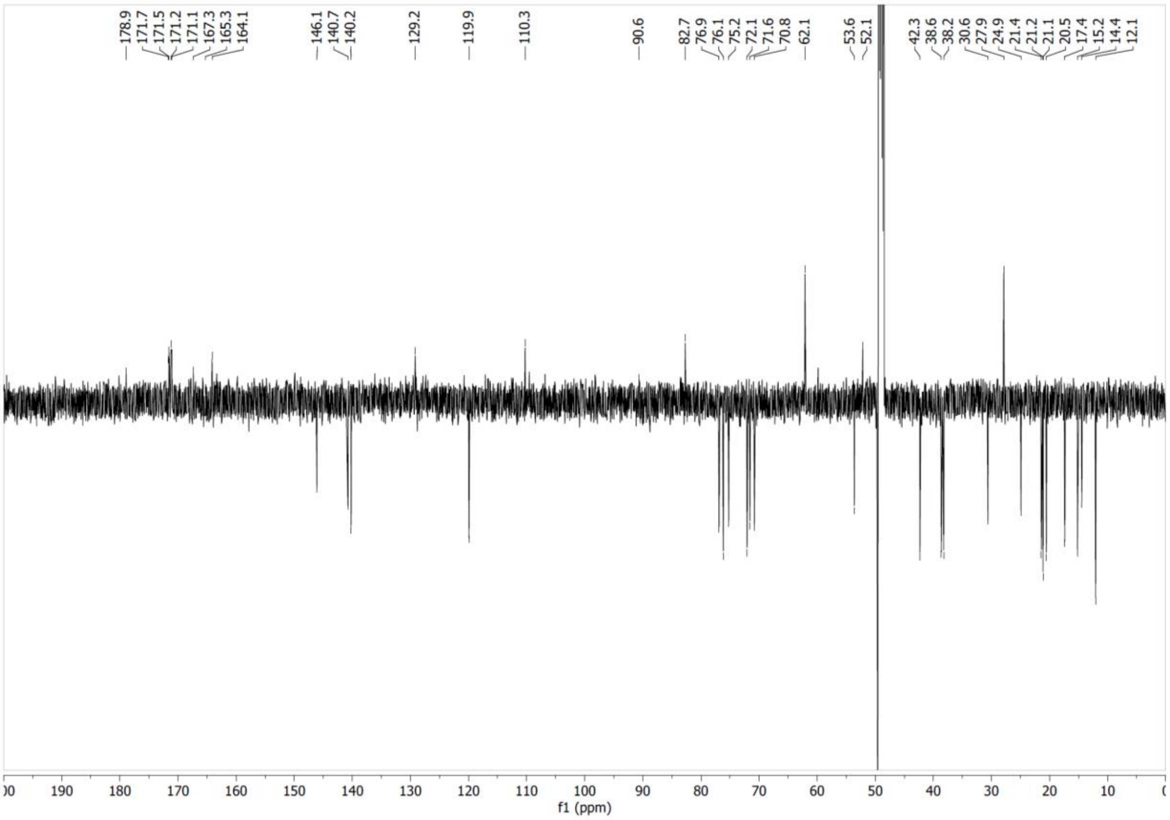

Supplementary Figure S71. ^{13}C NMR spectrum of compound **13** in CD_3OD at 151 MHz.

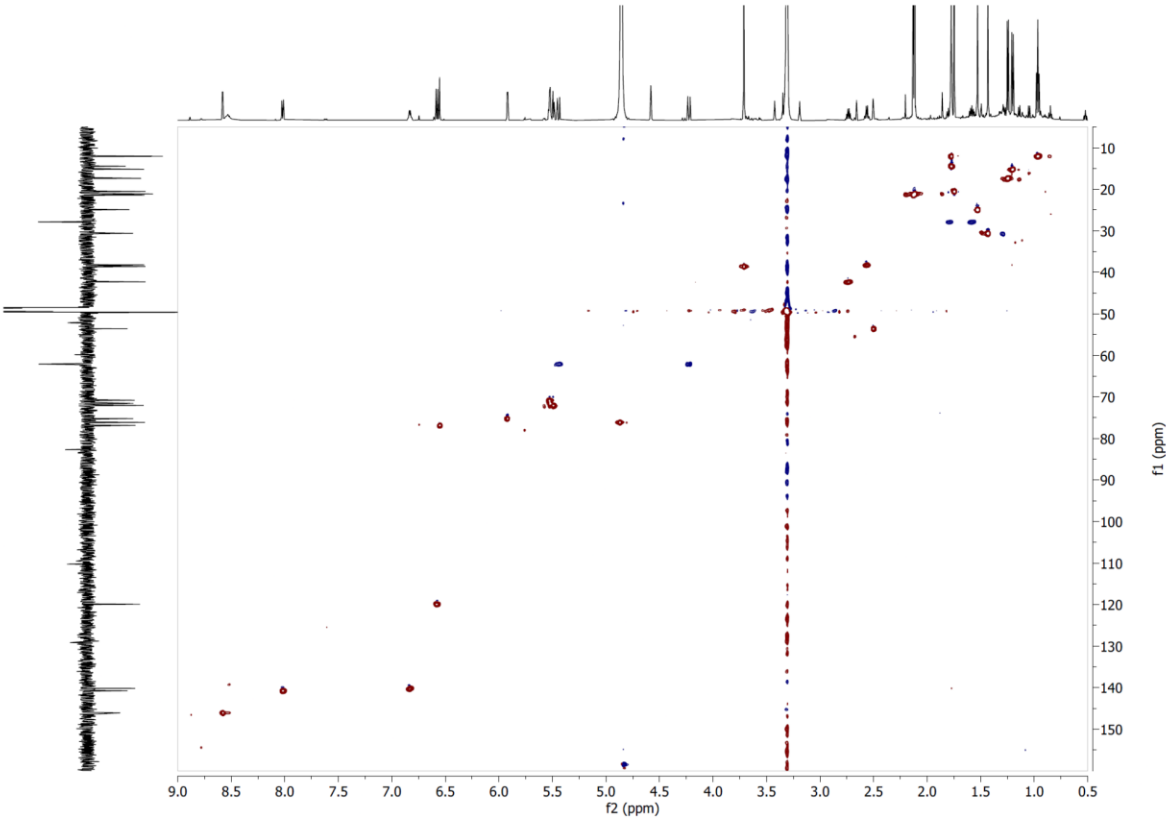

Supplementary Figure S72. HSQC NMR spectrum of compound **13** in CD_3OD.

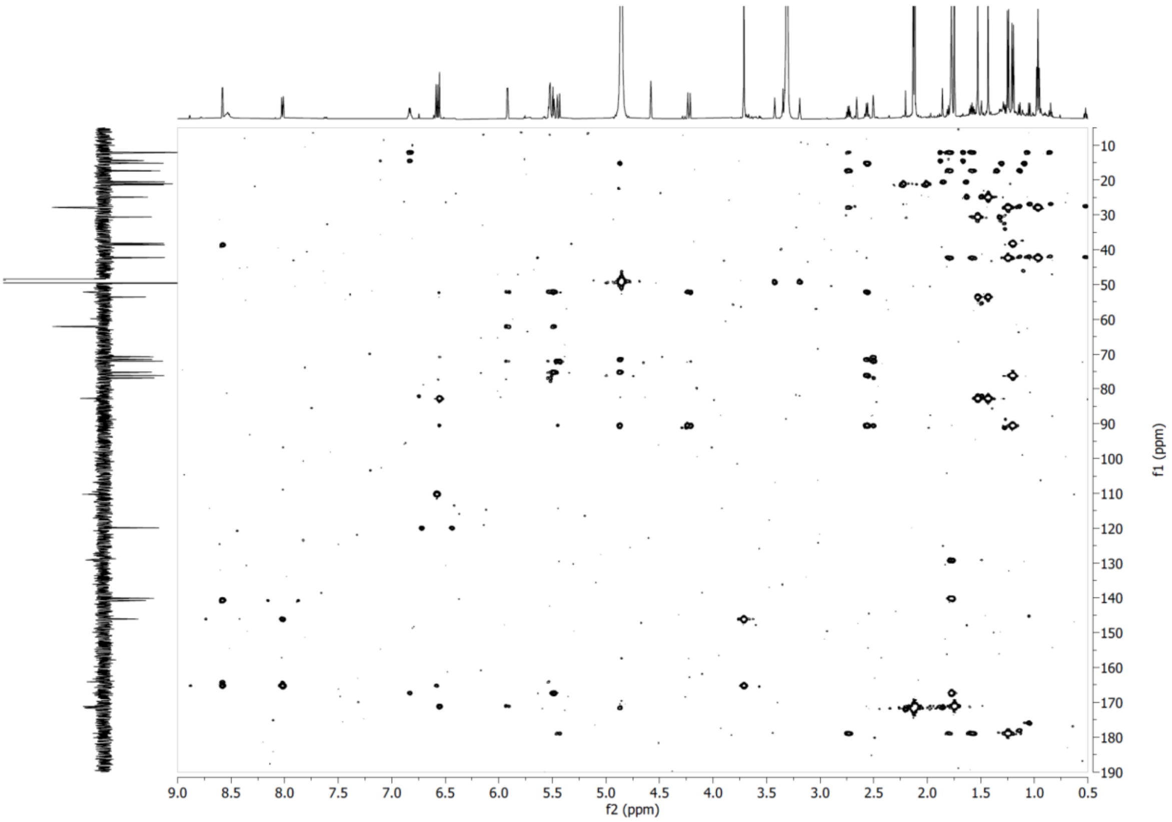

Supplementary Figure S73. HMBC NMR spectrum of compound **13** in CD_3OD.

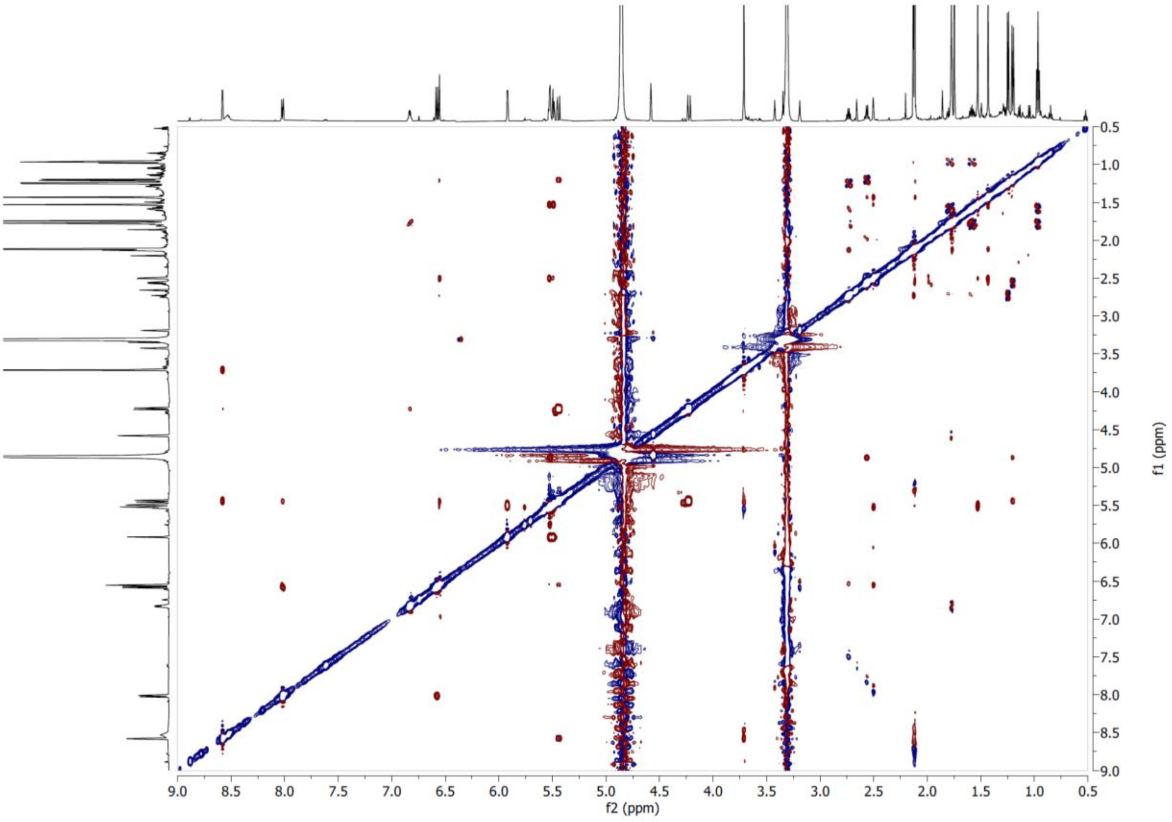

Supplementary Figure S74. ROESY NMR spectrum of compound **13** in CD_3OD at 600 MHz.

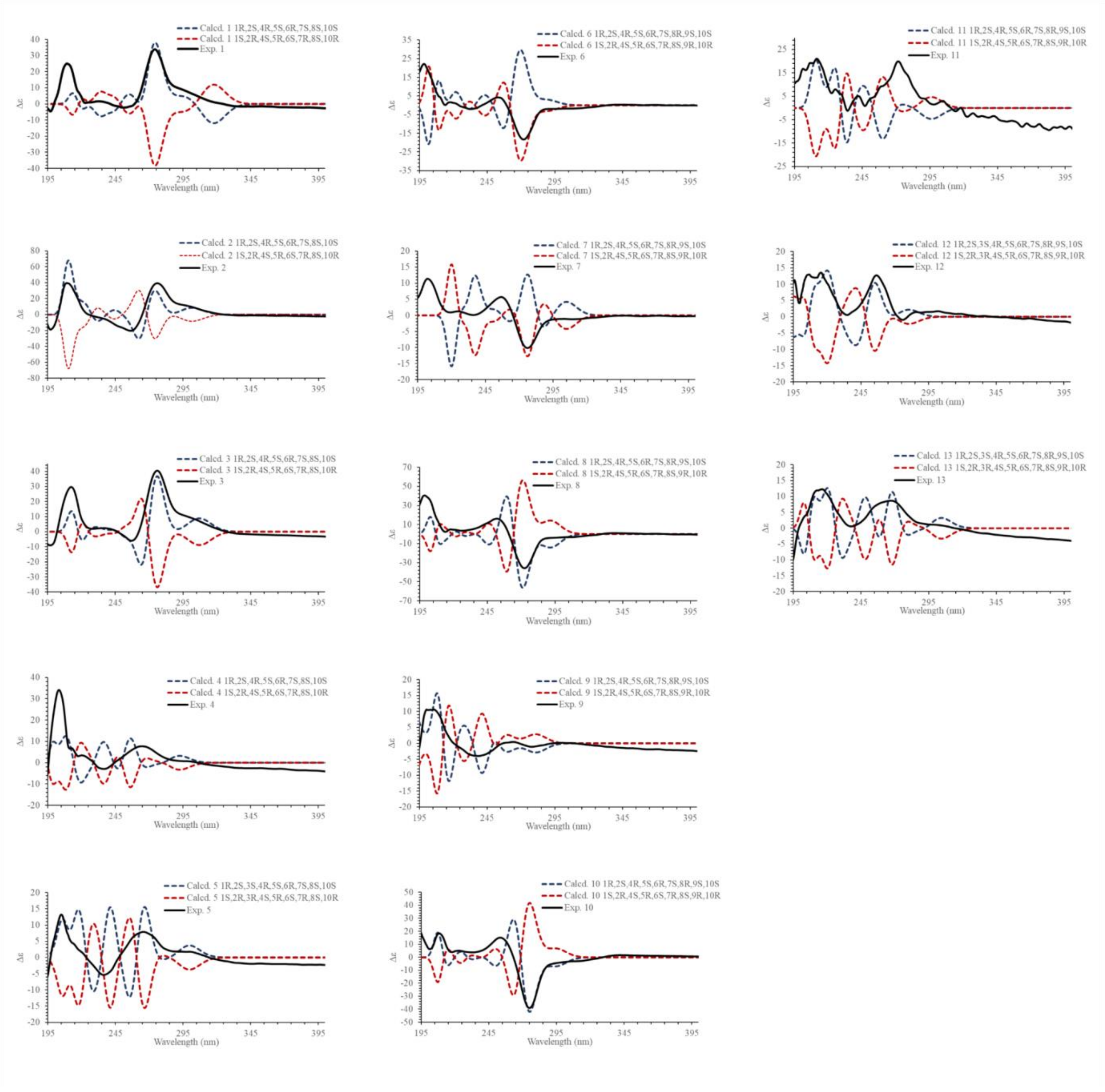

Supplementary Figure S75. Experimental and B3LYP/def2svp//B3LYP/6-31G(d,p) calculated spectra from compounds **1-13** in acetonitrile.

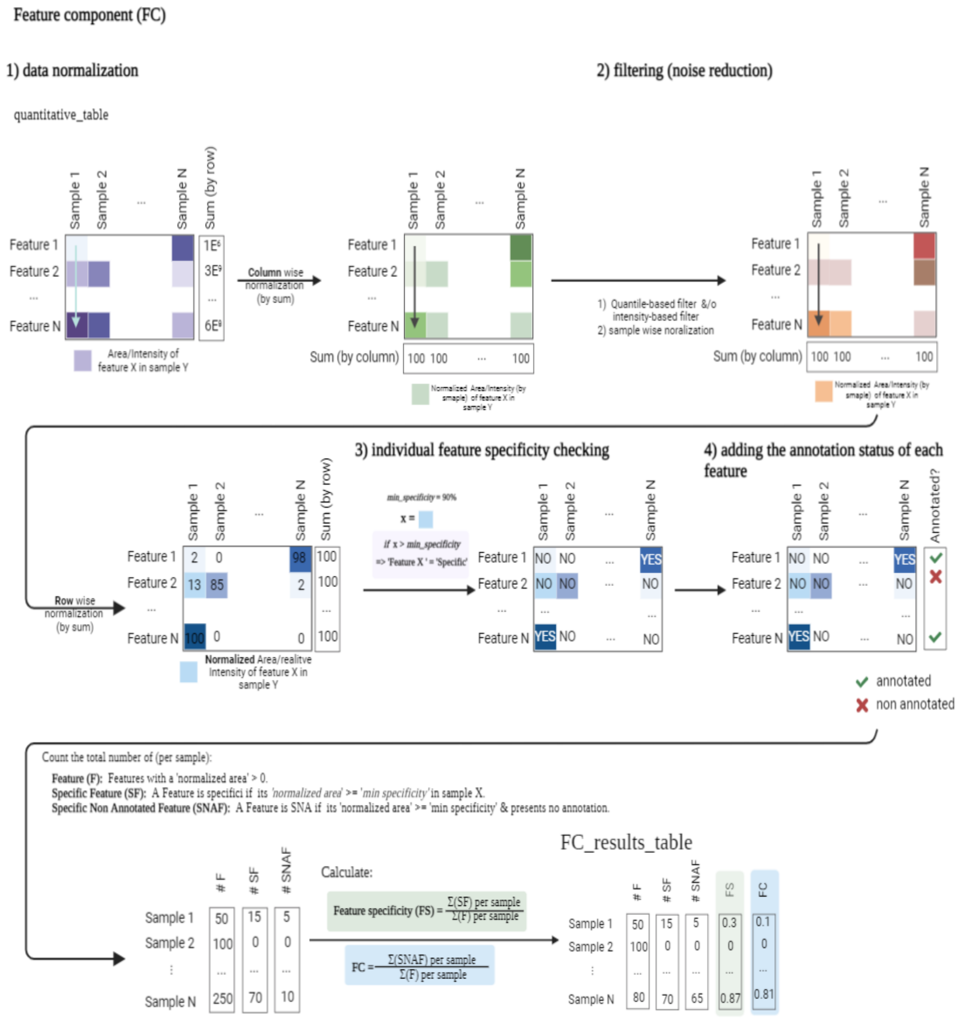

Supplementary Figure S76. Graphical representation of the steps followed for calculating the Feature Component (FC). The FC takes the feature-aligned quantification table obtained from the treatment of the raw data and the identification results from GNPS and other in silico tools as input. 1) The relative areas, based on the Total Ion Chromatogram, are normalized row-wise by total sum, to obtain relative percentages. 2) the resulting data frame is used to get the filename (or identifier preferred) of the sample where each ion is more concentrated (higher percentage). 3) The annotation status of each feature is linked to the results from point 2. For this particular step, several inputs from different annotation bioinformatics tools can be considered. Results from the GNPS platform are higher in relevance since they are based on an experimental database. *In silico* dereplication results from ISDB or SIRIUS can be included. If so, the script will consider the annotation status of a particular feature like 'annotated' if it presents identification in any of the inputs used. 4) The specificity status of each feature is checked according to the *min_specificity* threshold fixed by the user. If the area of a particular feature is equal to or higher than *min_specficity* then the feature is consider as 'specific'. 5) once both conditions are established for each feature (annotations and specificity status), the feature component ratio is calculated. The ratio is the division of the total of specific-non annotated features in a sample by the total of features present in it. Additionally, the Feature specificity is calculated in a similar way, but without taking into account the annotation status, only the specificity status. This means that the features specificity corresponds to the division of the total of specific features in a sample by the total of features present in the sample. The difference between both values indicates

the percentage of specific ions in the sample presenting an annotation. The molecular formula prediction ratio (MF_prediction_ratio) is calculated in the same way, but adding a particular condition, ‘the feature presents a good quality predicted molecular formula’. This molecular formula comes from the dereplication process using the Sirius software. It proposes a molecular formula using the module ZODIAC. So, MF prediction ratio corresponds to the division of the total of specific-non annotated-’has a good quality MF’ features in a sample by the total of features present in the sample. Refer to **Supplementary Table 1** for actual results.

<>

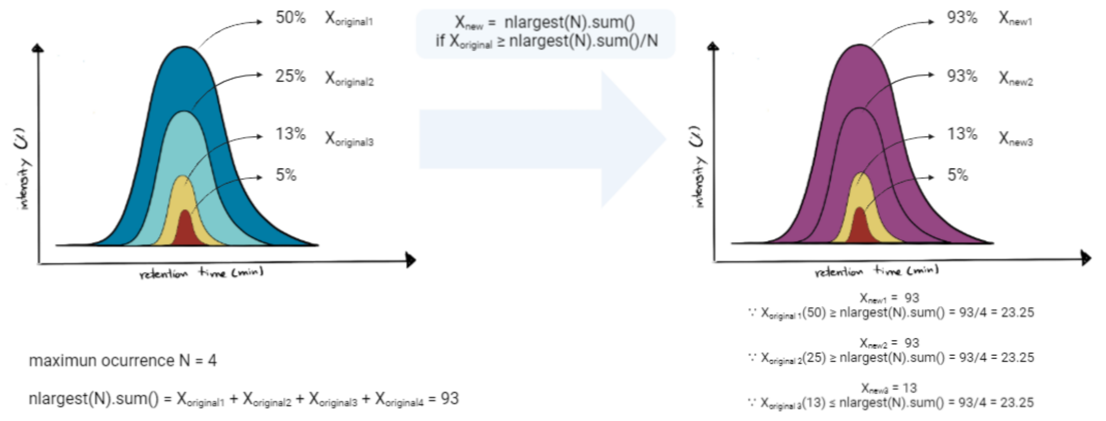

Supplementary Figure S77. Graphical representation of the data handling is performed when multiple samples with the same species are present in the dataset. The user will define the maximum occurrence of the species (N), this value will be used to get the sum of the N largest values for each feature in the table [nlargest(N).sum]. As shown in the figure, as an example for N = 4, the nlargest(N).sum = 93%, which means that this feature is potentially specific to the species. Because the selection and counting of specific features are done sample-wise, the original values of the feature in the N largest contributing samples are temporarily replaced by the (X_{new} = nlargest(N).sum). This, is only done if the original value ($X_{original}$) is higher than nlargest(N).sum/N. In the example, only the 2 maximum values were replaced accordingly. This restriction avoids introducing the presence of a specific feature in a sample where the feature is in a low proportion (way more difficult to isolate). After the replacement of the values, the calculations for FS and FC are done as previously described.

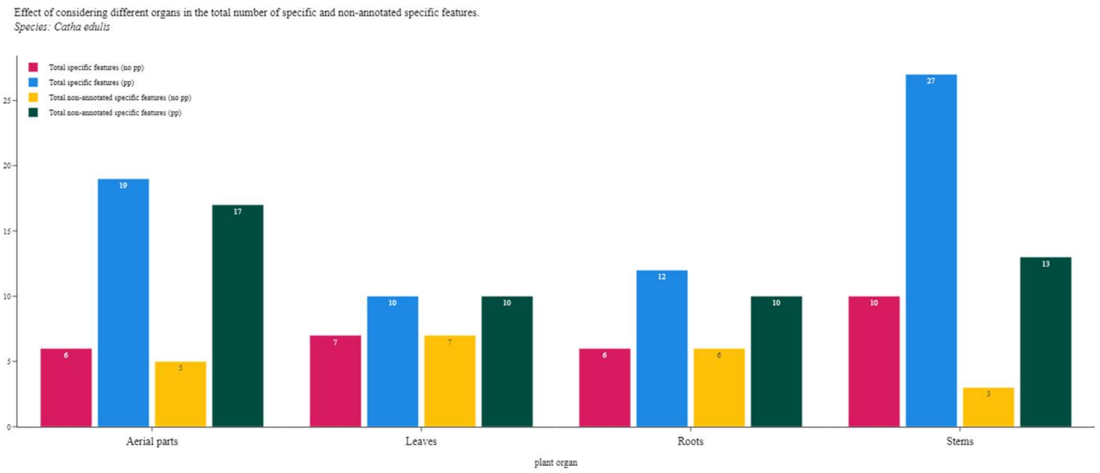

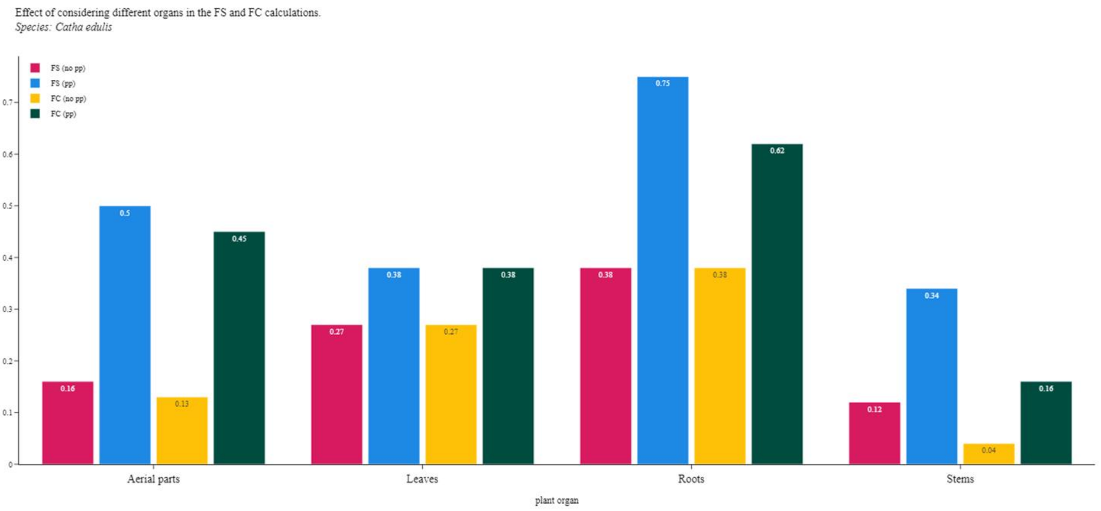

Supplementary Figure S78. Upper trace: Effect of considering the presence of different organs for the same species on the total number of specific and non-annotated specific features\. Lower trace: Effect of considering the presence of different organs for the same species on the FS and FC results.

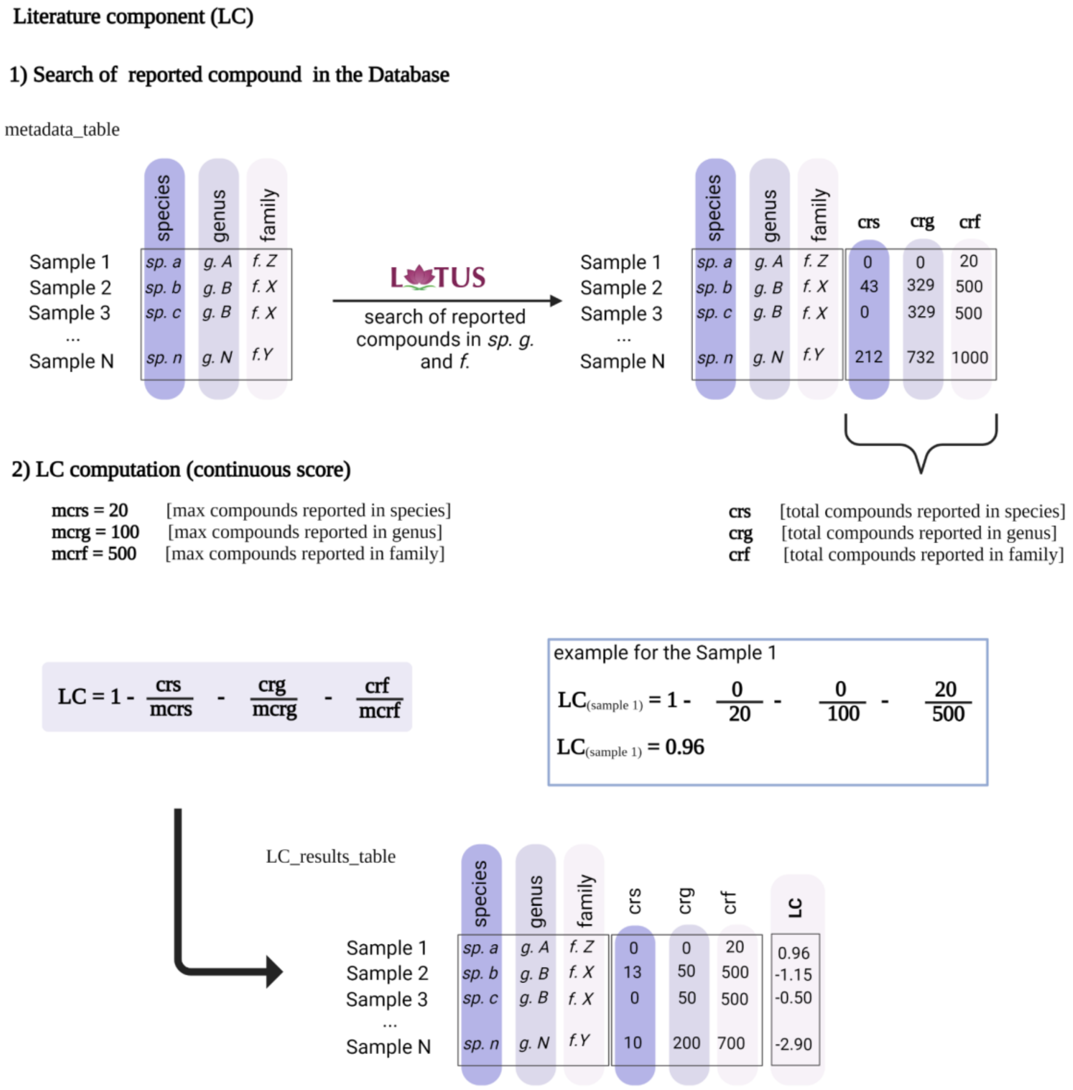

Supplementary Figure S79. Graphical representation of the steps followed for the calculation of the Literature Component (LC). The LC takes the taxonomical information for each sample as input. The species should be cleaned-up to have up to date recognized names, the Open Tree of Life (OTL), including the genus and family for each species. 1) The script will create a set{} with the species, genus and families found in the metadata to recover the total number of compounds reported in the database LOTUS for each level, and link the results directly to the samples. 2) The LC score is calculated with the formula shown above. The score is going to be modulated according to the maximum number of reported compounds in species (mrcs), genus (mrcg) and family (mcrf) defined by the user. The larger the number of reported compounds at each level, the lower the LC value.

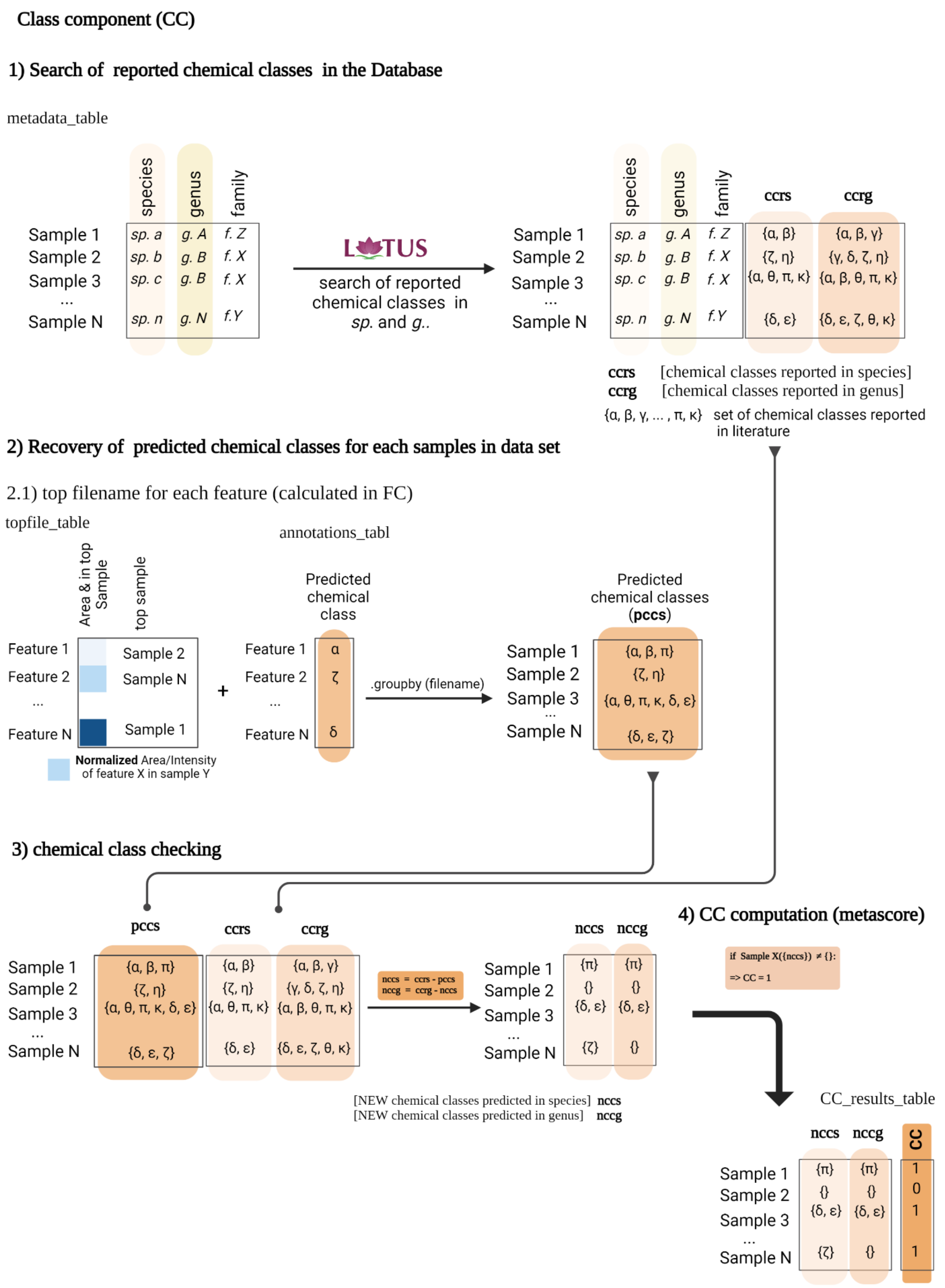
Class component (CC)
1) Search of reported chemical classes in the Database
metadata_table
species
genus
family
Sample 1
Sample 2
Sample 3
Sample N
sp. a g. A f. Z
sp. b g. B f. X
sp. c g. B f. X
sp. n g. N f.Y
LOTUS
search of reported chemical classes in sp. and g..
ccrs
ccrg
{α, β} {α, β, γ}
{ζ, η} {γ, δ, ζ, η}
{α, θ, π, κ} {α, β, θ, π, κ}
{δ, ε} {δ, ε, ζ, θ, κ}
ccrs [chemical classes reported in species]
ccrg [chemical classes reported in genus]
{α, β, γ, ... , π, κ} set of chemical classes reported in literature
2) Recovery of predicted chemical classes for each samples in data set
2.1) top filename for each feature (calculated in FC)
topfile_table
annotations_tabl
Area & in top Sample
top sample
Feature 1 Sample 2
Feature 2 Sample N
Feature N Sample 1
Normalized Area/Intensity of feature X in sample Y
Predicted chemical class
Feature 1 α
Feature 2 ζ
Feature N δ
.groupby (filename)
Predicted chemical classes (pccs)
{α, β, π}
{ζ, η}
{α, θ, π, κ, δ, ε}
{δ, ε, ζ}
3) chemical class checking
pccs
ccrs
ccrg
nccs = ccrs - pccs
nccg = ccrg - nccs
nccs
nccg
{π} {π}
{} {}
{δ, ε} {δ, ε}
{ζ} {}
[NEW chemical classes predicted in species] nccs
[NEW chemical classes predicted in genus] nccg
4) CC computation (metascore)
if Sample X({nccs}) ≠ {}:
=> CC = 1
CC_results_table
CC
1
0
1
1

Supplementary Figure S80. Graphical representation of the steps followed for calculating the Class Component (CC). The CC takes the taxonomical information for each sample, the quantitative table, and the dereplication results from SIRIUS-CANOPUS as input. The species should be cleaned-up to have up to date recognized names, the [Open Tree of Life](#) (OTL), including the genus and family for each species, this information is the same used for computing the LC. 1) The script will create a set{} with the species, genus, and families found in the metadata to recover the total of chemical classes for all the compounds reported in LOTUS for each level, and link the results directly to the samples. 2) Because the dereplication information comes from an aligned dataset, it is necessary to find to which sample a particular chemical class belongs, to simplify this, the table calculated in the second step of the FC (**Supplementary Figure 76**) is used, it contains the filename (sample) where each feature is present at the highest percentage. The CANOPUS results are linked to this table and the results are grouped by sample, generating a set of chemical classes predicted for each sample (pccs). 3) The new chemical classes predicted in species (nccs) and in genus (nccg) are obtained by simple subtraction (nccs = ccrs - pccs; nccg = ccrg - nccs). 4) The script will look into the nccs column to check if it is empty or contains any value (string), if there is some chemical class the Metascore CC for that particular sample will be assing as ‘1’, otherwise is ‘0’.

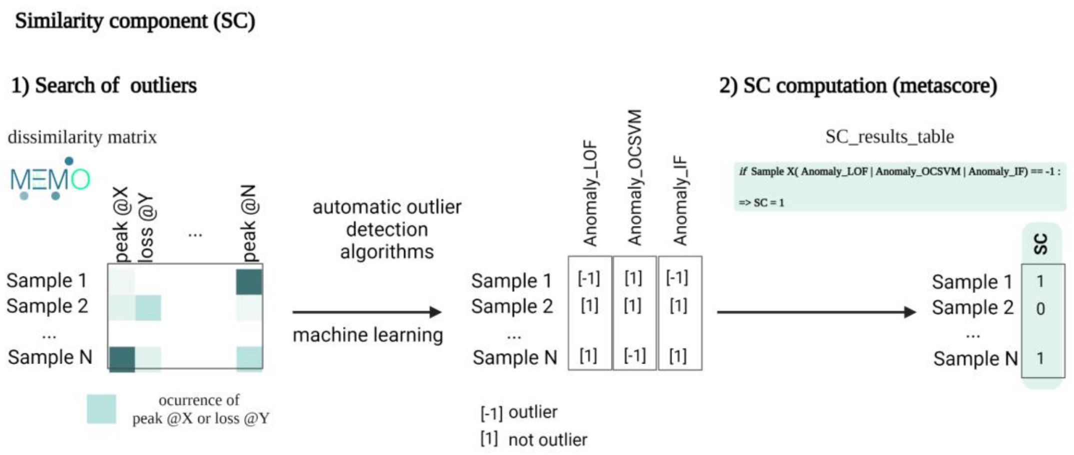

Supplementary Figure S81. Graphical representation of the steps followed for calculating the Similarity Component (SC). The SC takes the dissimilarity matrix generated from memo ms as input. 1) Three automatic outlier detectors are run over the set without further parameters to fix. The general parameters were fixed to 'auto'. However, depending on the user and the particularities of the set itself multiple parameters could be tunned. The script will add a column for the results of each of the three algorithms indicating the status, [-1] = outlier, [1] normal point. 2) Once the samples are tagged, the script will evaluate the status before adding the Metascore value '1' for outliers and '0' for normally distributed points accordingly. As explained in the main text, we used three different algorithms to cover a wider range of outliers predictions, the score will be '1' for the samples being 'outlier, [-1]' at least in one of the three algorithms.

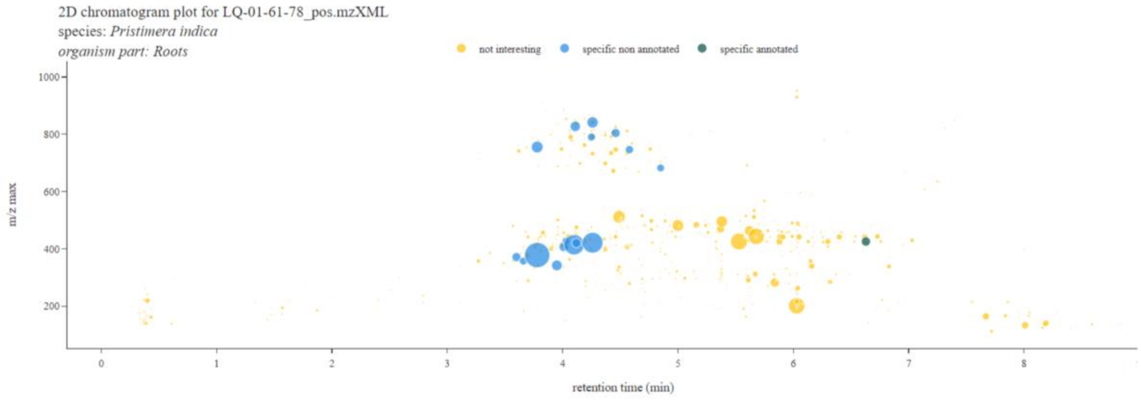

Supplementary Figure S82. Feature-based interactive Ion map plots showing the combined results of the FC and CC (if calculated) for the features of a selected sample (user-defined). The features are displayed according to their status (specific non-annotated (blue), specific annotated (green), and non-specific non-annotated -not interesting- (yellow)). Complementary information (adducts, row id, chemical class, etc) are displayed interactively for each feature if available. The intensities in both cases (bar's height and bubble's size) are proportional to the original quantification table. The scatter plot shows the *m/z* ratio of each feature (or ion network identity) on the y-axis.

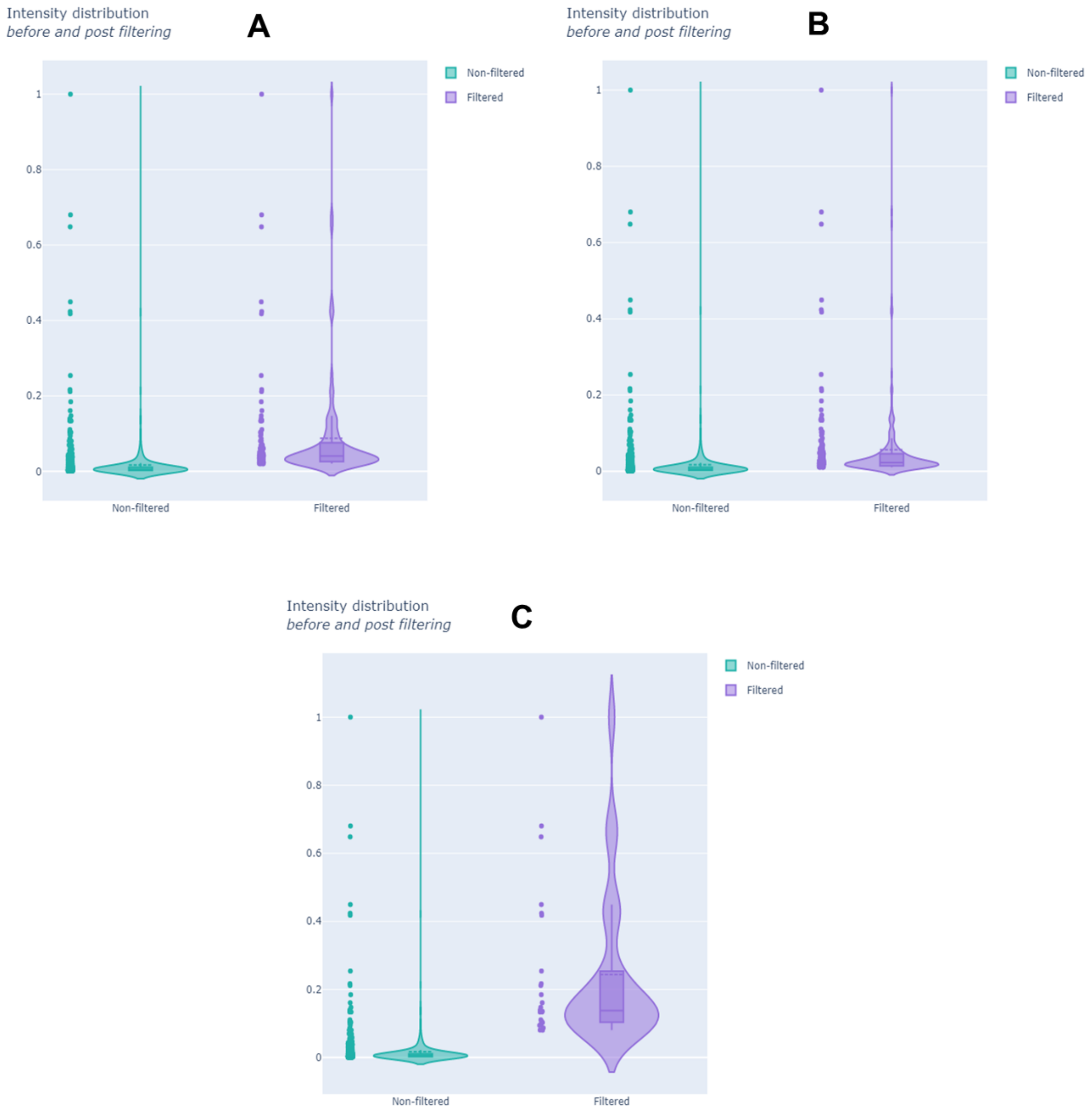

Supplementary Figure S83. Visualization of the effects of the filtering steps in a particular sample. A) Only the intensity-based filter was applied, all the values lower than 2% of relative intensity were minimized to zero [interactive figure here]. B) Only the quantile-based filter was applied, all the values lower than the third quantile (0.75) were minimized to zero [interactive figure here]. C) Effects of the sequential application of the intensity filter followed by the quantile-based filter [interactive figure here].

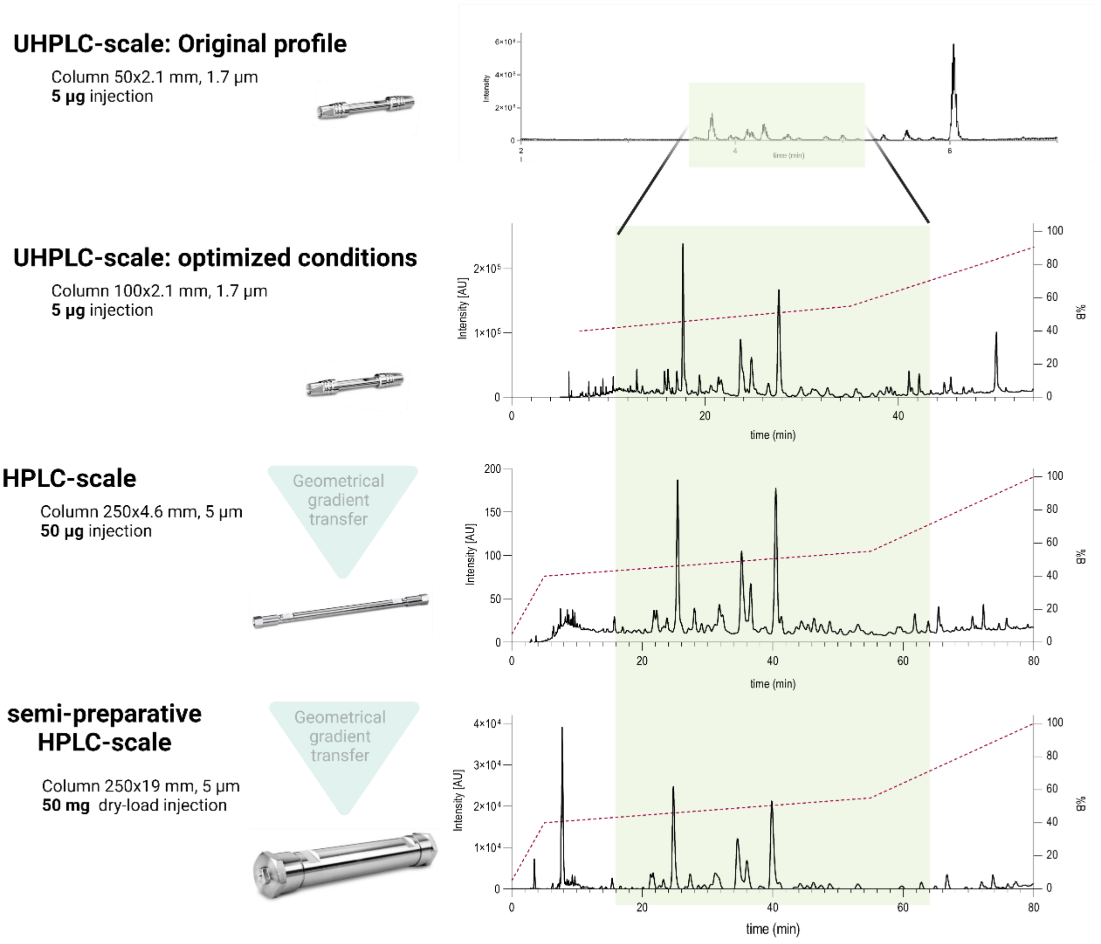

Supplementary Figure S84. Chromatograms at different analytical scales. A) UHPLC-254 nm, column Acquity BEH C_{18}, 100x2.1 mm, 1.7 μm. B) HPLC-254 nm, column Xbridge 250x4.6 mm, 5μm. C) Semi Preparative HPLC-254 nm, column Xbridge 250x19 mm, 5μm. The dashed line represents the %B [ACN + 0.1% Formic acid]. The region highlighted in light green indicates the region where the features spotted in the network are found.

Supplementary Table 2. Inventa Results for the Celastraceae Collection

Sample ID	FC	Feature specificity	MF prediction ratio	LC	Reported compounds in Species	Reported compounds in Genus	SC	CC	New CC in sp	New CC in genus	PR
LQ-01-61-78_pos.mzXML	0.82	0.84	0.32	1.00	2	8	1	1	{'Agarofuran sesquiterpenoids', 'Primary amides', 'Pyridine alkaloids'}	{'Agarofuran sesquiterpenoids', 'Primary amides', 'Pyridine alkaloids'}	3.82
LQ-01-61-37_pos.mzXML	0.81	0.84	0.54	1.00	1	440	1	1	{'Chromones', 'Simple coumarins', 'Isoquinoline alkaloids', 'Agarofuran sesquiterpenoids', 'Pyranocoumarins', 'Simple phenolic acids', 'Isocoumarins', 'Furocoumarins'}	{'Chromones', 'Simple coumarins', 'Isoquinoline alkaloids', 'Pyranocoumarins', 'Simple phenolic acids', 'Isocoumarins', 'Furocoumarins'}	3.81
LQ-01-61-06_pos.mzXML	0.84	0.86	0.48	0.89	212	732	1	1	{'Tetracyclic diterpenoids', 'Paraliane diterpenoids', 'Open-chain polyketides'}	{'Tetracyclic diterpenoids', 'Paraliane diterpenoids', 'Open-chain polyketides'}	3.73
LQ-01-61-07_pos.mzXML	0.70	0.71	0.50	0.96	71	732	1	1	{'Paraliane diterpenoids', 'Labdane diterpenoids', 'Neutral glycosphingolipids', 'Daucane sesquiterpenoids', 'Tetracyclic diterpenoids', 'Dicarboxylic acids', 'Trichothecane sesquiterpenoids', 'Monoacylglycerols', 'Diacylglycerols'}	{'Paraliane diterpenoids', 'Neutral glycosphingolipids', 'Daucane sesquiterpenoids', 'Tetracyclic diterpenoids', 'Dicarboxylic acids', 'Trichothecane sesquiterpenoids', 'Monoacylglycerols', 'Diacylglycerols'}	3.66
LQ-01-61-05_pos.mzXML	0.74	0.76	0.39	0.89	212	732	1	1	{'Pyridine alkaloids'}	{'Pyridine alkaloids'}	3.63
LQ-01-61-56_pos.mzXML	0.64	0.66	0.37	0.99	14	338	1	1	{'Paraliane diterpenoids', 'Agarofuran sesquiterpenoids', 'Drimane sesquiterpenoids', 'Daucane sesquiterpenoids'}	{'Paraliane diterpenoids', 'Agarofuran sesquiterpenoids', 'Drimane sesquiterpenoids', 'Daucane sesquiterpenoids'}	3.63
LQ-01-61-54_pos.mzXML	0.51	0.57	0.29	0.99	14	338	1	1	{'Agarofuran sesquiterpenoids', 'Trichothecane sesquiterpenoids', 'Lanostane, Tirucallane and Euphane triterpenoids'}	{'Agarofuran sesquiterpenoids', 'Trichothecane sesquiterpenoids', 'Lanostane, Tirucallane and Euphane triterpenoids'}	3.50
LQ-01-61-55_pos.mzXML	0.50	0.52	0.33	0.99	14	338	1	1	{'Agarofuran sesquiterpenoids', 'Trichothecane sesquiterpenoids', 'Drimane sesquiterpenoids', 'Daucane sesquiterpenoids'}	{'Agarofuran sesquiterpenoids', 'Trichothecane sesquiterpenoids', 'Drimane sesquiterpenoids', 'Daucane sesquiterpenoids'}	3.50
LQ-01-61-03_pos.mzXML	0.50	0.51	0.27	0.94	126	126	1	1	{'Ursane and Taraxastane triterpenoids', 'Tetraketide meroterpenoids', 'Triketide meroterpenoids', 'Daucane sesquiterpenoids'}	{'Ursane and Taraxastane triterpenoids', 'Daucane sesquiterpenoids', 'Tetraketide meroterpenoids', 'Triketide meroterpenoids'}	3.44
LQ-01-61-02_pos.mzXML	0.49	0.50	0.29	0.94	126	126	1	1	{'Oleanane triterpenoids'}	{'Oleanane triterpenoids'}	3.42
LQ-01-61-75_pos.mzXML	0.38	0.41	0.23	0.49	1011	1353	1	1	{'Vitamin D2 and derivatives'}	{'Vitamin D2 and derivatives'}	2.87
LQ-01-61-60_pos.mzXML	0.81	0.82	0.53	1.00	0	0	1	0			2.81

LQ-01-61-47_pos.mzXML	0.78	0.81	0.57	1.00	0	0	1	0			2.78
LQ-01-61-23_pos.mzXML	0.78	0.81	0.50	1.00	0	0	1	0			2.78
LQ-01-61-62_pos.mzXML	0.71	0.74	0.44	1.00	3	50	0	1	{'Dipeptides'}	{'Dipeptides'}	2.71
LQ-01-61-41_pos.mzXML	0.68	0.78	0.39	1.00	0	0	1	0			2.68
LQ-01-61-19_pos.mzXML	0.64	0.65	0.40	1.00	0	0	1	0			2.64
LQ-01-61-58_pos.mzXML	0.61	0.65	0.36	0.99	14	211	0	1	{'Ursane and Taraxastane triterpenoids', 'Agarofuran sesquiterpenoids'}	{'Ursane and Taraxastane triterpenoids', 'Agarofuran sesquiterpenoids'}	2.61
LQ-01-61-31_pos.mzXML	0.58	0.60	0.39	1.00	0	0	1	0			2.58
LQ-01-61-43_pos.mzXML	0.58	0.63	0.33	0.99	15	329	0	1	{'Flavones', 'Flavonols'}	{'Flavones', 'Flavonols'}	2.57
LQ-01-61-22_pos.mzXML	0.55	0.58	0.29	1.00	0	0	1	0			2.55
LQ-01-61-44_pos.mzXML	0.56	0.59	0.23	0.99	15	329	0	1	{'Lanostane, Tirucallane and Euphane triterpenoids', 'Pregnane steroids', 'Lupane triterpenoids'}	{'Lupane triterpenoids', 'Pregnane steroids', 'Lanostane, Tirucallane and Euphane triterpenoids'}	2.55
LQ-01-61-73_pos.mzXML	0.61	0.61	0.21	0.92	162	1353	1	0			2.53
LQ-01-61-72_pos.mzXML	0.49	0.50	0.41	1.00	0	0	1	0			2.49
LQ-01-61-53_pos.mzXML	0.45	0.49	0.28	0.98	43	329	0	1	{'Cholestane steroids'}	{'Cholestane steroids'}	2.43
LQ-01-61-52_pos.mzXML	0.43	0.45	0.32	0.98	43	329	0	1	{'Cholestane steroids'}	{'Cholestane steroids'}	2.41
LQ-01-61-01_pos.mzXML	0.46	0.49	0.27	0.94	126	126	1	0			2.40
LQ-01-61-16_pos.mzXML	0.44	0.50	0.29	0.95	92	440	0	1	{'Open-chain polyketides'}	{'Open-chain polyketides'}	2.39
LQ-01-61-04_pos.mzXML	0.45	0.46	0.31	0.94	126	126	1	0			2.38
LQ-01-61-67_pos.mzXML	0.51	0.57	0.37	0.84	313	514	0	1	{'Straight chain fatty acids', 'Lanostane, Tirucallane and Euphane triterpenoids'}	{'Straight chain fatty acids', 'Lanostane, Tirucallane and Euphane triterpenoids'}	2.36
LQ-01-61-66_pos.mzXML	0.50	0.52	0.33	0.84	313	514	0	1	{'Cholestane steroids', 'Lanostane, Tirucallane and Euphane triterpenoids'}	{'Cholestane steroids', 'Lanostane, Tirucallane and Euphane triterpenoids'}	2.34
LQ-01-61-30_pos.mzXML	0.35	0.37	0.25	0.95	92	440	0	1	{'Zearalenones', 'Dicarboxylic acids'}	{'Zearalenones', 'Dicarboxylic acids'}	2.30
LQ-01-61-71_pos.mzXML	0.42	0.49	0.25	0.84	313	514	0	1	{'Lanostane, Tirucallane and Euphane triterpenoids'}	{'Lanostane, Tirucallane and Euphane triterpenoids'}	2.26

LQ-01-61-74_pos.mzXML	0.51	0.53	0.29	0.49	1011	1353	1	0	2.01
LQ-01-61-27_pos.mzXML	0.83	0.90	0.51	0.99	11	440	0	0	1.83
LQ-01-61-28_pos.mzXML	0.76	0.81	0.63	1.00	1	440	0	0	1.76
LQ-01-61-42_pos.mzXML	0.71	0.71	0.41	1.00	0	0	0	0	1.71
LQ-01-61-57_pos.mzXML	0.71	0.82	0.38	0.99	14	211	0	0	1.70
LQ-01-61-70_pos.mzXML	0.67	0.68	0.28	1.00	0	0	0	0	1.67
LQ-01-61-09_pos.mzXML	0.70	0.70	0.19	0.96	71	732	0	0	1.66
LQ-01-61-08_pos.mzXML	0.70	0.70	0.48	0.96	71	732	0	0	1.66
LQ-01-61-68_pos.mzXML	0.66	0.66	0.34	1.00	0	0	0	0	1.66
LQ-01-61-33_pos.mzXML	0.65	0.68	0.50	1.00	0	0	0	0	1.65
LQ-01-61-40_pos.mzXML	0.64	0.65	0.42	1.00	0	0	0	0	1.64
LQ-01-61-69_pos.mzXML	0.63	0.74	0.37	1.00	0	0	0	0	1.63
LQ-01-61-51_pos.mzXML	0.62	0.65	0.50	1.00	0	0	0	0	1.62
LQ-01-61-14_pos.mzXML	0.61	0.62	0.35	1.00	0	0	0	0	1.61
LQ-01-61-15_pos.mzXML	0.65	0.71	0.32	0.95	92	440	0	0	1.60
LQ-01-61-63_pos.mzXML	0.59	0.63	0.43	1.00	0	0	0	0	1.59
LQ-01-61-12_pos.mzXML	0.57	0.61	0.40	1.00	0	0	0	0	1.57
LQ-01-61-32_pos.mzXML	0.56	0.59	0.37	1.00	0	0	0	0	1.56
LQ-01-61-46_pos.mzXML	0.55	0.57	0.43	1.00	0	0	0	0	1.55
LQ-01-61-59_pos.mzXML	0.55	0.56	0.37	0.99	14	211	0	0	1.54
LQ-01-61-29_pos.mzXML	0.58	0.62	0.33	0.95	92	440	0	0	1.54
LQ-01-61-64_pos.mzXML	0.53	0.57	0.43	1.00	0	0	0	0	1.53

LQ-01-61-61_pos.mzXML	0.48	0.52	0.33	1.00	0	0	0	0	1.48
LQ-01-61-20_pos.mzXML	0.46	0.46	0.28	1.00	2	440	0	0	1.46
LQ-01-61-35_pos.mzXML	0.45	0.51	0.29	1.00	0	0	0	0	1.45
LQ-01-61-45_pos.mzXML	0.45	0.49	0.32	1.00	0	0	0	0	1.45
LQ-01-61-11_pos.mzXML	0.45	0.49	0.29	1.00	0	0	0	0	1.45
LQ-01-61-10_pos.mzXML	0.44	0.50	0.35	1.00	0	0	0	0	1.44
LQ-01-61-18_pos.mzXML	0.44	0.44	0.28	1.00	0	0	0	0	1.44
LQ-01-61-21_pos.mzXML	0.43	0.46	0.24	1.00	0	0	0	0	1.43
LQ-01-61-38_pos.mzXML	0.42	0.43	0.28	1.00	1	440	0	0	1.42
LQ-01-61-34_pos.mzXML	0.42	0.42	0.22	1.00	0	0	0	0	1.42
LQ-01-61-36_pos.mzXML	0.40	0.41	0.26	1.00	0	0	0	0	1.40
LQ-01-61-26_pos.mzXML	0.39	0.42	0.24	1.00	0	0	0	0	1.39
LQ-01-61-17_pos.mzXML	0.38	0.40	0.15	1.00	0	0	0	0	1.38
LQ-01-61-39_pos.mzXML	0.38	0.38	0.28	1.00	1	440	0	0	1.38
LQ-01-61-50_pos.mzXML	0.38	0.41	0.22	1.00	0	0	0	0	1.38
LQ-01-61-13_pos.mzXML	0.37	0.42	0.29	1.00	0	0	0	0	1.37
LQ-01-61-24_pos.mzXML	0.37	0.40	0.27	1.00	0	0	0	0	1.37
LQ-01-61-48_pos.mzXML	0.36	0.44	0.18	1.00	0	0	0	0	1.36
LQ-01-61-49_pos.mzXML	0.33	0.33	0.15	1.00	0	0	0	0	1.33
LQ-01-61-25_pos.mzXML	0.30	0.31	0.22	1.00	0	0	0	0	1.30
LQ-01-61-65_pos.mzXML	0.29	0.30	0.24	0.84	313	514	0	0	1.13

Supplementary Table S3. Putative identities proposed for the extract of *Pristemira indica* roots.

cluster index	Exact mass (*m/z*)	Retention time (min)	Molecular Formula	Libname	Organism	Structure InChI
14749	768.3211	4.60	$C_{40}H_{49}NO_{14}$	ISDB (CAS 1403757-10-7)	Celastrus angulatus	InChI=1S/C40H49NO14/c1-21(2)33(44)49-20-39-30(53-36(47)26-17-13-14-18-41-26)27(50-23(5)42)19-38(9,48)40(39)31(51-24(6)43)28(37(7,8)55-40)29(52-34(45)22(3)4)32(39)54-35(46)25-15-11-10-12-16-25/h10-18,21-22,27-32,48H,19-20H2,1-9H3
8921	465.3001	6.04	$C_{30}H_{46}O_7$	CCMSLIB00004693704	Tripterygium wilfordi	InChI=1S/C30H40O4/c1-18-19-8-9-22-28(4,20(19)16-21(31)24(18)32)13-15-30(6)23-17-27(3,25(33)34-7)11-10-26(23,2)12-14-29(22,30)5/h8-9,16,23,32H,10-15,17H2,1-7H3/t23-,26-,27-,28+,29-,30+/m1/s1
95	421.2739	4.77	$C_{10}H_{14}N_4O_4$	CCMSLIB00000078862	Maytenus	InChI=1S/C28H36O3/c1-16-13-23-25(3,15-21(16)30)9-11-27(5)22-8-7-18-17(2)24(31)20(29)14-19(18)26(22,4)10-12-28(23,27)6/h7-8,14,16,23,31H,9-13,15H2,1-6H3/t16-,23?,25+,26+,27-,28+/m1/s1
